# Impaired NADH-linked mitochondrial respiration disrupts ventral midbrain neuronal programs in POLG disease

**DOI:** 10.1186/s12967-026-08706-w

**Published:** 2026-07-31

**Authors:** Anbin Chen, Tsering Yangzom, Gareth John Sullivan, Kristina Xiao Liang

**Affiliations:** 1 Department of Neurosurgery, Xinhua Hospital, Shanghai Jiao Tong University School of Medicine, Shanghai, 200092 China; 2 Department of Biomedicine (IBM), Faculty of Medicine, University of Bergen, Bergen, 5020 Norway; 3 Department of Pediatric Research, Oslo University Hospital, Oslo, 0372 Norway; 4 School of Medicine, Medical & Biological Sciences, University of St Andrews, North Haugh, St Andrews, KY16 9TF UK

**Keywords:** POLG disease, Dopaminergic vulnerability, NADH-dependent respiration, Midbrain organoids, Single-cell RNA sequencing

## Abstract

**Background:**

POLG (DNA polymerase γ catalytic subunit)-related mitochondrial diseases are among the most severe primary mitochondrial disorders and are characterized by progressive neurodegeneration with prominent dopaminergic involvement. However, the cell type-specific mechanisms linking mitochondrial DNA instability to neuronal vulnerability remain incompletely defined.

**Methods:**

Using patient-derived midbrain organoids and single-cell RNA sequencing, we investigated how *POLG* mutations alter mitochondrial and neuronal programs at subtype resolution. We analyzed dopaminergic neuronal populations and ventral midbrain neurons to define disease-associated transcriptional changes. To evaluate therapeutic improvement, POLG organoids were treated chronically with nicotinamide riboside (NR), followed by single-cell transcriptomic profiling and pathway enrichment analysis.

**Results:**

*POLG* mutations induced a coordinated downregulation of genes associated with oxidative phosphorylation and synaptic signaling, particularly in terminally differentiated dopaminergic neurons. This transcriptional alteration involved genes encoding respiratory chain complexes I–V, mitochondrial translation machinery, and ATP synthase components, suggesting disruption of mitochondrial bioenergetic programs at the transcriptomic level. Among dopaminergic subtypes, DA2 neurons and ventral midbrain neurons showed the most pronounced transcriptional alterations, indicating maturation-dependent vulnerability. NR treatment was associated with altered expression of genes involved in oxidative phosphorylation, NADH dehydrogenase activity, respiratory chain assembly, and synaptic pathways. Following NR exposure, dopaminergic subpopulations exhibited changes in cell-type proportions and partial normalization of mitochondrial- and synaptic-related transcriptional programs.

**Conclusions:**

These findings identify transcriptional alterations in pathways related to mitochondrial respiration. The data further suggests that modulation of NAD⁺ metabolism is associated with transcriptional changes in mitochondrial and neuronal pathways in this disease context.

**Graphical Abstract:**

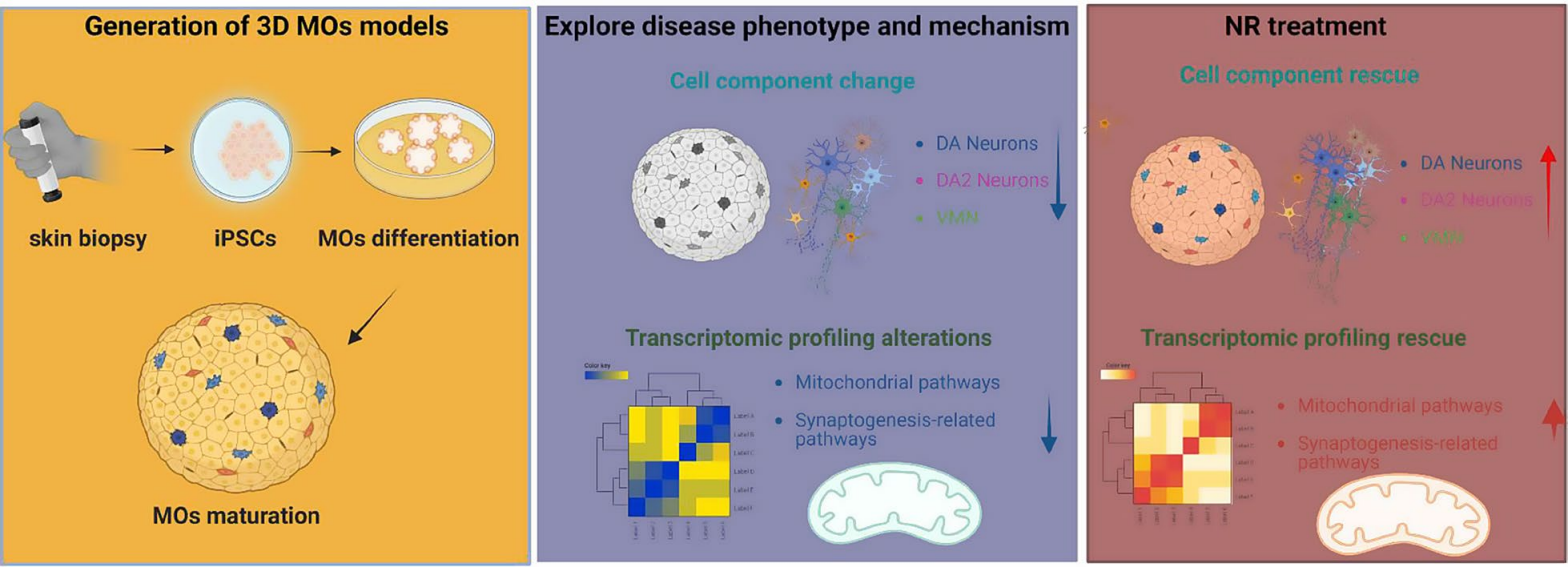

**Supplementary Information:**

The online version contains supplementary material available at 10.1186/s12967-026-08706-w.

## Introduction

POLG-related mitochondrial diseases represent some of the most severe forms of primary mitochondrial disorders, frequently characterized by progressive neurodegeneration, epilepsy, and movement disorders, including Parkinsonian features [1–3]. Mutations in the *POLG* gene impair mitochondrial DNA (mtDNA) replication and maintenance, leading to mtDNA depletion, defective respiratory chain assembly, and compromised oxidative phosphorylation [1–3]. Neurons, with their exceptionally high bioenergetic demand, are particularly vulnerable to mitochondrial dysfunction, and dopaminergic (DA) neurons appear especially sensitive in POLG-associated disorders [4]. However, the cellular and molecular mechanisms that link mtDNA instability to selective dopaminergic vulnerability remain incompletely defined.

Mitochondrial dysfunction has long been implicated in neurodegenerative diseases, including Parkinson’s disease [5]. DA neurons of the midbrain rely heavily on NADH-dependent mitochondrial respiration to sustain pacemaking activity, synaptic transmission, and extensive axonal arborization [6, 7]. Disruption of mitochondrial electron transport, particularly at complex I, can lead to redox imbalance, ATP insufficiency, and oxidative stress, thereby compromising neuronal integrity [8–10]. While *POLG* mutations are known to disrupt mtDNA maintenance [1, 3], it remains unclear whether they induce coordinated failure of specific mitochondrial respiratory programs within defined dopaminergic subpopulations, and how such failure relates to synaptic dysfunction and neuronal loss.

The emergence of human pluripotent stem cell–derived brain organoids has provided powerful platforms to model region-specific neurodevelopment and disease mechanisms in a three-dimensional context [11–17]. Midbrain-like organoids can recapitulate key aspects of DA neuron development and subtype diversity [15, 18, 19], offering opportunities to investigate selective neuronal vulnerability at single-cell resolution. Combined with single-cell RNA sequencing (scRNA-seq) technologies [19–21], these systems allow dissection of cell-type-specific transcriptional alterations in mitochondrial and synaptic pathways that may underline disease pathogenesis.

In parallel, growing evidence suggests that modulation of cellular NAD⁺ metabolism may counteract mitochondrial dysfunction and neurodegeneration [22, 23]. Nicotinamide riboside (NR), a precursor of NAD⁺, has been shown to enhance mitochondrial function and support neuronal survival in experimental systems [24–26]. Whether NAD⁺ boosting can improve mitochondrial respiratory programs and associate synaptic transcriptional networks in *POLG*-mutant DA neurons, however, remains to be fully elucidated.

Here, using patient-derived midbrain organoids (MOs) and single-cell transcriptomic profiling, we investigate how *POLG* mutations reshape mitochondrial and neuronal programs at subtype resolution. We identify a coordinated disruption of NADH-dependent mitochondrial respiration in DA neurons, tightly coupled to suppression of synaptic gene expression. Furthermore, we assessed whether boosting NAD⁺ metabolism with nicotinamide riboside can enhance these transcriptional programs. Together, this study defines a mechanistic link between mtDNA instability, respiratory failure, and selective dopaminergic vulnerability in POLG disease.

## Methods

### Culture of human induced pluripotent stem cells (iPSCs)

Patient-derived iPSCs were generated from fibroblasts carrying the *POLG* mutation c.2243G > C (p.W748S; WS5A) as previously described [4, 23]. The fibroblast line Detroit 551(ATCC® CCL 110™, human normal fetal female fibroblast) was reprogrammed to generate disease-free control iPSCs. In addition, isogenic control iPSCs were generated by CRISPR–Cas9-mediated correction of the pathogenic POLG mutation and usedas genetically matched disease-free controls. Briefly, ribonucleoprotein (RNP) complexes consisting of Cas9 protein and synthetically modified single-guide RNAs (sgRNAs) (Synthego, Redwood City, CA, USA) were introduced into cells via electroporation together with a single-stranded oligodeoxynucleotide (ssODN) donor template. Editing efficiency was initially evaluated after a 48-h recovery period by PCR amplification and Sanger sequencing of the target locus. Sequencing traces were analyzed using Inference of CRISPR Edits (ICE, Synthego) software. Monoclonal cell populations were generated by single-cell isolation and screened using PCR-Sanger sequencing to confirm correction of the target mutation. The human embryonic stem cell (ESC) line 360 [23] was used as an internal control for MO differentiation.

iPSCs and ESCs were maintained on Geltrex-coated plates (Geltrex™ LDEV-Free, hESC-qualified, Thermo Fisher Scientific, Cat# A1413302) in Essential 8 medium (Thermo Fisher Scientific, Cat# A1517001) supplemented with ROCK inhibitor Y-27632 (10 µM; Tocris Bioscience, Cat# 1254) for the first 24 h post-plating. Medium was changed daily, and cells were passaged at 70–80% confluence using 0.5 mM EDTA (Thermo Fisher Scientific, Cat# 15575020). All cultures were routinely tested for mycoplasma contamination using the MycoAlert™ Mycoplasma Detection Kit (Lonza, Cat# LT07-318).

### Generating and culturing MOs

MOs were generated using a modified dual-SMAD inhibition protocol for neural induction followed by midbrain floor-plate patterning. For neural induction, iPSCs at ~ 70% confluence were cultured in Essential 8 medium (Thermo Fisher Scientific, Cat# A1517001) and subsequently switched to neural induction medium consisting of Chemically Defined Medium (CDM) supplemented with SB431542 (10 µM; Tocris Bioscience, Cat# 1614), N-acetyl-L-cysteine (NAC; 10 µM; Sigma-Aldrich, Cat# A9165), and dorsomorphin (Compound C; 2 µM; Sigma-Aldrich, Cat# P5499). CDM consisted of IMDM (Thermo Fisher Scientific, Cat# 12440053) and Ham’s F12 (Thermo Fisher Scientific, Cat# 11765054) mixed at a 1:1 ratio, supplemented with BSA (0.5% w/v; Sigma-Aldrich, Cat# A9647), Lipid Concentrate 100 × (1×; Thermo Fisher Scientific, Cat# 11905031), thioglycerol (450 µM; Sigma-Aldrich, Cat# M6145), insulin (7 µg/mL; Sigma-Aldrich, Cat# I9278), and transferrin (7.5 µg/mL; Sigma-Aldrich, Cat# T8158).

On day 5, neural epithelial structures were detached using collagenase IV (1 mg/mL; Thermo Fisher Scientific, Cat# 17104019) and transferred to ultra-low attachment plates (Corning, Cat# 3471) to form neurospheres. From day 5–12, spheres were maintained in CDM supplemented with recombinant human FGF8b (100 ng/mL; PeproTech, Cat# 100−25). From day 12, Purmorphamine (PM; 1 µM; Calbiochem, Cat# 540220) and FGF8b (100 ng/mL) were added to promote ventral midbrain floor plate specification. At day 19, organoids were embedded in growth factor–reduced Matrigel (Corning, Cat# 356231) and matured in CDM supplemented with recombinant human BDNF (20 ng/mL; PeproTech, Cat# 450-02) and recombinant human GDNF (20 ng/mL; PeproTech, Cat# 450−10) for up to 4 months. For each experiment, 3–6 organoids were generated per differentiation batch. At least 3 independent differentiation batches were performed. Organoids with regular morphology and comparable diameters were selected for downstream analyses.

### Midbrain dopaminergic (DA) neuron differentiation

For DA neuron differentiation, MOs were dissociated into single cells using TrypLE™ Express Enzyme (Thermo Fisher Scientific, Cat# 12604013) and plated onto poly-L-ornithine (Sigma-Aldrich, Cat# P4957) and laminin (Sigma-Aldrich, Cat# L2020)–coated plates.

Neurons were matured in CDM supplemented with recombinant human BDNF (10 ng/mL) and recombinant human GDNF (10 ng/mL).

### NR treatment

MOs were treated daily with 1 mM NR for two months. NR was provided by Prof. Evandro Fei Fang (University of Oslo). NR concentration (1 mM) was selected based on our previous findings [24] showing beneficial effects at 0.5 mM in 2D POLG-derived cell cultures, with a moderate increase applied to account for diffusion limitations in 3D organoid systems. NR treatment was initiated on day 30 of organoid differentiation and continued daily for two months. Control organoids received equivalent volumes of vehicle under identical experimental conditions.

### Immunostaining of iPSCs and DA neurons

iPSCs and DA neurons were fixed in 4% paraformaldehyde (PFA; VWR, Cat# 100503-917) for 10 min at room temperature and blocked in PBS containing 10% normal goat serum (Sigma-Aldrich, Cat# G9023) and 0.3% Triton™ X-100 (Sigma-Aldrich, Cat# X100-100ML). Cells were incubated overnight at 4 °C with primary antibodies against pluripotency markers (SOX2, OCT4, SSEA4, NANOG) or neuronal markers (TH, TUJ1, MAP2, Synaptophysin, PSD95). After washing with PBS, samples were incubated with species-appropriate Alexa Fluor–conjugated secondary antibodies (1:400; Thermo Fisher Scientific) for 1 h at room temperature in the dark. Nuclei were counterstained with DAPI (Thermo Fisher Scientific, Cat# D1306), and samples were mounted using Fluoromount-G (SouthernBiotech, Cat# 0100−20). Antibody details, including host species and dilution factors, are provided in Table S1.

### Immunostaining of MOs

Organoids were fixed in 4% PFA for 30 min at room temperature, washed in PBS, and cryoprotected in 20% sucrose (Sigma-Aldrich, Cat# S0389) in PBS overnight at 4 °C. Organoids were then mounted onto coverslips and allowed to air-dry prior to immunostaining. Samples were blocked and permeabilized in PBS containing 10% normal goat serum and 0.1% Triton™ X-100 for 2 h at room temperature. Organoids were incubated with primary antibodies diluted in blocking buffer overnight at 4 °C. Following three washes in PBS (10 min each), samples were incubated with species-appropriate Alexa Fluor–conjugated secondary antibodies (1:400; Thermo Fisher Scientific) overnight at 4 °C in the dark. Nuclei were counterstained with DAPI, and coverslips were mounted using Fluoromount-G. Detailed antibody information, including host species and dilution factors, is provided in Table S1.

### Microscopy and image quantification

Images were acquired using a Leica TCS SP8 confocal laser scanning microscope (Leica Microsystems, Wetzlar, Germany) equipped with LAS X software. Images were captured using identical acquisition settings (laser power, gain, and offset) across experimental groups to ensure comparability. Image processing and analysis were performed using Fiji (ImageJ, NIH, USA). For quantitative analysis, 6–10 randomly selected regions of interest (ROIs) per sample were analyzed. All images were converted to 8-bit format, and fluorescence intensity was measured using standardized threshold settings applied uniformly across all samples. Fluorescence intensity was quantified as mean gray value, calculated as integrated density divided by area. Analyses were performed in a blind manner where applicable. Statistical analyses were conducted using GraphPad Prism 8.0.2 (GraphPad Software, San Diego, CA, USA).

### Flow cytometry analysis

Cells were dissociated using TrypLE™ Express Enzyme (Thermo Fisher Scientific, Cat# 12604013), washed in PBS, and fixed in 1.6% paraformaldehyde for 10 min at room temperature. Cells were then permeabilized with ice-cold 90% methanol (Sigma-Aldrich, Cat# 34860) for 20 min at − 20 °C. After washing, cells were blocked in PBS containing 0.3 M glycine (Sigma-Aldrich, Cat# G7126), 5% normal goat serum (Sigma-Aldrich, Cat# G9023), and 1% bovine serum albumin (BSA; Sigma-Aldrich, Cat# A9647) for 30 min at room temperature. Cells were incubated with primary antibodies against tyrosine hydroxylase (TH) and dopamine transporter (DAT) overnight at 4 °C, followed by species-appropriate Alexa Fluor–conjugated secondary antibodies (1:400; Thermo Fisher Scientific) for 1 h at room temperature in the dark. Flow cytometric analysis was performed using a BD Accuri™ C6 flow cytometer (BD Biosciences, Franklin Lakes, NJ, USA). At least 40,000 events were recorded per sample. Doublets were excluded using FSC-H versus FSC-A and SSC-H versus SSC-A gating strategies. Data were analyzed using BD Accuri™ C6 software . Antibody details are provided in Table S1.

### Electrophysiological assessment

Electrophysiological assessments were conducted using whole-cell patch-clamp techniques to record DA neurons derived from control iPSCs, following the methodology outlined in the previous paper [4].

### ScRNA-seq analysis

Organoids were washed once in PBS (Thermo Fisher Scientific, Cat# 10010023) and mechanically minced into 1–2 mm fragments using sterile ophthalmic scissors. Tissue fragments were transferred to 15 mL centrifuge tubes (Sarstedt, Cat# 62.554.001) and digested in 2 mL CellLive™ Tissue Dissociation Solution (Singleron Biotechnologies, Cat# 1190062) at 37 °C for 15 min with continuous agitation on a heated shaker (300 rpm). Following enzymatic digestion, the cell suspension was filtered through a 40 μm sterile cell strainer (Greiner Bio-One, Cat# 542040) and centrifuged at 350 × g for 5 min at 4 °C. The resulting pellet was resuspended in 1 mL PBS. Cell viability and concentration were assessed using 0.4% trypan blue solution (Gibco, Cat# 15250-061) and quantified with a hemocytometer. Only samples with viability > 85% were used for downstream single-cell library preparation. For each condition, three independent organoid differentiations were generated. Organoids were dissociated and single-cell suspensions from the three biological replicates were pooled prior to library preparation and sequencing.

Single-cell libraries were generated using the GEXSCOPE single-cell RNA-seq Library Kit (Singleron Biotechnologies) according to the manufacturer’s instructions and sequenced on an Illumina NovaSeq 6000 platform using paired-end 2 × 150 bp sequencing. Raw FASTQ files were processed using CeleScope^®^ (v1.14.1; Singleron Biotechnologies GmbH) with default parameters. Low-quality reads were removed prior to alignment using STAR against the human reference genome GRCh38 with Ensembl 92 annotation. Gene-level counts were generated using featureCounts, and cell calling was performed by fitting a negative bimodal distribution to distinguish cell-containing droplets from empty droplets, generating a UMI count matrix.

Initial preprocessing and quality-control filtering were performed using Scanpy [27] implemented in Python. Downstream analyses were conducted using Scanpy [27] in Python and Seurat in R. Quality control metrics included number of detected genes per cell (nFeature_RNA) and percentage of mitochondrial transcripts (percent_mt). Cells with > 20% mitochondrial reads, < 200 detected genes, > 6,500 genes, or > 30,000 UMIs were excluded to remove low-quality cells and potential doublets.

Data integration was performed using Harmony [28] within the Scanpy pipeline. The three datasets (Isogenic MOs , POLG MOs, and POLG MOs + NR treatment) were integrated and analyzed jointly to minimize technical variation across datasets prior to downstream analyses. The number of cells retained following quality-control filtering and sequencing metrics for each sample are summarized in Supplementary Table S2. Neighborhood graphs were computed using scanpy.pp.neighbors (n_neighbors = 10, n_pcs = 10), followed by unsupervised clustering using the Leiden algorithm (scanpy.tl.leiden, resolution = 1). Clusters were annotated based on canonical marker gene expression from published datasets [18–21, 29–31]. DA progenitors were defined by expression of *NEUROG2*,* NHLH1*, and *SOX4* [20, 29]. DA0 neurons were identified by high *ERBB4* and low *SYT1* expression [30]. DA1/2 neurons were defined by expression of *NR4A2*,* DCX*, and *SYT1* [18, 31]. Neural progenitors were annotated based on *TOP2A*,* MKI67*, and HMGB2 expression. Oligodendrocytes were identified by *RBFOX1* [21], oligodendrocyte progenitors by *OLIG2*, astrocytes by *GFAP*,* AQP4*,* EDNRB*, and *RFX4*, radial glial cells by *FABP7*, and proliferative glial progenitors by *TOP2A* and *MKI67* [19, 31]. Meningeal cells were defined by *COL1A1*,* COL1A2*, and *LUM* expression. Ventral midbrain neurons (VMNs) were annotated using CellTypist [32] with reference to develop human brain datasets [33]. We have clarified that VMNs were annotated using a reference-based approach (CellTypist with developing human brain datasets) and therefore represent a transcriptionally defined population rather than a strictly anatomically purified midbrain cell type. As a result, some enrichment signatures may reflect shared developmental transcriptional programs across closely related neuronal lineages or limitations in reference-based annotation resolution. For all scRNA-seq analyses, the CRISPR-corrected isogenic control served as the reference control for differential expression and downstream analyses.

A relatively permissive fold-change threshold (|log2FC| > 0.25) was applied to identify biologically coordinated transcriptional changes in scRNA-seq datasets while controlling for multiple testing using FDR < 0.05. Differential expression analysis was performed using Scanpy [27] across identified cell clusters and between defined cell populations. The Wilcoxon rank-sum test was applied with Benjamini–Hochberg correction for multiple testing. Cluster-specific cell proportions and absolute cell counts were calculated at the sample level. For selected analyses, clusters were combined into biologically relevant groups prior to differential testing.

Gene Ontology (GO) and Kyoto Encyclopedia of Genes and Genomes (KEGG) enrichment analyses were performed on significantly upregulated and downregulated genes using the Python package gseapy (gseapy.enrichr), which interfaces with the Enrichr platform [34]. The following databases were used: GO_Biological_Process_2021, *GO_Molecular_Function_2021*, *GO_Cellular_Component_2021*, *Reactome_2016, and KEA_2015*. Pathways with adjusted P values below the predefined significance threshold were considered significantly enriched. Pathways with adjusted p-values below the predefined significance threshold were considered significantly enriched.

### Statistical analysis

For all experiments, organoids were generated from at least three independent differentiations per condition. For scRNA-seq analyses, cells from three independent organoid differentiations were pooled prior to library preparation and sequencing, and the CRISPR-corrected isogenic control served as the reference control for all differential expressions and downstream transcriptomic analyses. Unless otherwise specified, non-transcriptomic experiments were performed using both Detroit 551-derived control organoids and CRISPR-corrected isogenic control organoids, which were combined and presented as the control group. Data were presented as mean ± standard deviation (SD) from at least three independent experiments. Normality was assessed using the Shapiro–Wilk test, and outliers were identified using the ROUT method. For normally distributed data, two-sided Student’s t-tests were applied; otherwise, the Mann–Whitney U test was used. Statistical analyses were performed using GraphPad Prism 8.0.2. A p-value ≤ 0.05 was considered statistically significant.

### Artificial intelligence statement

The authors used ChatGPT (OpenAI, GPT-4) for language editing and improvement of manuscript clarity. No scientific content, data analysis, interpretation, or conclusions were generated by artificial intelligence. All final content was reviewed and approved by the authors.

## Results

### Generation and characterization of MOs derived from iPSCs and ESCs

**Fig. 1:**
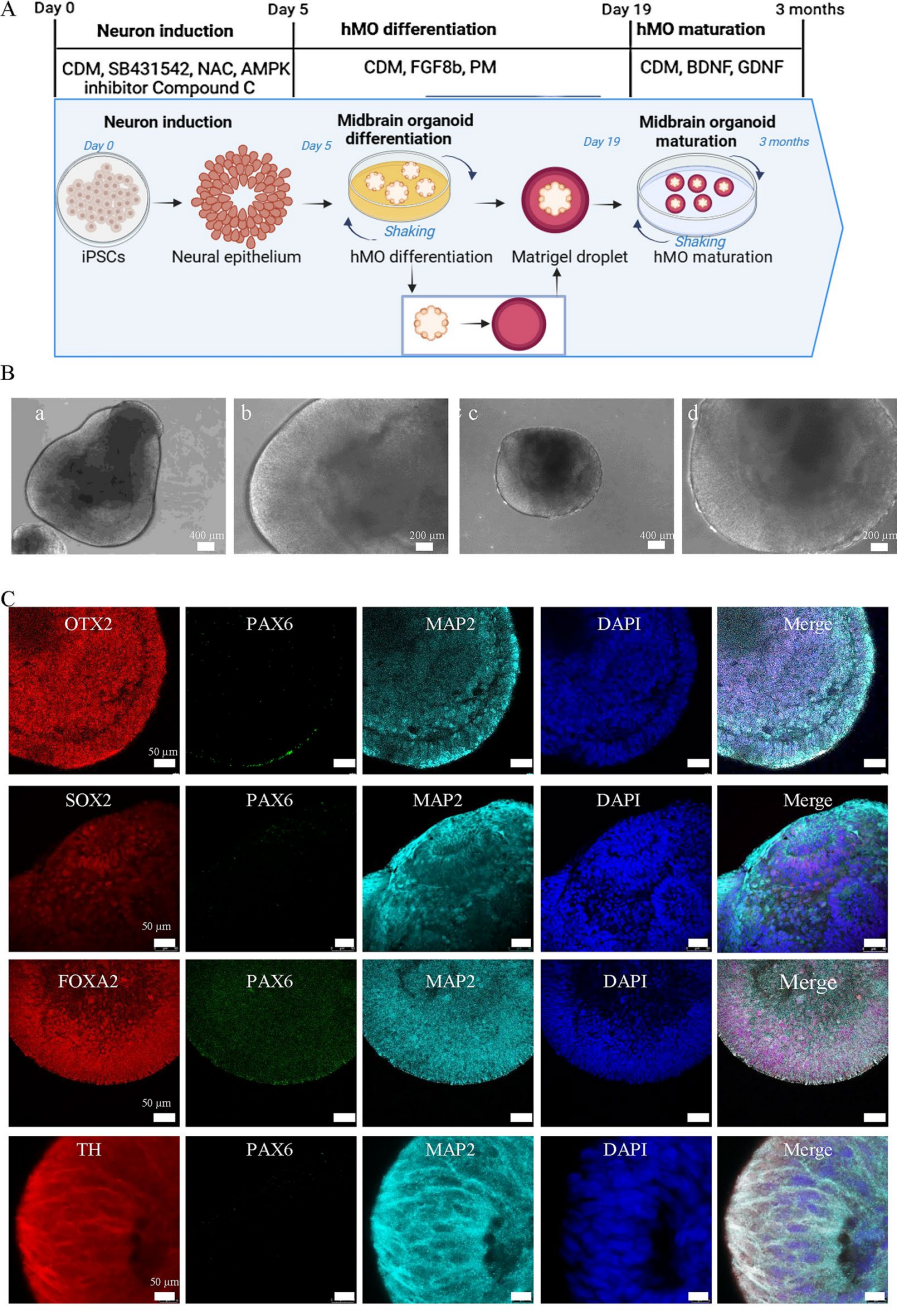
Generation and characterization of MOs from iPSCs and ESCs. (**A**) Schematic overview of the differentiation protocol used to generate MOs from control iPSCs and ESCs, created using BioRender.com. (**B**) Representative phase-contrast images of 2-month-old MOs derived from ESCs (a–b; 10× and 20× magnification) and control iPSCs (c–d; 10× and 20× magnification). Scale bars, 400 μm and 200 μm. (**C**) Immunofluorescence staining of 2-month-old MOs showing expression of midbrain and neural markers. Combinations include OTX2/PAX6/MAP2, SOX2/PAX6/MAP2, FOXA2/PAX6/MAP2, and TH/PAX6/MAP2. Nuclei are counterstained with DAPI (blue). Scale bars, 50 μm

Human iPSCs expressing canonical pluripotency markers NANOG, OCT4, and SOX2 (Fig. S1) were differentiated into MOs using a dual-SMAD inhibition protocol supplemented with FGF8b and the SHH agonist PM (Fig. 1A). Neuroectodermal specification toward a floor plate fate was induced through dual-SMAD inhibition in the presence of NAC, followed by suspension culture to generate 3D neurospheres. Caudalization with FGF8b and subsequent ventralization with FGF8b and PM promoted midbrain patterning. Organoids were embedded in Matrigel and matured in the presence of BDNF and GDNF (Fig. 1A).

Both ESCs and iPSCs (Fig. S2, a) efficiently progressed through neuroepithelial stages (Fig. S2, b) and DA progenitor specification (Fig. S2, c). Organoid formation was evident by day 19 (Fig. S2, d), and structural maturation was achieved by day 50 in both control iPSC and ESC-derived cultures (Fig. S2, e and f). Comparable morphology was observed between ESC- and iPSC-derived organoids after two months of differentiation (Fig. 1B).

To confirm regional identity, 60-day organoids were assessed for midbrain markers. Low expression of the forebrain marker PAX6 and robust expression of OTX2 and FOXA2 confirmed midbrain floor plate specification (Fig. 1C). TH expression showed the DA neuron lineage, and SOX2-positive neural progenitors and MAP2-positive mature neurons were detected within the same organoids, indicating neuronal identity (Fig. 1C). Midbrain dopaminergic identity was further validated by TH expression. TH co-localized with MAP2 at day 60, consistent with progressive neuronal maturation (Fig. 1C). Quantitative fluorescence analysis demonstrated comparable expression levels between ESC- and iPSC-derived organoids (Fig. S3). Expression level of OTX2 was higher in iPSC derived MOs, while the FOXA2, SOX2, and TH were similar between ESC- and iPSC-derived organoids (Fig. S3).

Together, these data establish that both ESC- and iPSC-derived cultures reproducibly generate structurally organized MOs with appropriate floor plate specification and DA neuron differentiation.

### Neuronal differentiation of ESC- and iPSC-derived MOs

To evaluate dopaminergic maturation, two-month-old organoids were dissociated into single cells and cultured for an additional four weeks. Dissociated cultures formed extensive neuronal networks with elongated neurites (Fig. S4A). Flow cytometry demonstrated that the majority of cells were tyrosine TH-positive (98.2%), and a substantial fraction expressed dopamine DAT (76.6%) (Fig. S4B, C). Immunostaining confirmed co-expression of TH and MAP2, and positive expression of the DAT, consistent with midbrain DA neuron identity (Fig. S4C). Synaptic maturation was assessed using pre- and postsynaptic markers. Neurons expressed synaptophysin and PSD-95, with clear co-localization patterns indicative of synaptic protein organization (Fig. S4D).

Functional properties were evaluated using electrophysiological recordings. Neurons displayed spontaneous and evoked action potentials with regular firing patterns (Fig. S5B), consistent with electrically active DA neurons.

Together, these findings demonstrate that ESC- and iPSC-derived MOs generate mature, synaptically competent, and electrophysiologically active DA neurons suitable for downstream disease modeling.

### POLG organoids exhibit reduced dopaminergic representation and coordinated transcriptional suppression

**Fig. 2:**
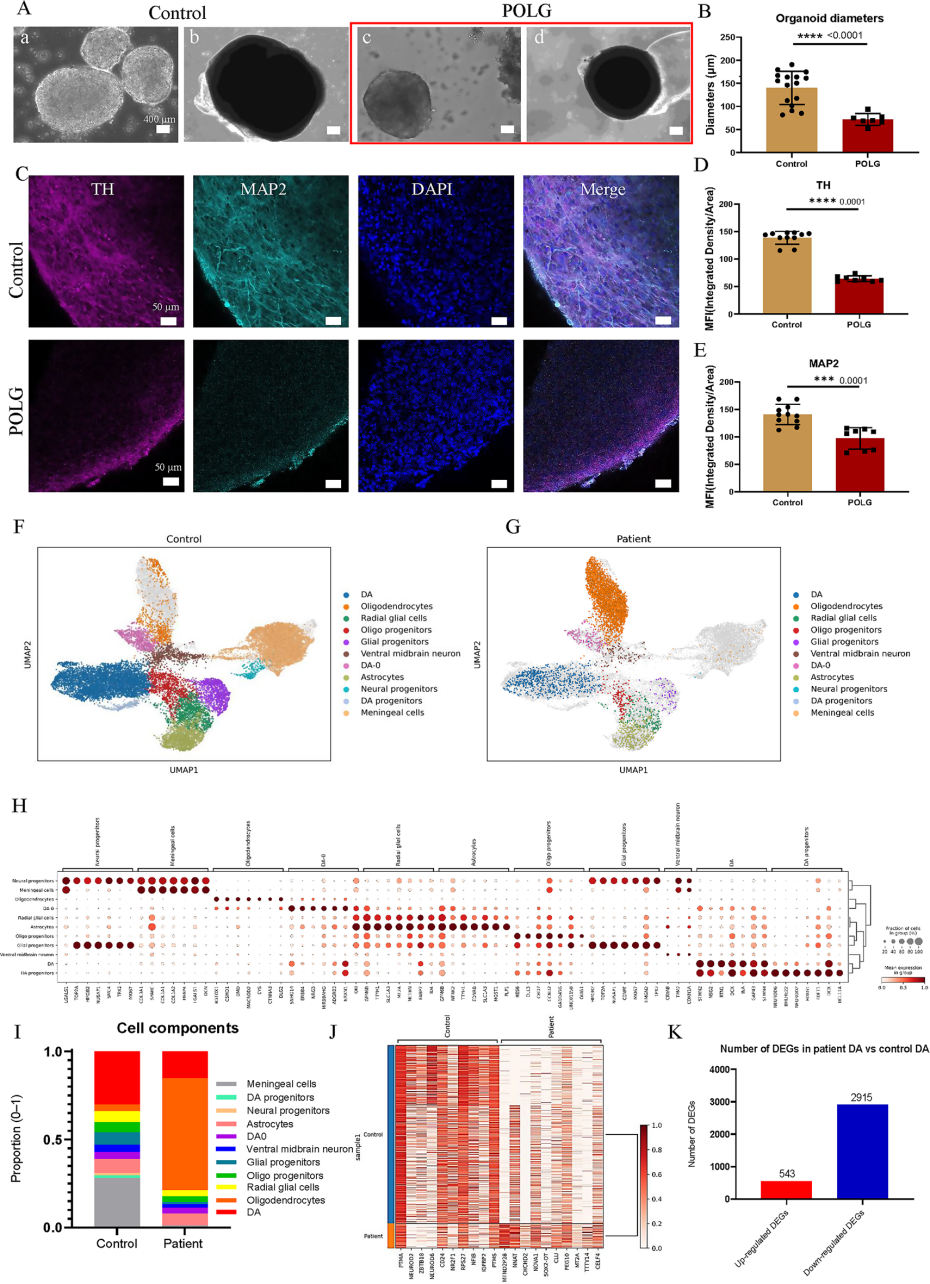
Differentiation and phenotypic comparison of 90-day MOs derived from POLG iPSCs versus controls. (**A**-**B**) Representative phase-contrast images (**A**) and measurement of the organoid diameters (**B**) of 90-day MOs derived from control and POLG patient iPSCs. Scale bar, 400 μm. (**C**–**E**) Immunofluorescence staining and quantification for TH and MAP2 in 90-day control and POLG MOs. Nuclei are stained with DAPI (blue). Scale bar, 100 μm. (**F**–**G**) UMAP visualization and quantitative analysis of cellular composition in 90-day control and POLG MOs. (**H–I**) Differential gene expression analysis and cell proportion comparisons across annotated cell populations in 90-day MOs. (**J**–**K**) Heatmap and numbers of upregulated and downregulated DEGs in 90-day POLG MOs compared to controls.S scRNA-seq analysis was performed on pooled cells derived from three independent organoid differentiations per condition

To model POLG-associated mitochondrial disease, patient-specific iPSC-derived MOs were generated using the same differentiation protocol. Compared with isogenic controls, POLG organoids displayed irregular morphology and significantly reduced size (Fig. 2A, B). Immunostaining of 90-day MOs revealed marked reduction in TH and MAP2 expressions in POLG organoids compared to controls (Fig. 2C–E), indicating preferential impairment of DA neuron differentiation. scRNA-seq was performed to define cellular and molecular alterations. Both control (5,369 cells, Fig. 2F and Table S2) and POLG organoids (3,331 cells, Fig. 2G and Table S2) contained comparable major cell populations, including DA progenitors, DA neurons (DA0, DA1, DA2), VMNs, astrocytes, oligodendrocytes, radial glia, and DA progenitor populations (Fig. 2F, G) with the marker gene clusters (Fig. 2H). However, the proportion of DA neurons was significantly reduced in POLG organoids (15.25%) compared with controls (30.2%) (Fig. 2I), confirming selective vulnerability of DA populations. Differential expression analysis identified widespread transcriptional suppression in POLG organoids, with 2,915 downregulated and 543 upregulated genes (Fig. 2J, K). GO enrichment of downregulated genes demonstrated significant suppression of synaptic processes, including trans-synaptic signaling, synaptic vesicle cycle, and synapse organization (Fig. S6A).

Cellular component analysis further highlighted enrichment of synaptic membrane, postsynaptic membrane, and glutamatergic synapse categories, alongside mitochondrial inner membrane and mitochondrial protein complex components (Fig. S6B). Molecular function analysis revealed downregulation of glutamate receptor binding, ion channel binding, clathrin binding, and oxidoreductase activity acting on NAD(P)H substrates (Fig. S6C, S9). Network visualization (emapplot) identified modulation of chemical synaptic transmission and oxidative phosphorylation as central interconnected nodes among the suppressed pathways (Fig. S7-S9).

Together, these findings demonstrate that *POLG* mutation leads to the reduced DA neurons representation and coordinated suppression of synaptic and mitochondrial transcriptional programs in MOs.

### DA neurons in POLG organoids exhibit mitochondrial respiratory and synaptic transcriptional suppression

To define cell-type-specific mechanisms, differential expression analysis was performed in DA neuron’s derived from POLG organoids. Two major axes of dysfunction emerged: suppression of mitochondrial bioenergetic programs and impairment of neuronal signaling pathways.

**Fig. 3:**
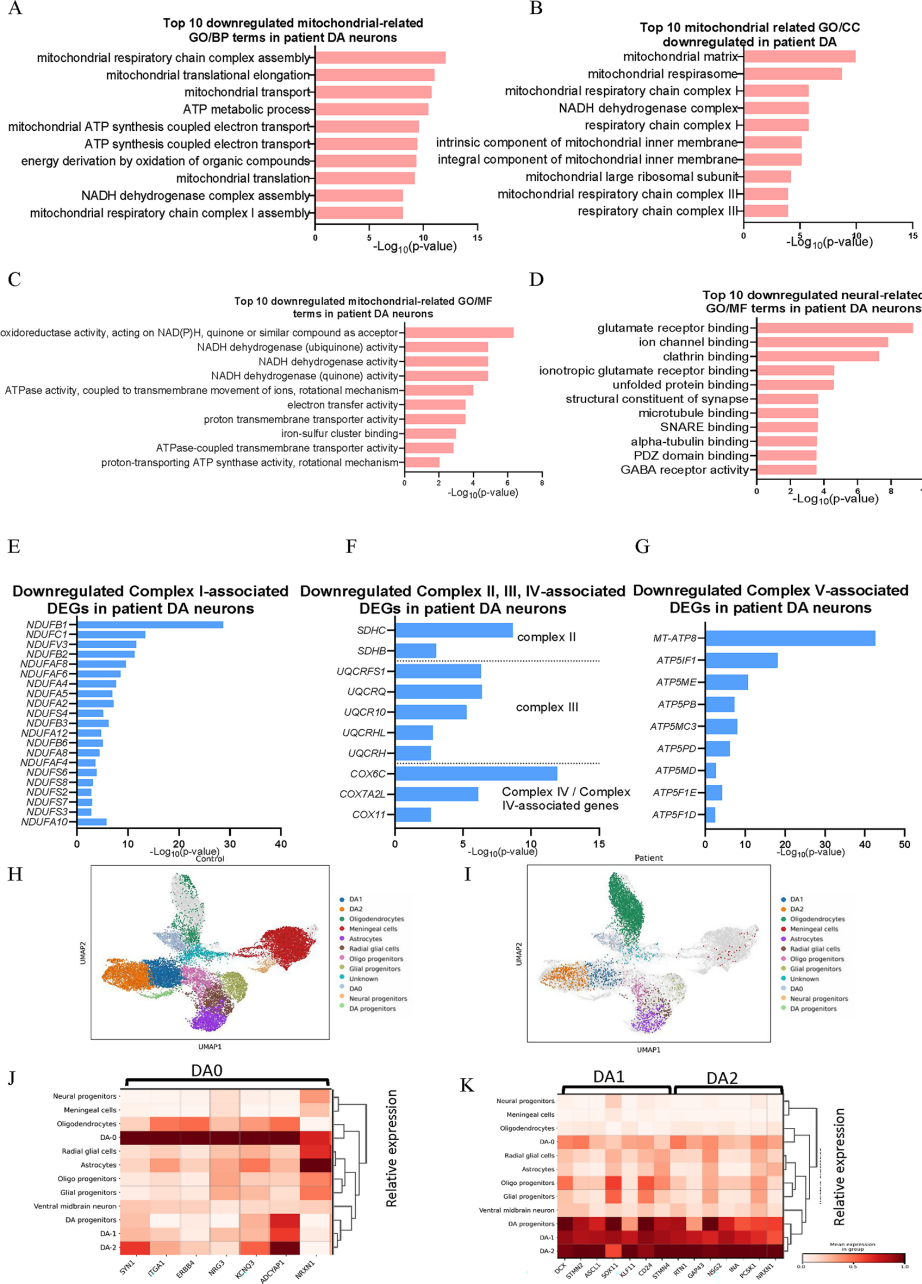
scRNA-seq analysis for GO and KEGG pathway enrichment in MOs derived from POLG iPSCs versus controls. (**A**–**C**) GO enrichment analyses (BP, CC, MF) of downregulated mitochondrial-associated DEGs in DA neurons from POLG MOs. (**D**) Top neural-associated GO/MF terms downregulated in POLG DA neurons. (**E**) Downregulated Complex I-associated DEGs in patient DA neurons. (**F**) Downregulated Complex II, III, IV-associated DEGs in patient DA neurons. (**G**) Downregulated Complex V-associated DEGs in patient DA neurons. (**H**–**I**) UMAP visualization of annotated cell populations in control and POLG MOs. (**J, K**) Cluster-specific differential expression patterns across identified DA0, DA1, DA2 neuronal subtypes. scRNA-seq analysis was performed on pooled cells derived from three independent organoid differentiations per condition

Mitochondrial related GO enrichment analysis of genes downregulated in DA neurons from POLG MOs further revealed significant reductions in oxidoreductase activity acting on NAD(P)H and quinone substrates, NADH dehydrogenase activity, ATPase activity, and iron–sulfur cluster binding (Fig. 3A–C; Table S3–S5), indicating compromised electron transport and mitochondrial enzymatic function.

In parallel, neuronal molecular functions were suppressed, including glutamate receptor binding, GABA receptor binding, synaptic membrane components, and microtubule-associated binding (Fig. 3D; Table S6).

Complex I (NADH dehydrogenase) displayed broad suppression of multiple structural and catalytic subunits, including *NDUFS2*, *NDUFS3*, *NDUFS4*, *NDUFS6*, *NDUFS*7, *NDUFS8*, *NDUFA2*, *NDUFA4*, *NDUFA5*, *NDUFA8*, *NDUFA10*, *NDUFA12*, *NDUFA13*, *NDUFB1*, *NDUFB2*, *NDUFB3*, *NDUFB6*, *NDUFC1*, *NDUFV3*, *NDUFAF4*, *NDUFAF6*, and *NDUFAF8* (Fig. 3E, Table S7). Complex II was also reduced, as evidenced by decreased expression of *SDHB *and *SDHC*. Complex III genes, including *UQCRH*, *UQCRHL*, *UQCRFS1*, *UQCRQ*, and *UQCR10*, were downregulated. Likewise, Complex IV subunits, including *COX7A2L*, *COX6C*, and *COX11*, were suppressed (Fig. 3F, Table S8). Complex V (ATP synthase) exhibited extensive downregulation of both F1 and Fo subunits, including *ATP5F1A*, *ATP5F1D*, *ATP5PD*, *ATP5MC1–3*, ATP5PE, , *ATP5IF1*, and *MT-ATP8*, together with *ATP6V1A*, *ATP6V1D*, and *ATP6V1H* (Fig. 3G, Table S9).

KEGG pathway analysis of upregulated DEGs revealed enrichment of stress- and remodeling-related pathways, including Apoptosis, Mitophagy (animal), ErbB signaling, Wnt signaling, Cell cycle, and Regulation of actin cytoskeleton (Fig. S10), consistent with activation of cellular stress and compensatory programs.

Collectively, these findings suggest that POLG DA neurons exhibit transcriptional changes associated with mitochondrial bioenergetic and neuronal programs, with reduced expression of respiratory chain and synaptic genes and increased expression of stress-, mitophagy-, and compensatory response pathways.

### Selective transcriptional vulnerability of DA2 neurons in POLG organoids

**Fig. 4:**
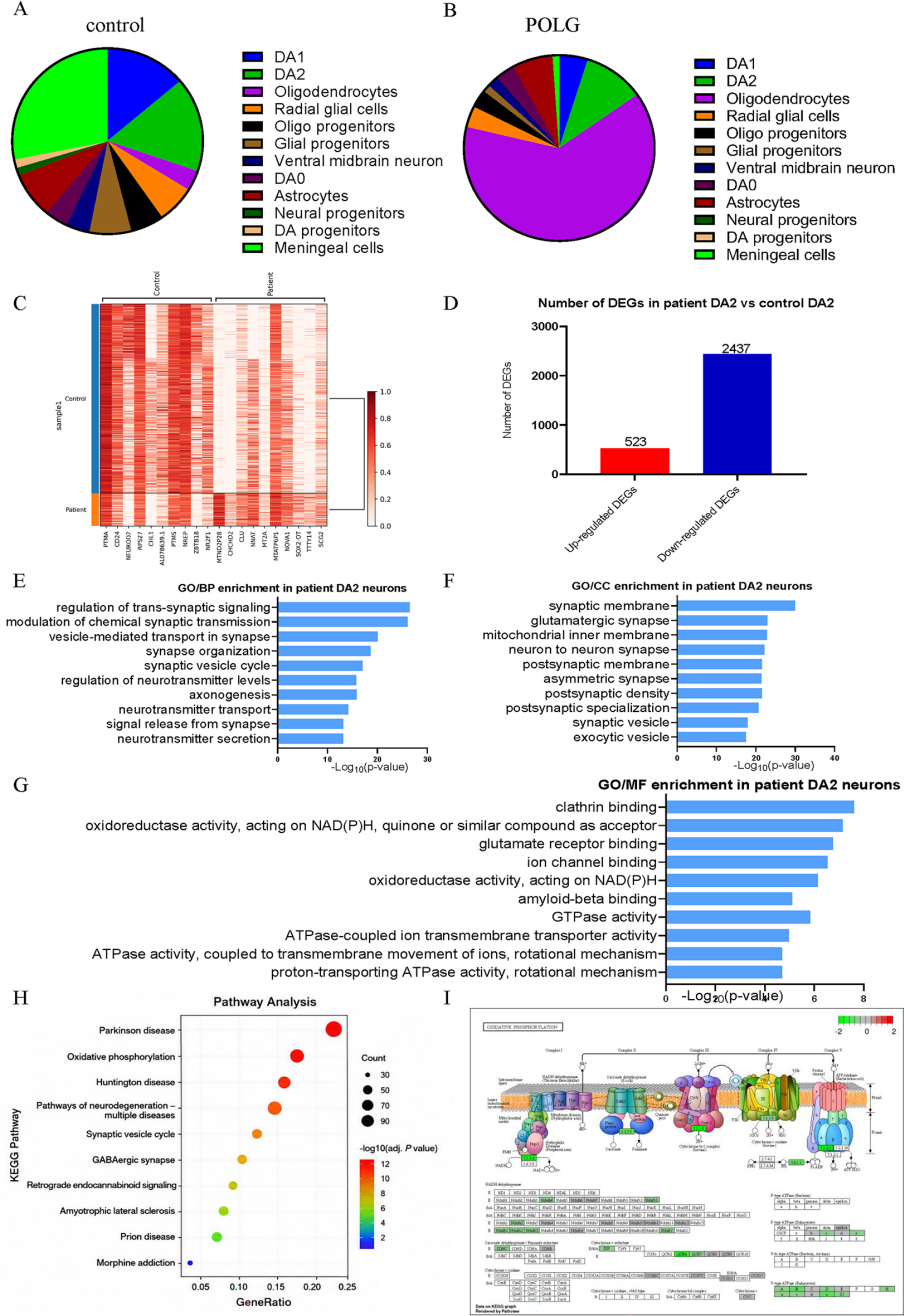
scRNA-seq analysis of DA2 neurons in POLG patient-derived MOs. (**A**–**B**) Pie charts showing distribution of major cell populations in control (A) and POLG (B) MOs. (**C**) Heatmap of differentially expressed genes in DA2 neurons. (**D**) Quantification of upregulated and downregulated DEGs in POLG DA2 neurons. (**E**–**G**) Top 10 enriched downregulated GO terms (BP, CC, MF) in POLG DA2 neurons. (**H**) KEGG pathway enrichment analysis of downregulated DEGs in POLG DA2 neurons. The x-axis represents the GeneRatio, bubble size indicates the number of genes enriched in each pathway (Count), and bubble color represents the significance level as −log10(P value). (**I**) Schematic representation of suppressed oxidative phosphorylation genes in POLG DA2 neurons. All scRNA-seq analyses were performed on pooled cells from three independent organoid differentiations per condition

To further resolve dopaminergic heterogeneity, DA neurons were subdivided into three transcriptionally distinct subpopulations (DA0, DA1, and DA2) within the dopaminergic lineage cluster based on unsupervised Leiden clustering of the integrated scRNA-seq dataset (Fig. 3H–I). Subtype identities were defined by discrete transcriptional programs reflecting progressive dopaminergic maturation and synaptic engagement. DA0 neurons displayed relatively low expression of synaptic transmission and maturation-associated genes, including *SYN1*,* NRXN1*,* ERBB4*, and *NRG3*, together with *ITGA1* and *ADCYAP1*, consistent with a transitional state characterized by early neurite outgrowth and emerging excitability (Fig. 3J). DA1 neurons were marked by increased expression of neuronal differentiation and cytoskeletal remodeling genes, including *DCX*,* STMN2*,* STMN4*,* GAP43*,* RTN1*, and *NSG2*, indicative of structural and morphological maturation. In contrast, DA2 neurons exhibited sustained expression of synaptic integration and network connectivity genes such as *GAP43*,* INA*,* PCSK1*, and *NRXN1*, representing the most transcriptionally mature and functionally engaged dopaminergic population within the dataset (Fig. 3K). Quantitative analysis revealed significant reductions in DA1 neurons (4.88% vs. 13.83%), DA2 neurons (10.36% vs. 16.40%), and VMNs (1.97% vs. 4.00%) in POLG organoids compared with controls (Fig. 4A, B). In contrast, DA0 neurons and DA progenitors showed only minor changes (Fig. 4A, B), indicating a maturation-dependent vulnerability in POLG organoids, whereby structurally and functionally differentiated dopaminergic populations are preferentially depleted. Differential expression analysis of DA2 neurons identified 2,437 downregulated and 523 upregulated genes in POLG organoids relative to controls (Fig. 4C, D), highlighting a profound transcriptional imbalance characterized predominantly by suppression of genes associated with mitochondrial bioenergetics, synaptic transmission, and neuronal structural integrity. In contrast, enrichment analyses of DA1 and DA0 neurons revealed no major alterations in mitochondrial- or neuronal-related pathways (data not shown).

GO enrichment of downregulated genes in DA2 neurons revealed strong suppression of synaptic processes, including regulation of trans-synaptic signaling, synaptic vesicle cycle, vesicle-mediated transport, axonogenesis, and neurotransmitter secretion (Fig. 4E; Table S10). Cellular component analysis identified enrichment in synaptic membrane, postsynaptic density, glutamatergic synapse, and mitochondrial inner membrane categories (Fig. 4F; Table S11). Molecular function analysis demonstrated reductions in glutamate receptor binding, ion channel binding, clathrin binding, oxidoreductase activity acting on NAD(P)H substrates, and ATPase-coupled ion transport (Fig. 4G; Table S12). KEGG pathway analysis confirmed enrichment of downregulated genes in Parkinson’s disease, Huntington’s disease, Amyotrophic lateral sclerosis, oxidative phosphorylation, GABAergic synapse, and synaptic vesicle cycle pathways (Fig. 4H; Table S13). Notably, oxidative phosphorylation emerged as a central suppressed pathway (Fig. 4I), with downregulated genes clustering within mitochondrial respiratory chain complexes. Representative components included Complex I subunits (e.g., *NDUFS2*,* NDUFS4*,* NDUFB1*), Complex III (*UQCRFS1*), Complex IV (*COX6C*), and ATP synthase subunits (*ATP5F1D*,* ATP5PD*).

Together, these findings identify DA2 neurons as a selectively vulnerable dopaminergic subtype in POLG organoids, characterized by coordinated suppression of mitochondrial respiratory chain genes and synaptic signaling networks.

### POLG DA2 neurons exhibit coordinated suppression of mitochondrial translation, respiratory chain assembly, and synaptic programs

To further define the molecular programs disrupted in terminally differentiated DA neurons, gene ontology enrichment analysis was performed on downregulated genes in DA2 cells from POLG organoids.

**Fig. 5:**
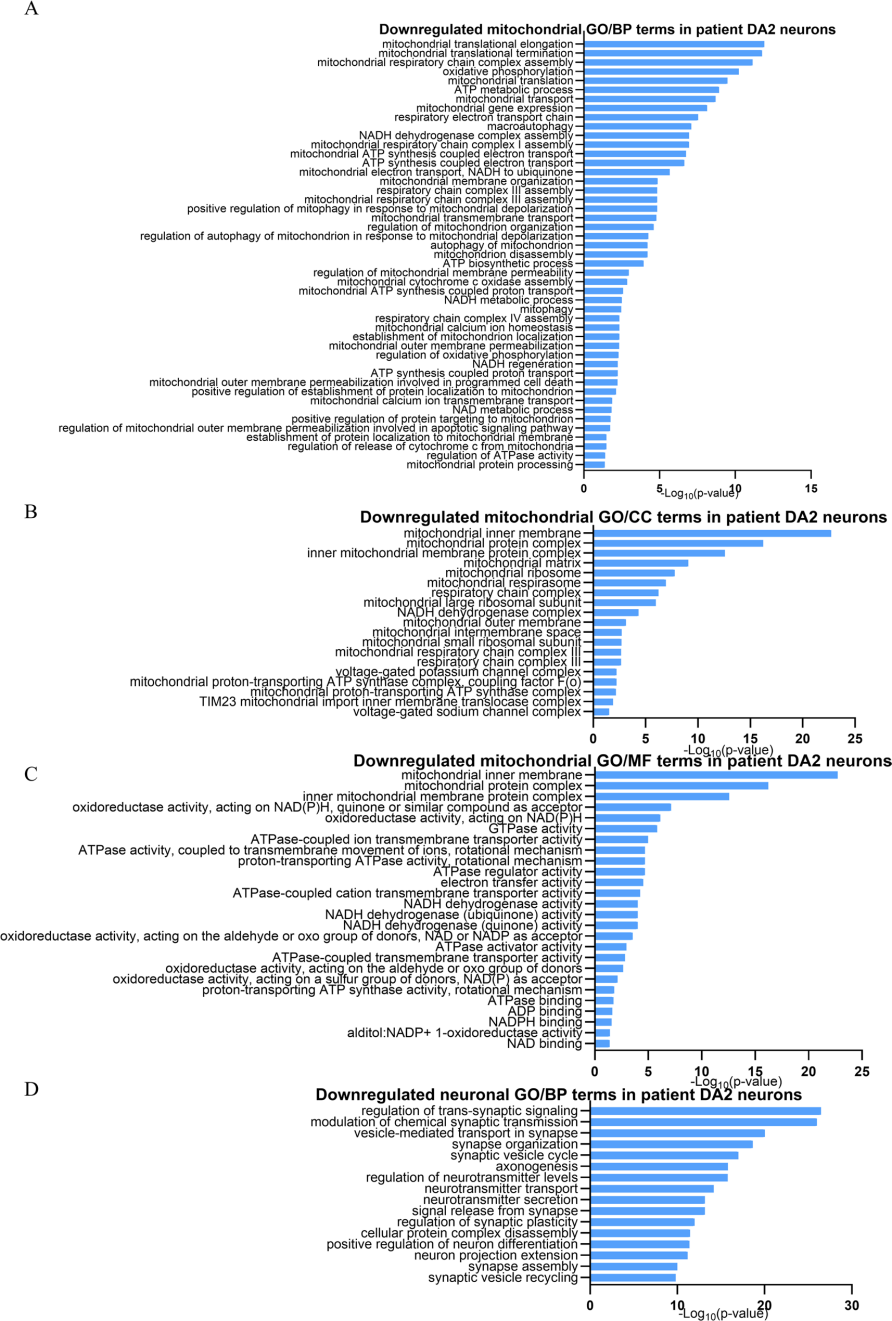
Analysis of downregulated mitochondrial and neuronal pathways in DA2 neurons of POLG patient-derived MOs. (**A**–**C**) Downregulated mitochondrial-associated GO terms (BP, CC, MF) in POLG DA2 neurons. (**D**) Downregulated neuronal-associated GO terms in POLG DA2 neurons. scRNA-seq analyses were performed on pooled cells from three independent organoid differentiations per condition

Biological process (GO/BP) analysis revealed enrichment of mitochondrial-related pathways (Fig. 5A), including mitochondrial translation elongation and termination, mitochondrial respiratory chain complex assembly, oxidative phosphorylation, ATP metabolic process, and mitochondrial electron transport. In addition, GO terms related to NADH-dependent respiration, ATP synthesis–coupled electron transport, and mitochondrial membrane organization were also significantly enriched among downregulated genes, indicating coordinated transcriptional alteration of mitochondrial functional modules.

Consistent with these findings, cellular component (GO/CC) analysis demonstrated marked depletion of structural mitochondrial compartments (Fig. 5B; Table S15), including mitochondrial inner membrane, mitochondrial protein complexes, mitochondrial matrix, respiratory chain complex, and mitochondrial respirasome. Notably, ATP synthase complexes and proton-transporting ATP synthase assemblies were significantly enriched among downregulated components, suggesting destabilization of ATP-generating machinery at the organelle level.

At the functional annotation level, molecular function (GO/MF) analysis revealed enrichment of terms associated with mitochondrial enzymatic processes (Fig. 5C; Table S16), including oxidoreductase activity acting on NAD(P)H substrates, NADH dehydrogenase activity, electron carrier activity, ATP synthase activity, and proton-transporting ATPase activity. Together, these changes suggest coordinated transcriptional modulation of mitochondrial electron transport and ATP-related processes in POLG DA2 neurons. Importantly, mitochondrial dysfunction was accompanied by widespread suppression of neuronal programs (Fig. 5D; Table S17). Downregulated biological processes included regulation of trans-synaptic signaling, modulation of chemical synaptic transmission, synaptic vesicle cycle, axonogenesis, neurotransmitter transport and secretion, synaptic plasticity, and neuron projection assembly. Together, these results demonstrate coordinated transcriptional alterations in bioenergetic and synaptic networks in DA2 neurons, identifying terminally differentiated dopaminergic cells as the most vulnerable population in POLG organoids.

Together, these data demonstrate that POLG DA2 neurons undergo coordinated suppression of mitochondrial translation and oxidative phosphorylation pathways alongside synaptic transmission networks, supporting a coupled mitochondrial–neuronal dysfunction at the transcriptional level.

### Transcriptomic changes in VMN-annotated populations in POLG organoids

**Fig. 6:**
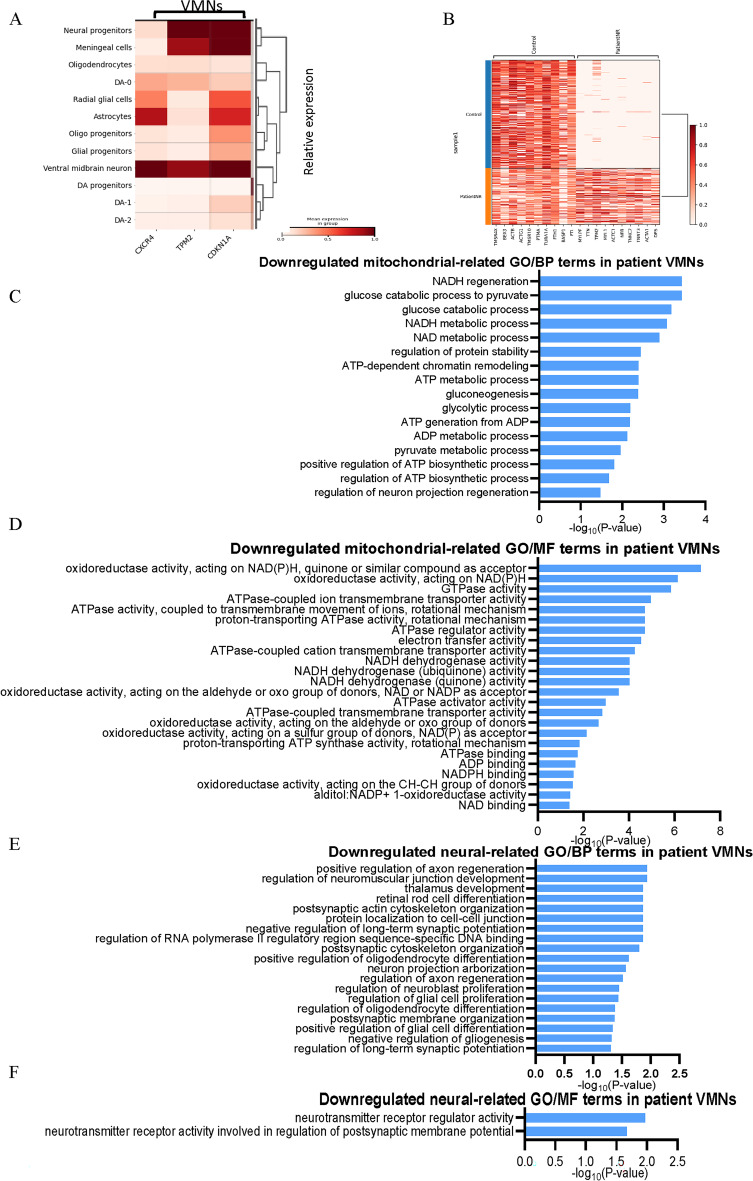
Characterization of mitochondrial and neuronal pathway alterations in VMNs of POLG patient-derived MOs. (**A**) Canonical marker expression of VMNs across annotated cell populations. (**B**) Heatmap showing DEGs in VMNs from POLG versus control MOs. (**C**–**F**) Downregulated mitochondrial- and neural-associated GO terms (BP, MF) in POLG VMNs. scRNA-seq analyses were performed on pooled cells from three independent organoid differentiations per condition

To further evaluate ventral midbrain lineage identity, canonical marker expression was examined across annotated cell populations (Fig. 6A). VMNs exhibited robust enrichment of *CXCR4*, *TPM2*, and *CDKN1A*, confirming region-specific specification and post-mitotic neuronal identity. *CXCR4* expression was particularly pronounced in VMNs, consistent with its role in neuronal positioning and ventral patterning. *TPM2* enrichment reflects cytoskeletal organization within differentiated neurons, while *CDKN1A* expression indicates cell cycle exit and terminal differentiation. In contrast, these markers were expressed at lower levels in DA1 and DA2 neuron subpopulations, and only modestly in progenitor populations, supporting the transcriptional distinction between VMNs and dopaminergic subclusters. The gene expression profiles of VMNs in POLG organoids revealed substantial transcriptional suppression compared to control cells (Fig. 6B).

**Fig. 7:**
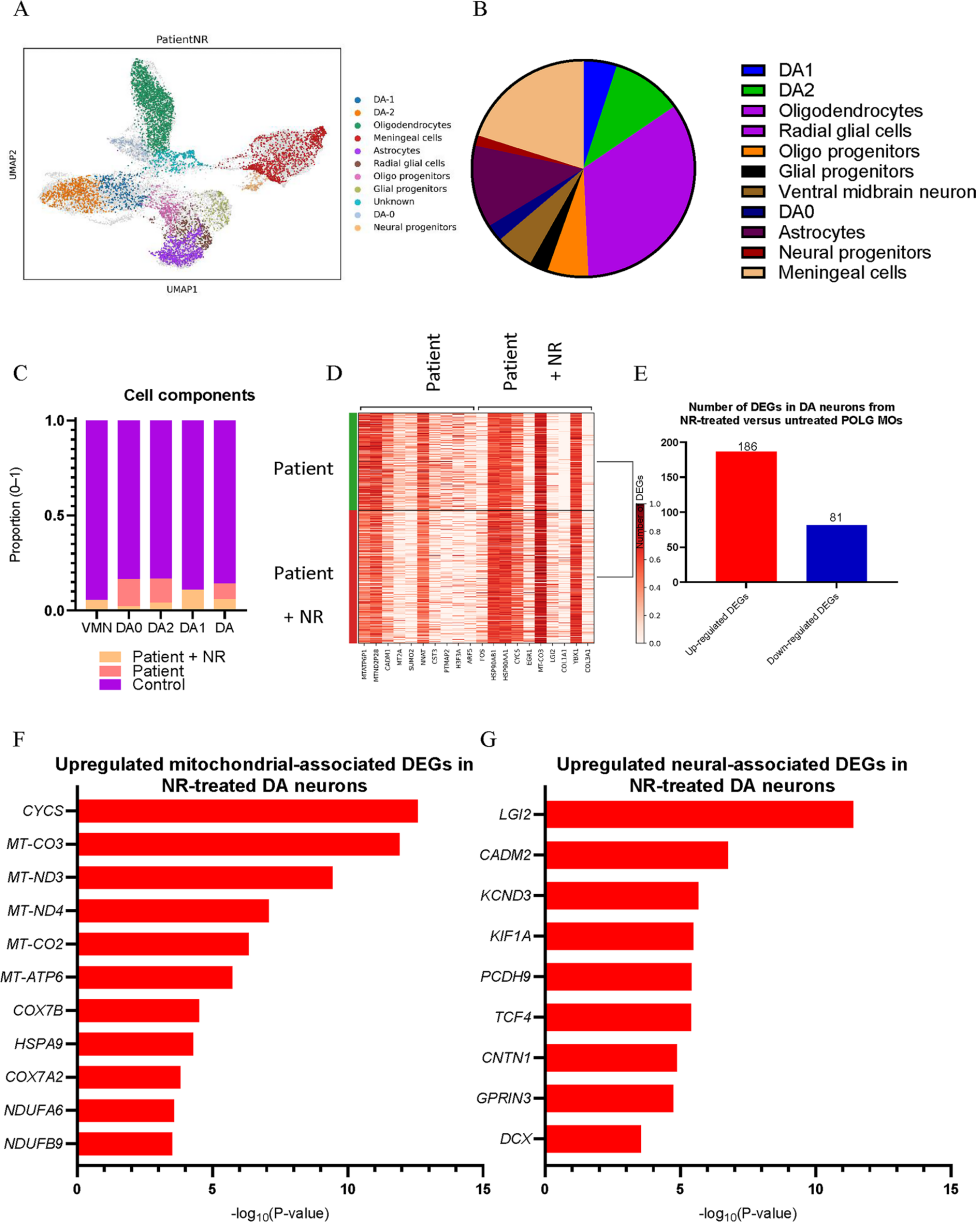
scRNA-seq analysis in DA neurons of POLG patient MOs treated with NR. (**A**–**C**) UMAP visualization and cell proportion analysis of POLG MOs with NR treatment. The values in C shown within each bar represent the normalized cell proportion (range 0–1), where 1.0 corresponds to 100% of cells within each cluster. (**D**–**E**) Differential expression analysis in DA neurons following NR treatment, including heatmaps (**D**), DEG quantification (**E**). (**F**) Upregulated mitochondrial-associated DEGs in NR-treated DA neurons. (**G**) Upregulated neural-associated DEGs in NR-treated DA neurons. scRNA-seq analyses were performed on pooled cells from three independent organoid differentiations per condition

**Fig. 8:**
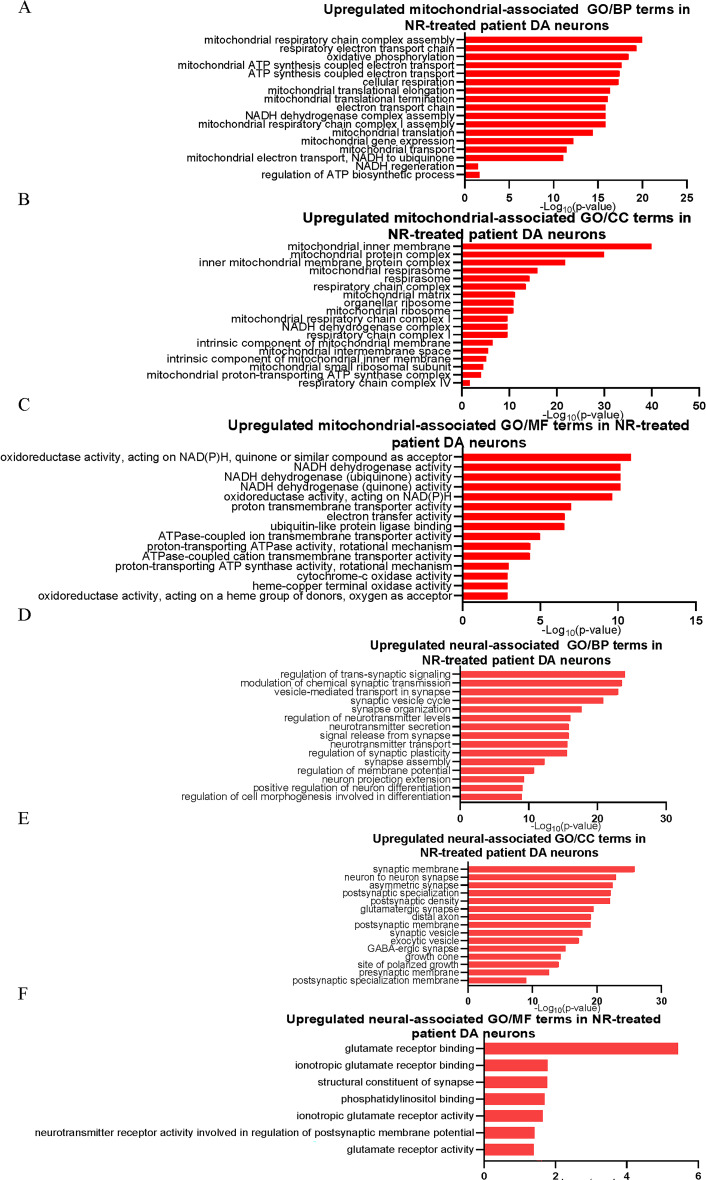
Mitochondrial and neuronal GO enrichment analysis in DA neurons from POLG patient-derived MOs following NR treatment. (**A**–**C**) Upregulated mitochondrial-associated GO terms (BP, CC, MF) in DA neurons following NR treatment. (**D**–**F**) Upregulated neuronal and synaptic-associated GO terms in DA neurons after NR treatment. scRNA-seq analysis was performed on pooled cells derived from three independent organoid differentiations per condition

**Fig. 9:**
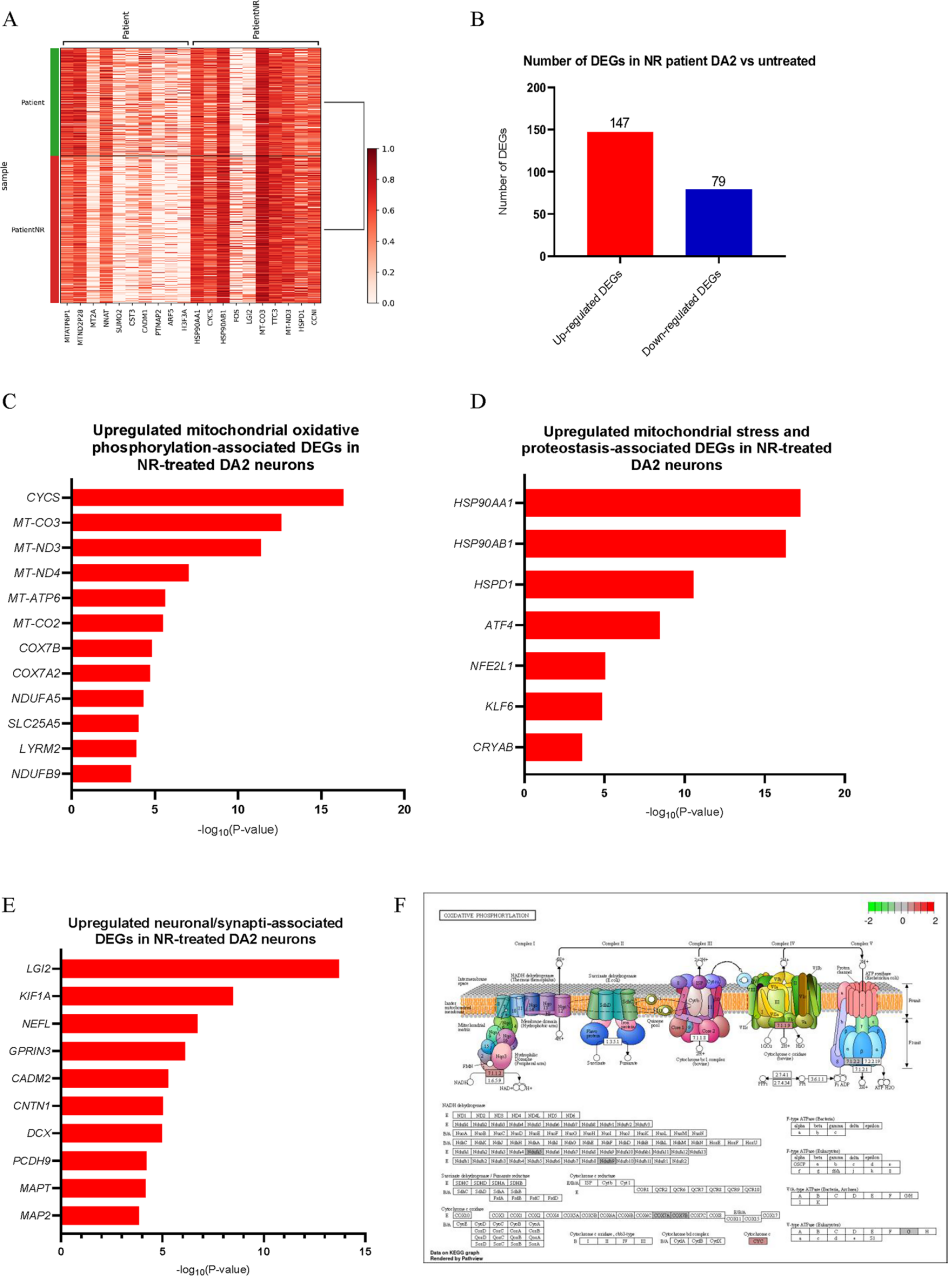
Transcriptomic profiling and pathway analysis of NR-treated DA2 neurons. (**A**) Heatmap showing the expression patterns of DEGs between patient-derived DA2 neurons and NR-treated patient DA2 neurons. (**B**) Number of significantly upregulated and downregulated DEGs identified following NR treatment. (**C**) Upregulated mitochondrial oxidative phosphorylation-associated DEGs in NR-treated DA2 neurons. (**D**) Upregulated mitochondrial stress and proteostasis-associated DEGs in NR-treated DA2 neurons. (**E**) Upregulated neuronal/synaptic-associated DEGs in NR-treated DA2 neurons. (**F**) KEGG pathway mapping of upregulated DEGs onto the oxidative phosphorylation pathway. scRNA-seq analysis was performed on pooled cells derived from three independent organoid differentiations per condition

Within the mitochondrial-related BP highlighted by GO enrichment for downregulated DEGs in VMNs from POLG MOs, we observed processes related to positive regulation of axon regeneration, regulation of neuromuscular junction development, thalamus development, retinal rod cell differentiation, postsynaptic actin cytoskeleton organization, protein localization to cell-cell junctions, negative regulation of long-term synaptic potentiation, regulation of RNA polymerase II regulatory region sequence-specific DNA binding, postsynaptic cytoskeleton organization, positive regulation of oligodendrocyte differentiation, neuron projection arborization, and more (Fig. 6C, Table S18). These enrichment results should be interpreted in a descriptive rather than lineage-specific manner, as VMN populations were defined using reference-based annotation and may include transcriptionally related neuronal states rather than a strictly anatomically defined midbrain population. Accordingly, some of the observed GO terms may reflect shared developmental transcriptional programs or limitations in reference dataset resolution, rather than distinct biological processes specific to VMNs.

Regarding mitochondrial-related MF, the top GO terms included oxidoreductase activity (acting on NAD(P)H, quinone, or similar compounds as acceptors), GTPase activity, ATPase-coupled ion transmembrane transporter activity, proton-transporting ATPase activity (rotational mechanism), ATPase regulator activity, electron transfer activity, ATPase-coupled cation transmembrane transporter activity, NADH dehydrogenase activity, NADH dehydrogenase (ubiquinone) activity, NADH dehydrogenase (quinone) activity, and many others (Fig. 6D, Table S19).

Similarly, GO enrichment for downregulated DEGs within VMNs unveiled neural-related BP. These encompassed functions like positive regulation of axon regeneration, regulation of neuromuscular junction development, thalamus development, retinal rod cell differentiation, postsynaptic actin cytoskeleton organization, protein localization to cell-cell junctions, negative regulation of long-term synaptic potentiation, regulation of RNA polymerase II regulatory region sequence-specific DNA binding, postsynaptic cytoskeleton organization, positive regulation of oligodendrocyte differentiation, neuron projection arborization, regulation of neuroblast proliferation, and more (Fig. 6E, Table S20). Additionally, GO enrichment for downregulated DEGs within VMNs also revealed neural-related MF. These included functions such as neurotransmitter receptor regulator activity and neurotransmitter receptor activity involved in regulating postsynaptic membrane potential (Fig. 6F, Table S21).

Overall, this analysis sheds light on the gene expression alterations observed in VMNs within POLG MOs, as well as the molecular changes associated with these neurodegenerative conditions.

### NR treatment partially improves dopaminergic subtype representation and mitochondrial–synaptic transcriptional programs

To evaluate the impact of NR on POLG organoids, single-cell transcriptomic profiling was performed following chronic NR treatment. UMAP visualization demonstrated preservation of major cell populations, including DA0, DA1, DA2 neurons, VMNs, astrocytes, oligodendrocytes, and DA progenitor populations (Fig. 7A, B). Quantitative analysis revealed an increase in DA neuron’s subtypes following NR treatment. The proportion of DA2 neurons increased from 12.33% to 16.79%, while VMNs increased from 1.97% to 5.59% (Fig. 7C), indicating partial normalization of midbrain neuronal representation. Differential expression analysis in DA neurons identified 186 upregulated and 81 downregulated genes following NR treatment (Fig. 7D, E).

GO enrichment of upregulated genes demonstrated significant activation of mitochondrial respiratory chain complex assembly, respiratory electron transport chain, oxidative phosphorylation, mitochondrial ATP synthesis coupled electron transport, and energy derivation by oxidation of organic compounds (Fig. S11A). In parallel, synaptic programs including regulation of trans-synaptic signaling, synaptic vesicle cycle, vesicle-mediated transport, and synapse organization were enriched. Cellular component analysis confirmed enrichment of mitochondrial inner membrane, mitochondrial protein complex, synaptic membrane, and postsynaptic density categories (Fig. S11B). Molecular function analysis further demonstrated increased oxidoreductase activity acting on NAD(P)H substrates, NADH dehydrogenase activity, ATPase regulator activity, and ion channel binding (Fig. 7H).

NR treatment markedly altered the transcriptional profile of DA neurons, leading to significant upregulation of mitochondrial and metabolic genes (Fig. S11C). Upregulated mitochondrial-associated DEGs in NR-treated DA neurons included *CYCS*,* MT-CO3*,* MT-ND3*,* MT-ND4*,* MT-CO2*,* MT-ATP6*,* COX7B*,* HSPA9*,* COX7A2*,* NDUFA6*, and *NDUFB9* (Fig. 7F, Table S22). Upregulated neural-associated DEGs in NR-treated DA neurons included *LGI2*,* CADM2*,* KCND3*,* KIF1A*,* PCDH9*,* TCF4*,* CNTN1*,* GPRIN3*, and *DCX* (Fig. 7G, Table S23).

Together, these findings demonstrate that NR treatment changes in dopaminergic cell composition and modulation of transcriptional programs related to oxidative phosphorylation and synaptic function in DA neurons from POLG MOs.

### NR treatment is associated with partial normalization of mitochondrial- and synaptic-related transcriptional programs in POLG DA neurons

To determine whether NR treatment modulates the mitochondrial and neuronal deficits observed in POLG organoids, transcriptomic profiling was performed in DA neurons following treatment.

GO biological process analysis demonstrated upregulation of mitochondrial pathways previously suppressed in POLG DA neurons, including mitochondrial respiratory chain complex assembly, respiratory electron transport chain, oxidative phosphorylation, mitochondrial ATP synthesis coupled electron transport, cellular respiration, and mitochondrial translational elongation (Fig. 8A; Table S24).

Cellular component enrichment confirmed increased representation of mitochondrial inner membrane, mitochondrial protein complex, inner mitochondrial membrane protein complex, and mitochondrial respirasome categories (Fig. 8B; Table S25). Molecular function analysis further revealed enriched oxidoreductase activity acting on NAD(P)H substrates, NADH dehydrogenase activity, proton transmembrane transporter activity, and electron transfer activity (Fig. 8C; Table S26). In parallel, neural-related pathways were significantly upregulated. BB included regulation of trans-synaptic signaling, modulation of chemical synaptic transmission, and vesicle-mediated transport in synapses (Fig. 8D; Table S27). Cellular component analysis demonstrated enrichment of synaptic membrane, neuron-to-neuron synapse, asymmetric synapse, and postsynaptic density (Fig. 8E; Table S28), while molecular function analysis confirmed increased synaptic membrane activity and neurotransmission-related functions (Fig. 8F; Table S29).

Together, these data indicate that NR treatment partially reverses the coordinated suppression of mitochondrial respiratory and synaptic transcriptional programs observed in POLG DA neurons at a transcriptomic level.

### NR selectively reactivates translational and mitochondrial programs in metabolically vulnerable DA2 neurons

To determine whether NR exerts subtype-specific beneficial effects, we analyzed transcriptomic changes within individual dopaminergic subpopulations following treatment. Differential expression analysis revealed 147 upregulated and 79 downregulated genes in NR-treated POLG DA2 neurons compared to untreated samples (Fig. 9A, B), indicating a net transcriptional activation profile in this vulnerable dopaminergic subtype. In contrast, DA0 and DA1 populations exhibited minimal transcriptional changes and did not show significant pathway enrichment following NR treatment (data not shown), suggesting limited responsiveness in earlier or structurally intermediate dopaminergic states. GO biological process enrichment analysis of upregulated genes in NR-treated DA2 neurons revealed significant activation of protein targeting and intracellular transport pathways (Fig. S12A). Prominent categories included protein localization to endoplasmic reticulum, cotranslational protein targeting to membrane, SRP-dependent cotranslational protein targeting to membrane, and establishment of protein localization to the endoplasmic reticulum. In addition, cytoskeleton-dependent intracellular transport, transport along microtubule, and actin–myosin filament sliding was enriched.

Cellular component enrichment analysis of upregulated genes in NR-treated DA2 neurons demonstrated strong activation of ribosome-associated structures (Fig. S12B). The most significantly enriched categories included cytosolic ribosome, ribosomal subunit, ribosome, cytosolic large ribosomal subunit, and cytosolic small ribosomal subunit. Additional enrichment of cell–substrate junction and focal adhesion components suggest concurrent enhancement of structural stabilization and cytoskeletal anchoring mechanisms.

Neurons further showed enrichment of molecular function categories related to protein binding, microtubule binding, ribosomal components, and unfolded protein binding (Fig. S12C). The most significantly enriched category was structural constituent of ribosome, accompanied by enrichment of rRNA binding, 5 S rRNA binding, and translation regulator activity. Functions associated with mRNA binding and translation factor activity were also increased, indicating enhanced ribosome assembly and translational initiation capacity. In addition, enrichment of actin binding and cadherin binding suggests concurrent partial normalization of cytoskeletal organization and cell–cell interaction programs.

NR treatment in DA2 cells resulted in the upregulation of genes associated with mitochondrial oxidative phosphorylation and energy metabolism, including components of the electron transport chain and ATP production machinery (*CYCS*,* MT-CO3*,* MT-ND3*,* MT-ND4*,* MT-ATP6*,* MT-CO2*,* COX7B*,* COX7A2*,* NDUFA5*,* SLC25A5*,* LYRM2*, and *NDUFB9*) (Fig. 9C, Table S30). Increased expression of stress-response and proteostasis-related genes (*HSP90AA1*,* HSP90AB1*,* HSPD1*,* ATF4*,* NFE2L1*,* KLF6*, and *CRYAB*) was also observed (Fig. 9D, Table S31), indicating upregulation of cellular stress adaptation and protein quality-control pathways. In addition, genes involved in neuronal development, axonal organization, neuronal adhesion, and cytoskeletal structure (*LGI2*,* KIF1A*,* NEFL*,* GPRIN3*,* CADM2*,* CNTN1*,* DCX*,* PCDH9*,* MAPT*, and *MAP2*) were upregulated (Fig. 9E, Table S31), suggesting enriched neuronal maturation and structural organization following NR treatment.

To further investigate the biological relevance of these transcriptional changes, upregulated DEGs were mapped to the oxidative phosphorylation pathway (Fig. 9F). Several genes were localized to key components of the mitochondrial electron transport chain (ETC), including Complex I (NADH dehydrogenase) (*MT-ND4*, *NDUFA5*, and *NDUFB9*), Complex IV (cytochrome c oxidase) (*MT-CO3*), and Complex V (ATP synthase) (*MT-ATP6*).

 Collectively, these data indicate that NR treatment preferentially enhances translational activity and stress-adaptation programs inPOLG DA2 neurons.

### NR is associated with enhanced expression of mitochondrial and neuronal pathways in POLG DA2 neurons

**Fig. 10:**
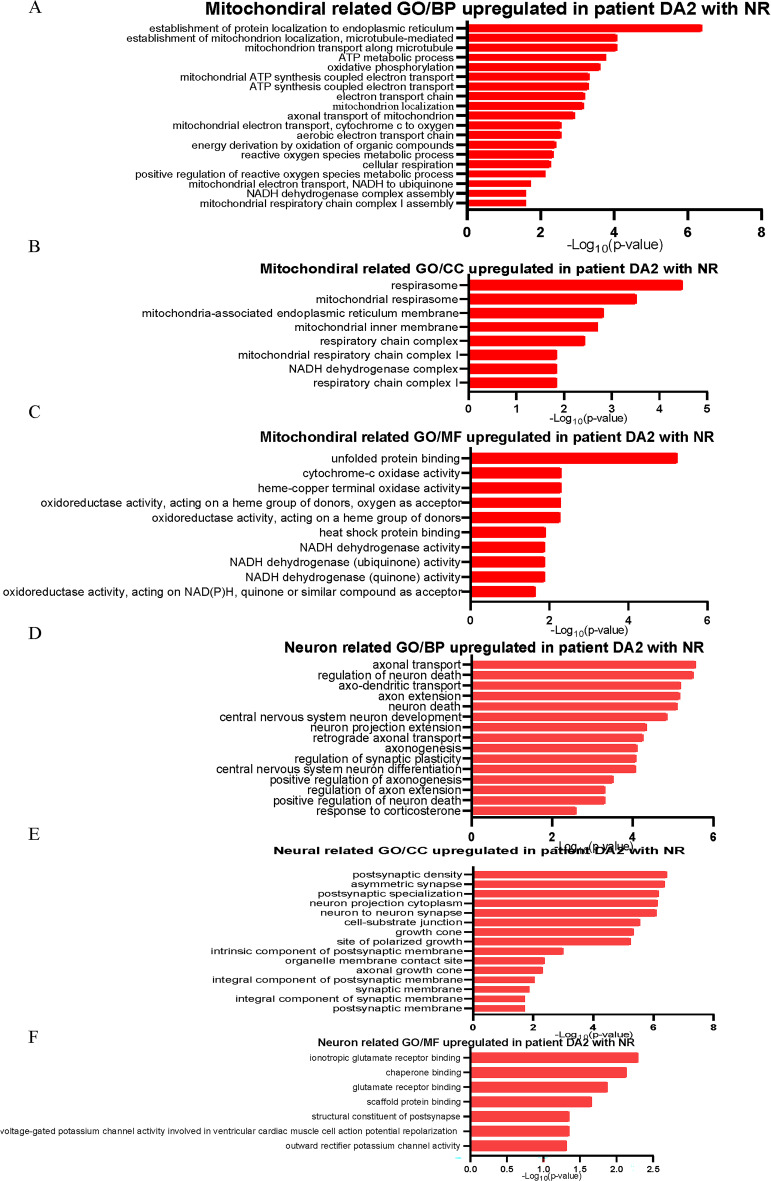
Functional enrichment analysis of DEGs following NR treatment in DA2 neurons. (**A**–**F**) GO enrichment analysis of upregulated DEGs categorized into BP, CC, and MF terms. The top enriched GO terms are shown and ranked according to enrichment score (− log10 P value). Bar lengths represent enrichment significance based on −log10(P value). scRNA-seq analysis was performed on pooled cells derived from three independent organoid differentiations per condition

GO biological process enrichment of upregulated genes demonstrated reactivation of mitochondrial pathways that were previously suppressed in POLG DA2 neurons. These included oxidative phosphorylation, mitochondrial electron transport chain, NADH dehydrogenase complex assembly, ATP metabolic process, cellular respiration, and microtubule-mediated mitochondrion transport (Fig. 10A; Table S25).

Cellular component enrichment further supported partial normalization of mitochondrial architecture, highlighting respirasome, mitochondrial inner membrane, respiratory chain complex I, NADH dehydrogenase complex, and mitochondria-associated endoplasmic reticulum membrane (Fig. 10B; Table S34). Consistently, molecular function analysis revealed enhanced NADH dehydrogenase activity, cytochrome c oxidase activity, oxidoreductase activity acting on NAD(P)H substrates, and electron transfer activity (Fig. 10C; Table S35). Representative genes encoding respiratory chain components—including Complex I (*NDUFA5*,* NDUFB9*), Complex IV (*COX7A*,* COX7B*,* MT-CO3*), and mtDNA-encoded subunits such as *MT-ND4* and *MT-ATP6*—were significantly upregulated following NR treatment (Fig. 9F, G). In parallel, neuronal transcriptional programs were also enhanced. Enriched biological processes included axonal transport, axon extension, neuron projection extension, axonogenesis, regulation of synaptic plasticity, and regulation of neuron death (Fig. 10D; Table S36). Cellular component analysis demonstrated increased representation of postsynaptic density, asymmetric synapse, synaptic membrane, glutamatergic synapse, and growth cone structures (Fig. 10E; Table S37). Molecular function enrichment further identified upregulation of ionotropic glutamate receptor binding, glutamate receptor binding, scaffold protein binding, and voltage-gated potassium channel activity (Fig. 10F; Table S38). Notably, neuronal structural genes including *DCX*, *MAP1B*, and MAP2 were elevated (Fig. 9E), consistent with enhanced neuronal maturation and cytoskeletal stabilization.

Collectively, these data demonstrate that NR treatment coordinately reactivates mitochondrial bioenergetic pathways and neuronal maturation programs in POLG DA2 neurons.

### NR treatment improves translational, mitochondrial, and synaptic programs in POLG VMNs

**Fig. 11:**
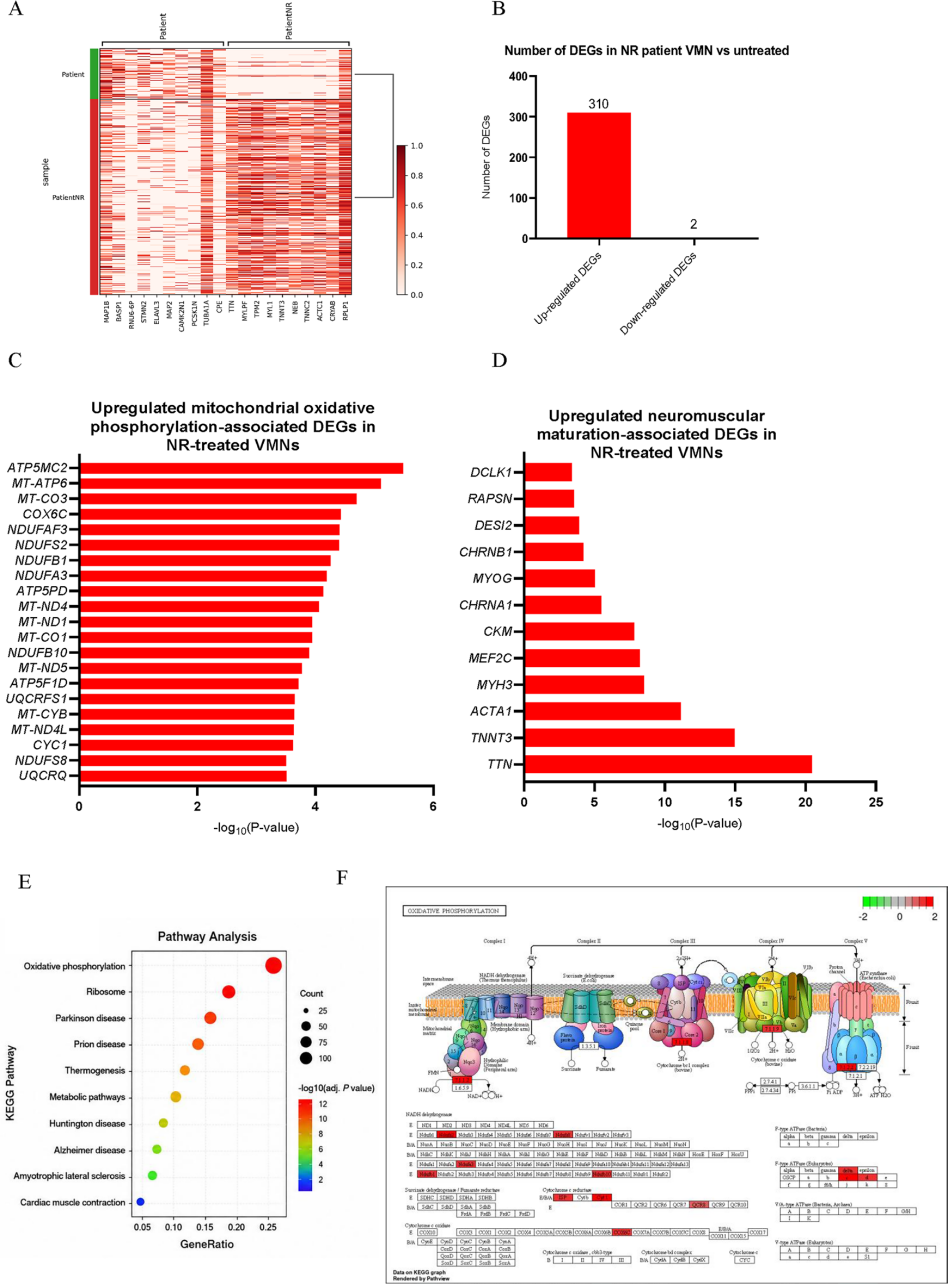
Transcriptomic and pathway analyses of NR-treated VMNs. (**A**) Heatmap of DEGs between patient-derived VMN neurons and NR-treated patient VMNs. Hierarchical clustering demonstrates distinct gene expression patterns between groups. (**B**) Number of upregulated and downregulated DEGs in VMNs identified following NR treatment. (**C**) Upregulated mitochondrial oxidative phosphorylation-associated DEGs in NR-treated VMNs. (**D**) Upregulated neuromuscular maturation-associated DEGs in NR-treated VMNs. (**E**) KEGG pathway enrichment analysis of DEGs. (**F**) Mapping of upregulated DEGs onto the oxidative phosphorylation pathway. The x-axis represents the GeneRatio, bubble size indicates the number of genes enriched in each pathway (Count), and bubble color represents the significance level as −log10(P value). scRNA-seq analysis was performed on pooled cells derived from three independent organoid differentiations per condition

Gene expression profiling of NR-treated POLG-derived VMNs demonstrated robust transcriptional activation compared to untreated samples (Fig. 11A). A total of 310 genes were upregulated, whereas only 2 genes were downregulated (Fig. 11B), indicating a strong directional beneficial effect in this neuronal population. GO biological process enrichment of upregulated genes revealed prominent activation of translational and protein-targeting pathways, including translational protein targeting to membrane, SRP-dependent translational protein targeting to membrane, protein targeting to endoplasmic reticulum, translational initiation, and nonsense-mediated decay of nuclear-transcribed mRNA (Fig. S13A). Consistently, cellular component analysis highlighted enrichment of cytosolic ribosome, large and small ribosomal subunits, and ribosomal complexes (Fig. S13B), while molecular function enrichment demonstrated increased structural constituent of ribosome, rRNA binding, translation regulator activity, and mRNA binding functions (Fig. S13C).

NR treatment in VMN cells resulted in the upregulation of genes associated with mitochondrial oxidative phosphorylation and cellular energy metabolism, including multiple components of the electron transport chain and ATP synthesis machinery (*ATP5MC2*,* MT-ATP6*,* MT-CO3*,* COX6C*,* NDUFAF3*,* NDUFS2*,* NDUFB1*,* NDUFA3*,* ATP5PD*,* MT-ND4*,* MT-ND1*,* MT-CO1*,* NDUFB10*,* MT-ND5*,* ATP5F1D*,* UQCRFS1*,* MT-CYB*,* MT-ND4L*,* CYC1*,* UQCRQ*, and *NDUFS8*) (Fig. 11C, Table S39). Increased expression of genes involved in muscle development and contractile function (*TTN*,* TNNT3*,* ACTA1*,* MYH3*,* MEF2C*,* CKM*, and *MYOG*), as well as genes associated with neuromuscular signaling and structural organization (*CHRNA1*,* CHRNB1*,* RAPSN*,* DCLK1*, and *DESI2*), was also observed following NR treatment (Fig. 11D, Table S40).

KEGG pathway analysis further identified significant enrichment of oxidative phosphorylation and neurodegeneration-related pathways, including Parkinson’s disease and prion disease (Fig. 11E). Notably, genes within the “Oxidative Phosphorylation” pathway were broadly upregulated and clustered across mitochondrial respiratory chain complexes (Fig. 11F). These included Complex I components (*NDUFB1*,* NDUFA3*,* NDUFS2*,* NDUFB10*), mtDNA-encoded subunits (*MT-ND1*,* MT-ND4*,* MT-ND5*,* MT-ND4L*,* MT-CO1*,* MT-CO3*,* MT-ATP6*), Complex III component (*MT-CYB*,* CYC1*), and ATP synthase subunits (*ATP5F1D*,* ATP5MC2*,* ATP5PD*), as well as metabolic regulators such as *PDHA1*,* PDHB*,* NDUFAF3*,* UCP2*, and *VDAC*.

**Fig. 12:**
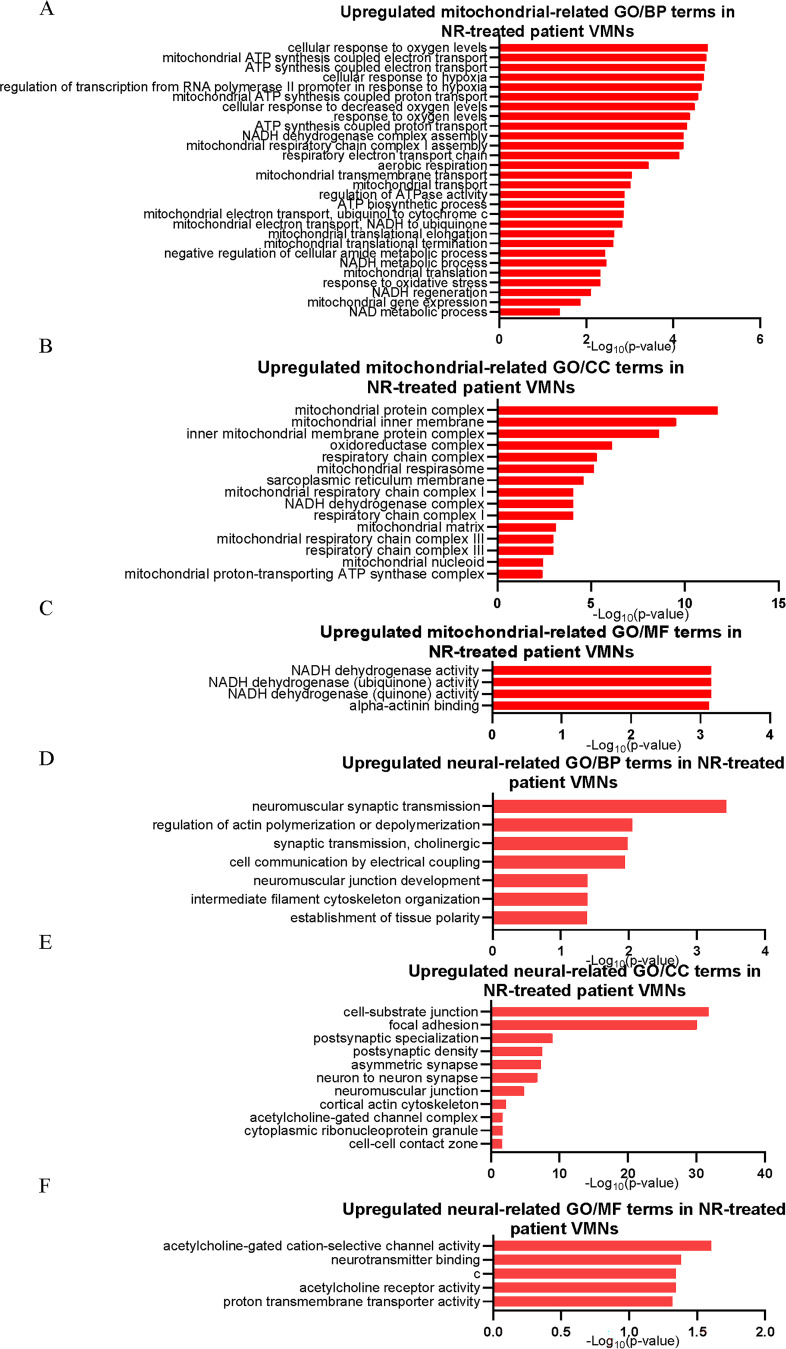
Upregulation of mitochondrial- and neuronal-related pathways in VMNs of POLG patients following NR treatment. (**A**–**C**) GO enrichment analysis showing upregulated mitochondrial-associated pathways in NR-treated VMNs, including BP, CC, and MF categories. (**D**–**F**) GO enrichment analysis showing upregulated neuronal- and synaptic-related pathways in NR-treated VMNs, including BP, CC, and MF categories. scRNA-seq analysis was performed on pooled cells derived from three independent organoid differentiations per condition

Further GO enrichment focusing on mitochondrial-related processes confirmed upregulation of mitochondrial ATP synthesis coupled electron transport, ATP synthesis coupled proton transport, aerobic respiration, NADH dehydrogenase complex assembly, mitochondrial transport, and cellular response to hypoxia (Fig. 12A, Table S41). Cellular component enrichment reinforced recovery of mitochondrial protein complex, mitochondrial inner membrane, mitochondrial respirasome, mitochondrial matrix, and mitochondrial ATP synthase complex (Fig. 12B, Table S42). Molecular function analysis further supported increased NADH dehydrogenase activity and oxidoreductase activity acting on NAD(P)H substrates (Fig. 12C, Table S43).

In parallel, neural-related pathways were also enhanced. Biological processes included neuromuscular synaptic transmission, cholinergic synaptic transmission, neuromuscular junction development, regulation of actin cytoskeleton organization, and cytoskeletal remodeling (Fig. 12D, Table S44). Cellular components such as postsynaptic specialization, neuron-to-neuron synapse, neuromuscular junction, focal adhesion, and cortical actin cytoskeleton were enriched (Fig. 12E, Table S45). Molecular function enrichment demonstrated increased acetylcholine receptor activity, acetylcholine-gated cation channel activity, and neurotransmitter binding (Fig. 12F, Table S46).

Together, these findings demonstrate that NR treatment induces a coordinated improvement of translational capacity, mitochondrial oxidative phosphorylation, and synaptic signaling programs in POLG VMNs.

## Discussion

The present study establishes a robust human MO platform derived from pluripotent stem cells and applies it to dissect the cellular and molecular consequences of *POLG* mutations. Using a dual-SMAD–based patterning strategy combined with FGF8b and SHH pathway activation via PM, we generated organoids that recapitulate key features of ventral midbrain development, including OTX2 and FOXA2 expression and limited PAX6 expression, confirming midbrain specification [35–40]. These findings are consistent with established roles of FGF-8 and SHH signaling in midbrain patterning during embryogenesis [36, 37]. Importantly, the organoids reproducibly generated mature TH⁺ DA neurons with synaptic markers, supporting their molecular and cellular relevance for modeling midbrain DA systems [41].

A central finding of this study is the selective vulnerability of DA neuron subtypes in POLG-derived organoids. *POLG* encodes the catalytic subunit of mitochondrial mtDNA polymerase gamma and is essential for mtDNA replication and maintenance [1]. Mutations in *POLG* are associated with mitochondrial dysfunction and neurodegenerative phenotypes, including dopaminergic involvement [2, 3]. In our model, POLG mutations were associated with reduced representation of DA1 and DA2 neurons and VMNs. However,the most pronounced mitochondrial and neuronal transcriptional alterations were observed in DA2 neurons and VMNs, indicating selective vulnerability ofthese neuronal populations. Single-cell analysis of DA2 neurons and VMNs identified profound downregulation of oxidative phosphorylation, NADH dehydrogenase activity, ATP synthesis, and mitochondrial respiratory chain complex assembly pathways. Given the high metabolic demands of DA neurons [42], impairment of NADH-dependent respiration may create transcriptional alterations indicative of bioenergetic stress that predisposes these neurons to degeneration, consistent with mechanisms implicated in Parkinson’s disease and related disorders [22, 43, 44].

 In DA2 neurons and VMNs, mitochondrial suppression in POLG organoids was tightly coupled to transcriptional alterations in synaptic pathways. Downregulation of genes involved in synaptic vesicle cycle, trans-synaptic signaling, glutamate receptor binding, ion channel activity, and synaptic structural organization suggests that mitochondrial dysfunction extends beyond energy metabolism to impact neurotransmission. Synaptic integrity is critical for neuronal survival and plasticity [45–48], and disturbances in glutamatergic and GABAergic signaling have been implicated in neurodegenerative pathology [46]. The convergence of oxidative phosphorylation deficits and synaptic gene suppression aligns with broader observations that mitochondrial dysfunction represents a shared mechanism across neurodegenerative diseases [8, 49, 50].

KEGG pathway enrichment further highlighted associations with neurodegenerative pathways, including Parkinson’s disease, Huntington’s disease, and ALS. Mitochondrial dysfunction is increasingly recognized as a core pathogenic mechanism in Parkinson’s disease [5, 51, 52], and suppression of respiratory chain complex genes—particularly those related to Complex I and Complex IV—has been linked to DA degeneration [9]. Our findings extend these concepts to POLG-associated pathology, suggesting that mtDNA instability may initiate a cascade of respiratory insufficiency and downstream synaptic impairment.

To explore therapeutic modulation of this mitochondrial–synaptic axis, we examined the effects of NR, a NAD⁺ precursor known to support mitochondrial metabolism. NR treatment partially enhanced DA subtype representation, particularly within the DA2 population, and induced upregulation of oxidative phosphorylation pathways. Enhanced expression of genes associated with NADH dehydrogenase activity, mitochondrial respiratory chain complexes, ATP synthesis coupled electron transport, and respirasome assembly suggests improved mitochondrial bioenergetic capacity. Importantly, synaptic-related transcriptional programs—including trans-synaptic signaling and synaptic membrane components—were concurrently upregulated, indicating coordinated enhancement of neuronal pathways.

Subtype-specific analyses revealed particularly transcriptional improvement effects in DA2 neurons and VMNs, populations that exhibited pronounced vulnerability under POLG conditions after NR treatment. In VMNs, NR treatment activated translational and mitochondrial respiratory programs, including genes within the oxidative phosphorylation pathway, further reinforcing the central role of NADH-dependent electron transport in neuronal stabilization. These findings are consistent with the broader concept that enhancing mitochondrial function may counteract neurodegenerative processes linked to respiratory chain insufficiency.

Collectively, our data supports a model in which *POLG* mutations disrupt mitochondrial homeostasis, leading to respiratory chain suppression, NADH-dependent bioenergetic failure, and coordinated synaptic transcriptional collapse. Increased NAD⁺ availability through NR was associated with partial reactivation of mitochondrial and neuronal transcriptional programs, highlighting mitochondrial bioenergetics as a therapeutic entry point in POLG-related neurodegeneration. While further in vivo validation is required, this human organoid system provides a mechanistic framework for understanding mitochondrial-driven dopaminergic vulnerability and offers a translational platform for metabolic intervention strategies.

To integrate these findings, we propose a mitochondria–synapse axis model underlying POLG-associated dopaminergic vulnerability (summarized in the Fig. S14). In this framework, *POLG* mutations impair mtDNA maintenance, leading to suppression of respiratory chain complexes—particularly Complex I and IV—and consequent disruption of NADH-dependent electron transport. The resulting bioenergetic insufficiency compromises oxidative phosphorylation and ATP production, which are essential for sustaining synaptic vesicle cycling, ion channel activity, and neurotransmitter release. This coordinated mitochondrial and synaptic transcriptional collapse renders high-energy-demand neuronal subtypes, especially DA2 neurons and ventral midbrain neurons, selectively vulnerable. Importantly, NAD⁺ augmentation through nicotinamide riboside treatment appears to intervene at the level of NADH oxidation, enhancing respiratory chain activity and restoring oxidative phosphorylation capacity. The reactivation of mitochondrial bioenergetics is accompanied by stabilization of synaptic transcriptional programs, suggesting functional coupling between mitochondrial respiration and synaptic integrity. Thus, our data support a mechanistic cascade in which mtDNA instability drives respiratory suppression, leading to synaptic destabilization and DA dysfunction, whereas metabolic supplementation partially improves the mitochondria–synapse axis and improves neuronal resilience.

Importantly, we acknowledge that the effects of NR observed in this study are based on transcriptomic changes and alterations in cellular composition, and that functional recovery was not directly assessed. Accordingly, these findings should be interpreted as modulation of mitochondrial and neuronal gene expression programs rather than evidence of restored mitochondrial or neuronal function. While NR treatment was associated with changes in genes related to oxidative phosphorylation, NADH dehydrogenase activity, and synaptic pathways, we did not measure NAD⁺/NADH levels or mitochondrial enzymatic activity. Therefore, a mechanistic interpretation of NR via NAD⁺ boosting cannot be established in this study. Instead, NR should be considered as a metabolic perturbation associated with partial modulation of disease-related transcriptional states in POLG-derived organoids. Future studies incorporating NAD⁺ quantification and direct bioenergetic assays will be required to determine the functional relevance of these changes. .

### Limitations

Several limitations of the present study should be considered. First, the conclusions regarding impaired mitochondrial respiration and the effects of NR are primarily based on transcriptional profiling and pathway enrichment analyses. Although coordinated changes in genes associated with oxidative phosphorylation, mitochondrial translation, and synaptic function were observed, direct functional measurements of mitochondrial activity, including oxygen consumption rate, respiratory complex activity, ATP production, and NAD^+^/NADH balance, were not performed. Therefore, the observed transcriptomic alterations should be interpreted as changes associated with mitochondrial and neuronal programs rather than definitive evidence of functional restoration or impairment.

Second, single-cell suspensions from biological replicates were pooled prior to library preparation, which reduced technical variation but precluded replicate-aware statistical modeling and may increase the risk of pseudoreplication in differential expression analyses. Future studies incorporating independent replicate-level sequencing and pseudobulk approaches would provide stronger statistical support.

Third, while midbrain organoids reproduce key aspects of human neurodevelopment and dopaminergic lineage specification, they do not fully recapitulate the complexity of the in vivo brain environment, including vascularization, immune interactions, long-range circuit connectivity, and age-associated processes. Thus, some disease-associated cellular responses observed in organoids may not fully reflect the pathophysiology of POLG disease in patients.

Fourth, NR treatment was performed using a single concentration and treatment paradigm. Although the selected dose was based on previous observations and adapted for 3D organoid culture conditions, dose–response relationships and optimal treatment windows were not systematically evaluated. Future studies examining multiple concentrations and treatment durations will be important to determine the therapeutic range and define the relationship between NR exposure and biological responses.

Finally, although NR treatment was associated with partial normalization of mitochondrial- and synaptic-related transcriptional programs, the mechanisms underlying these changes were not directly investigated. Whether these effects are mediated through altered NAD^+^ metabolism, mitochondrial quality control pathways, redox homeostasis, or other downstream signaling pathways remain to be determined.

## Conclusions

In this study, we established a human MO model to investigate the cellular and molecular consequences of *POLG* mutations in a physiologically relevant 3D context. POLG deficiency was associated with selective vulnerability of DA neuronal subtypes and widespread transcriptional alterations, including reduced expression of genes involved in oxidative phosphorylation and synaptic function.

Single-cell transcriptomic analyses revealed that mitochondrial respiratory and synaptic gene programs are concurrently altered in POLG-associated pathology, suggesting a close association between mtDNA instability and disruption of DA neuronal states. Notably, NR treatment was associated with partial modulation of mitochondrial and synaptic gene expression signatures and changes in DA subtype proportions.

Importantly, all interpretations regarding mitochondrial respiratory function are based on transcriptomic data and do not directly measure mitochondrial activity or bioenergetic function.

Together, these findings suggest that mitochondrial dysfunction-related transcriptional remodeling is linked to DA vulnerability in POLG disease and support further investigation of metabolic interventions in human neuronal models of mitochondrial disorders.

## Supplementary Information

Below is the link to the electronic supplementary material.

Supplementary Material 1

Supplementary Material 2

Supplementary Material 3

## Data Availability

The RNA sequencing read count data has been deposited in the NCBI Gene Expression Omnibus (GEO) under accession number GSE241743. All other datasets generated and analyzed during the current study are available from the corresponding author upon reasonable request.

## Abbreviations

ALS: Amyotrophic lateral sclerosis
ATP: Adenosine triphosphate
BDNF: Brain-derived neurotrophic factor
CC: Cellular component
CDM: Chemically defined medium
DA neuron: Dopaminergic neuron
DEG: Differentially expressed gene
DAT: Dopamine transporter
ESC: Embryonic stem cell
FGF8b: Fibroblast growth factor 8b
GDNF: Glial cell line-derived neurotrophic factor
GO: Gene Ontology
IBM: Department of Biomedicine
iPSC: Induced pluripotent stem cell
KEGG: Kyoto Encyclopedia of Genes and Genomes
ROIs: Regions of interest
MF: Molecular function
MO: Midbrain organoid
mtDNA: Mitochondrial DNA
NAD⁺: Nicotinamide adenine dinucleotide
NR: Nicotinamide riboside
NAC: N-acetyl-L-cysteine
PBS: Phosphate-buffered saline
PD: Parkinson’s disease
PFA: Paraformaldehyde
PM: Purmorphamine
POLG: DNA polymerase gamma catalytic subunit
RNP: Ribonucleoprotein
scRNA-seq: Single-cell RNA sequencing
SHH: Sonic hedgehog
sgRNAs: Synthetically modified single-guide RNAs
ssODN: Single-stranded oligodeoxynucleotide
SD: Standard deviation
TH: Tyrosine hydroxylase
UMAP: Uniform Manifold Approximation and Projection
UMI: Unique molecular identifier
VMN: Ventral midbrain neuron(s)
3D: Three-dimensional
