# Supplementary material for: Impaired NADH-linked mitochondrial respiration disrupts ventral midbrain neuronal programs in POLG disease
Source: J Transl Med. 2026 Jul 31;24:988. doi: 10.1186/s12967-026-08706-w (PMC13435581; doi:10.1186/s12967-026-08706-w)
Supplement: Supplementary file 1 [file 12967_2026_8706_MOESM1_ESM.pdf]

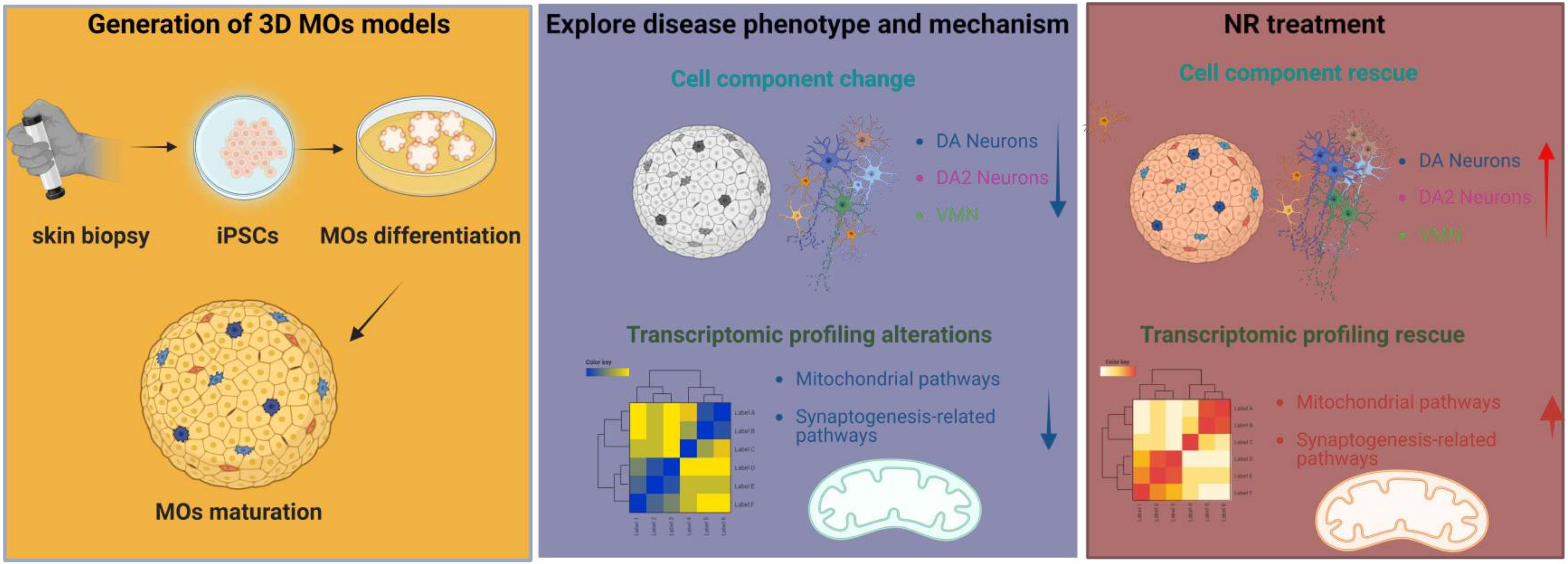

Generation of 3D MOs models
skin biopsy
iPSCs
MOs differentiation
MOs maturation
Explore disease phenotype and mechanism
Cell component change
DA Neurons
DA2 Neurons
VMN
Transcriptomic profiling alterations
Mitochondrial pathways
Synaptogenesis-related pathways
NR treatment
Cell component rescue
DA Neurons
DA2 Neurons
VMN
Transcriptomic profiling rescue
Mitochondrial pathways
Synaptogenesis-related pathways
