# Supplementary material for: Impaired NADH-linked mitochondrial respiration disrupts ventral midbrain neuronal programs in POLG disease
Source: J Transl Med. 2026 Jul 31;24:988. doi: 10.1186/s12967-026-08706-w (PMC13435581; doi:10.1186/s12967-026-08706-w)
Supplement: Supplementary file 2 [file 12967_2026_8706_MOESM2_ESM.pdf]

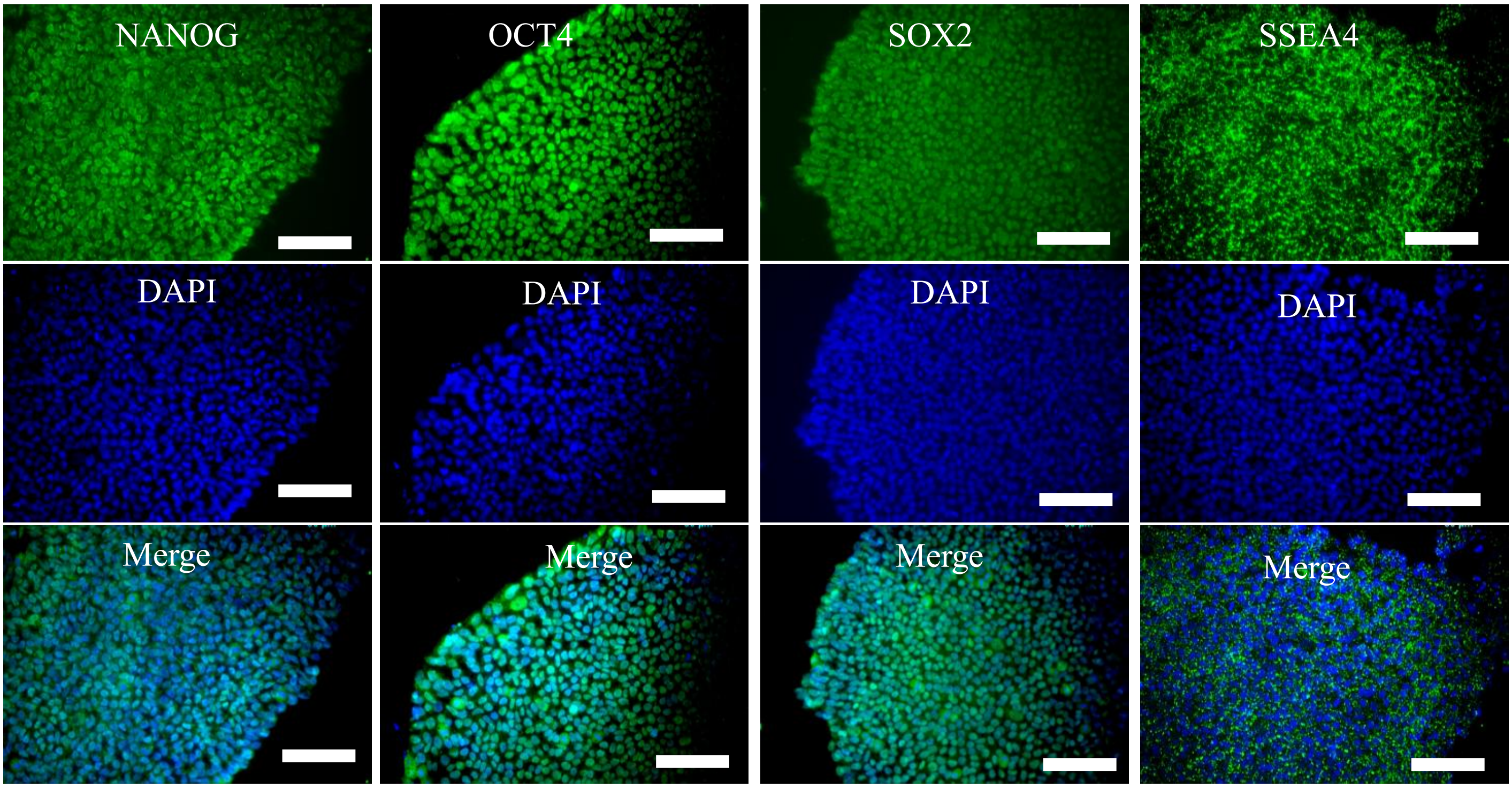

Figure S1. Representative fluorescent images of control iPSCs labeled with Nanog, OCT4, SOX2, and SSEA4. Nuclei were stained with DAPI (blue). Scale bar is 50 µm.

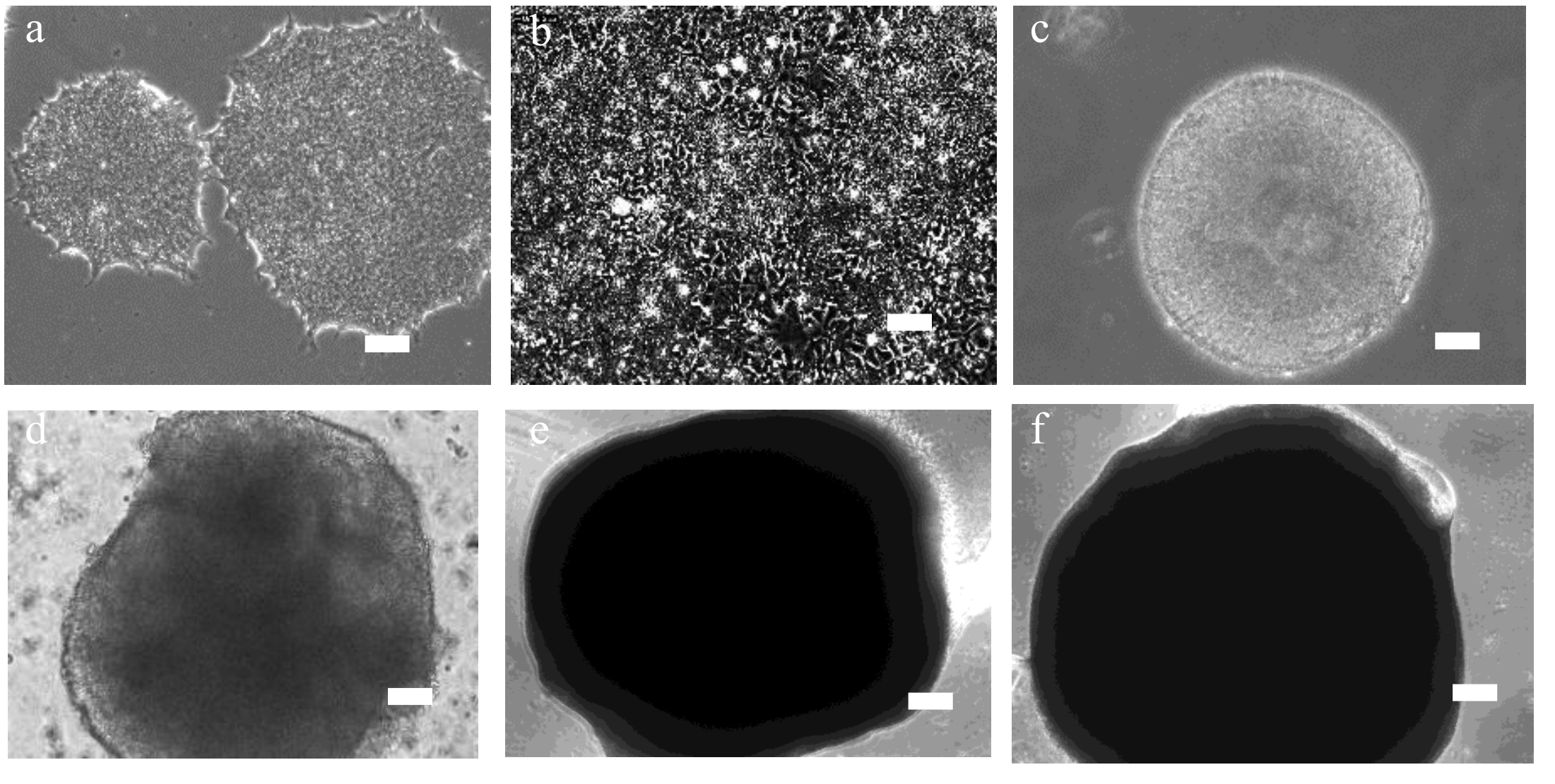

Figure S2. Representative phase-contrast images of iPSCs (a), neuroepithelium (b), DA progenitors (c) and MOs generated on days 19 (d) and maturation was achieved by day 50 in both control iPSCs (e) and ESCs (f) . Scale bar is 100 μm.

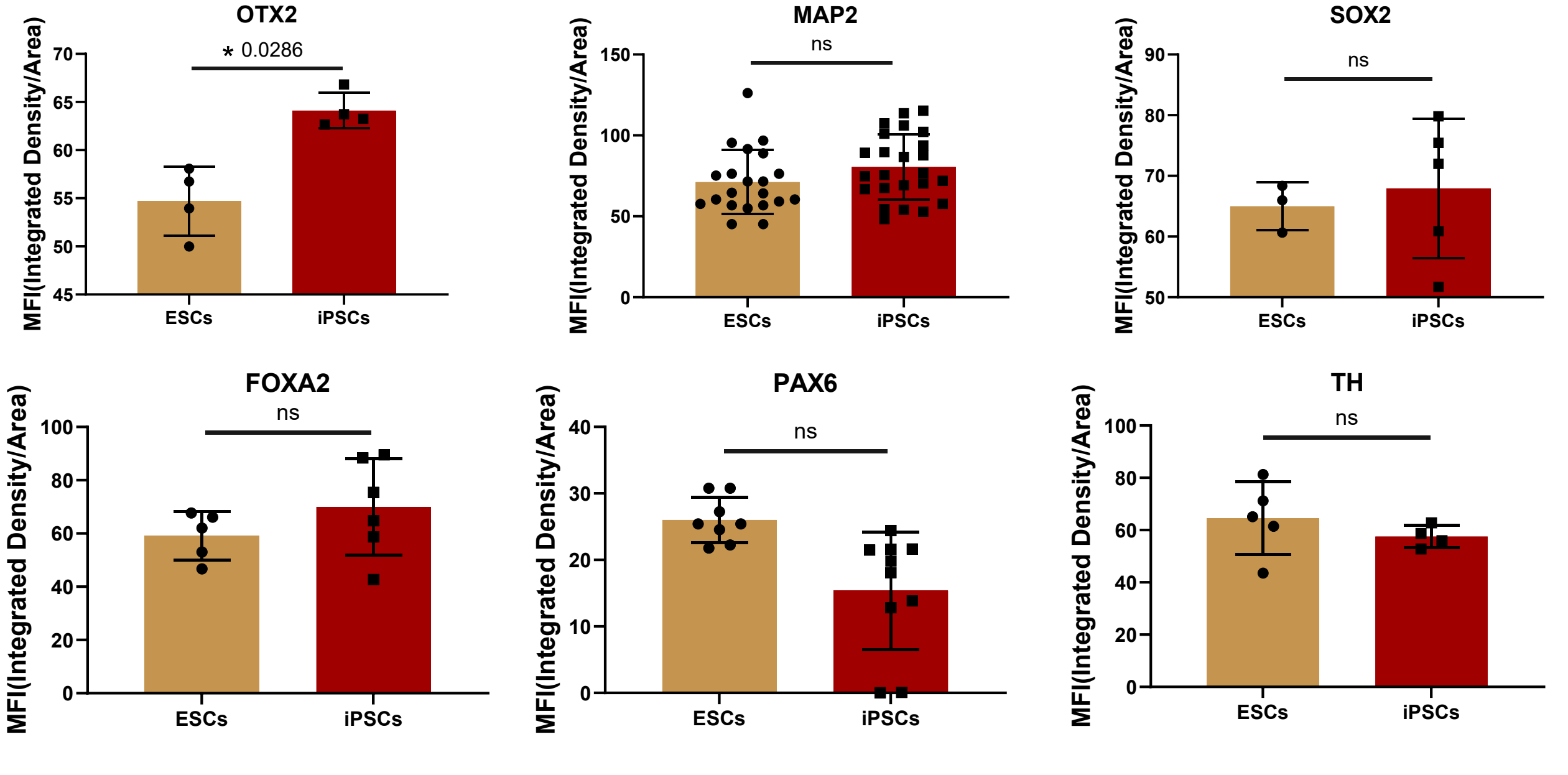

Figure S3: Quantification of immunofluorescence staining for markers for OTX2, MAP2, SOX2, FOXA2, PAX6, TH of 90-day MOs.

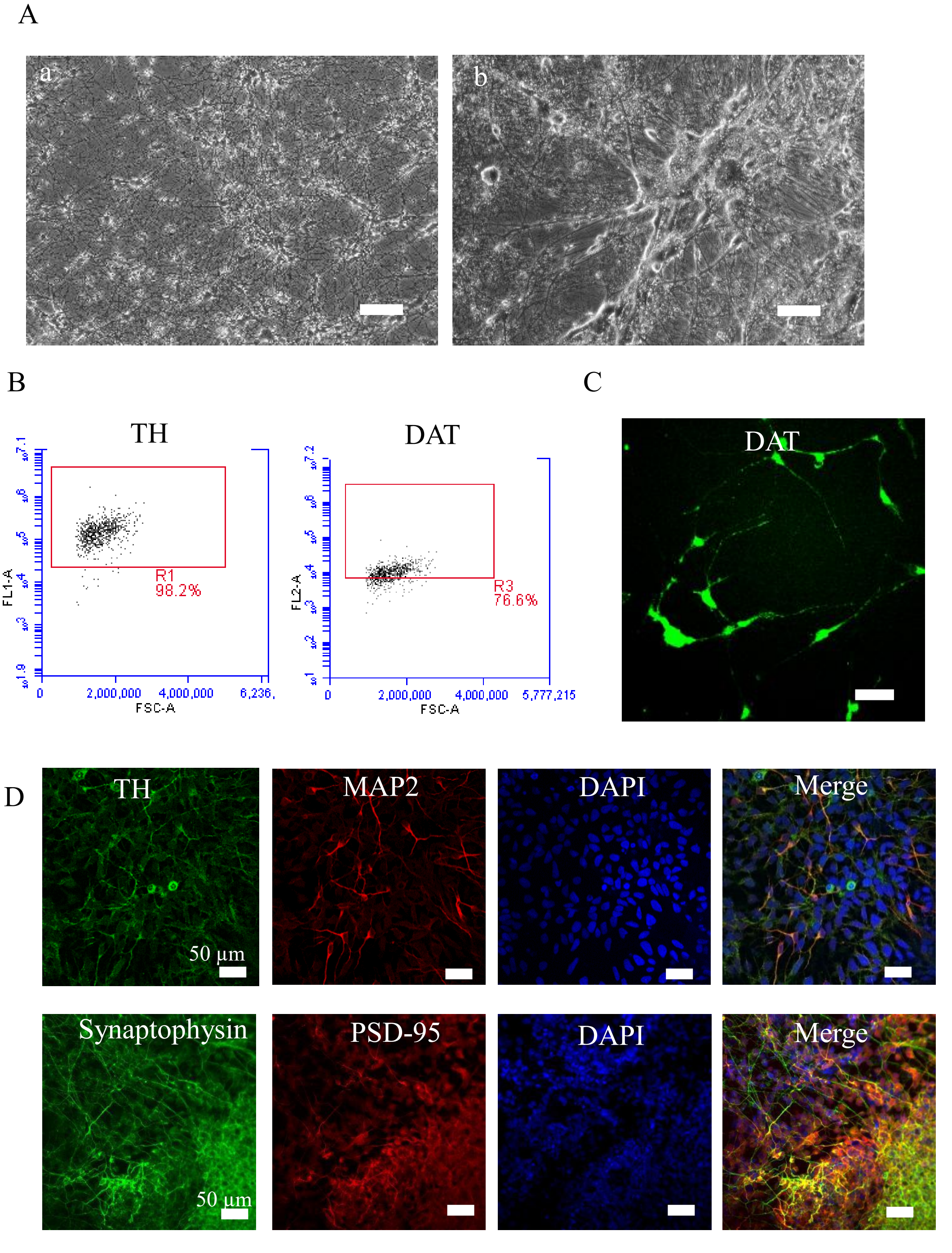

Figure S4: Differentiation and characterization of DA neurons from POLG patient and control iPSCs and generation of MO. A. Phase contrast images of mDA neurons from control iPSCs (a) and POLG iPSCs (b). Scale bar is 100 µm. B. Flow cytoetric analysis of the DA neural markers TH and DAT in MO-generated DA neurons. C. Fluorescent staining of the DA neural marker DAT in DA neurons. Scale bar is 50 µm. D. Fluorescent staining of DA neurons for the DA neural marker TH, the mature neural marker MAP2, the presynaptic marker synaptophysin, and the postsynaptic marker PSD95 in DA neurons. Nuclei were stained with DAPI (blue). Scale bar is 50 µm.

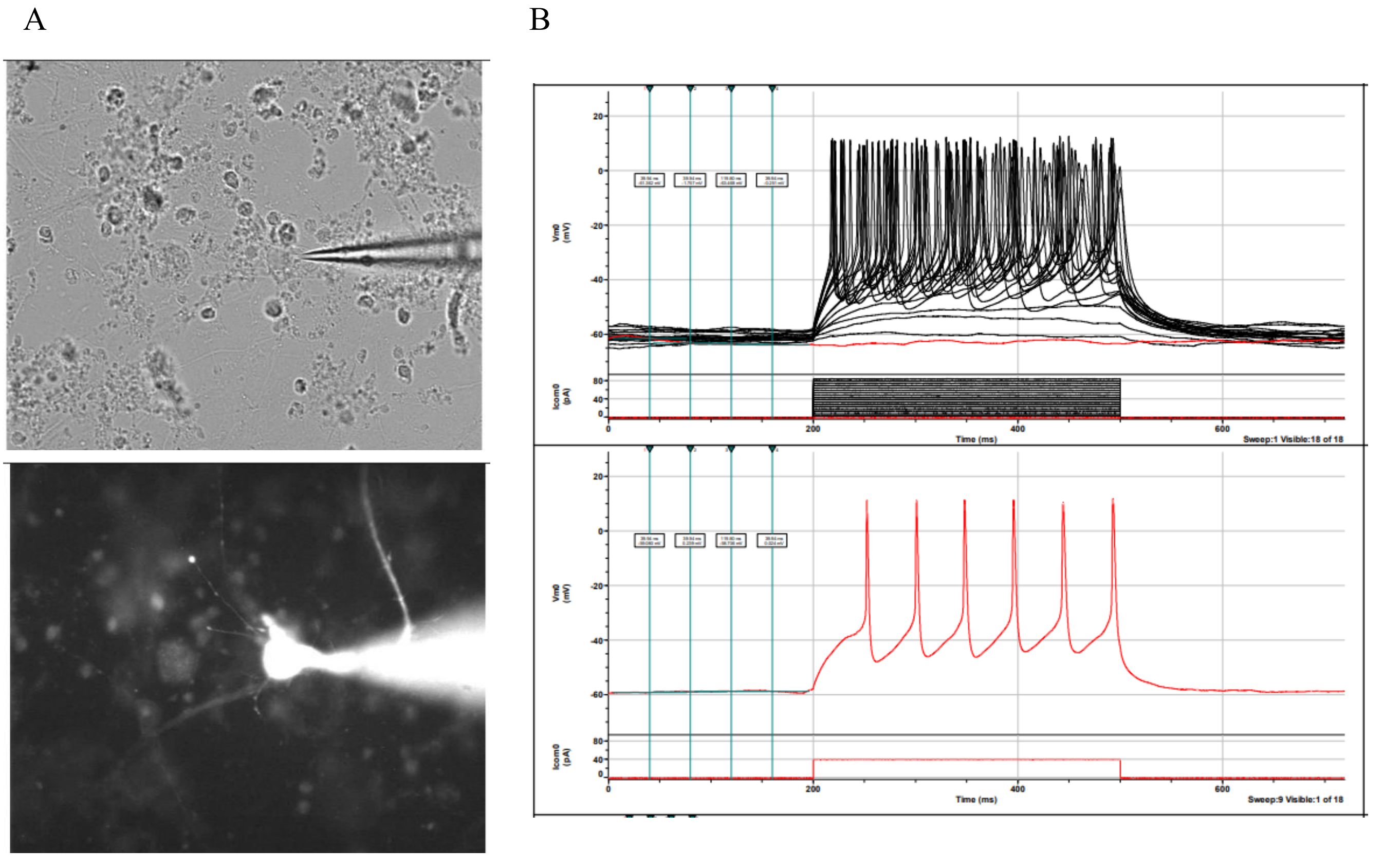

Figure S5: Electrophysiological analysis of the DA neurons generated from control iPSC-derived MOs. A. Top: Brightfield microscopy image showing the approach of a patch-clamp pipette to a DA neuron. Bottom: Fluorescence microscopy image of a DA neuron filled with fluorescent dye, delineating its structural features. B. Top: A series of electrophysiological recordings from a DA neuron demonstrating typical pace making activity, characterized by regular, autonomous firing of action potentials. Bottom: Electrophysiological recording of a single DA neuron showing the response to depolarizing current steps, characterized by a pattern of action potentials that reflects the neuron's excitability and firing properties. Scale bars: Time (ms) on the x-axis and Membrane Potential (mV) on the y-axis for the electrophysiological traces. Each trace represents an individual recording sweep from a single neuron.

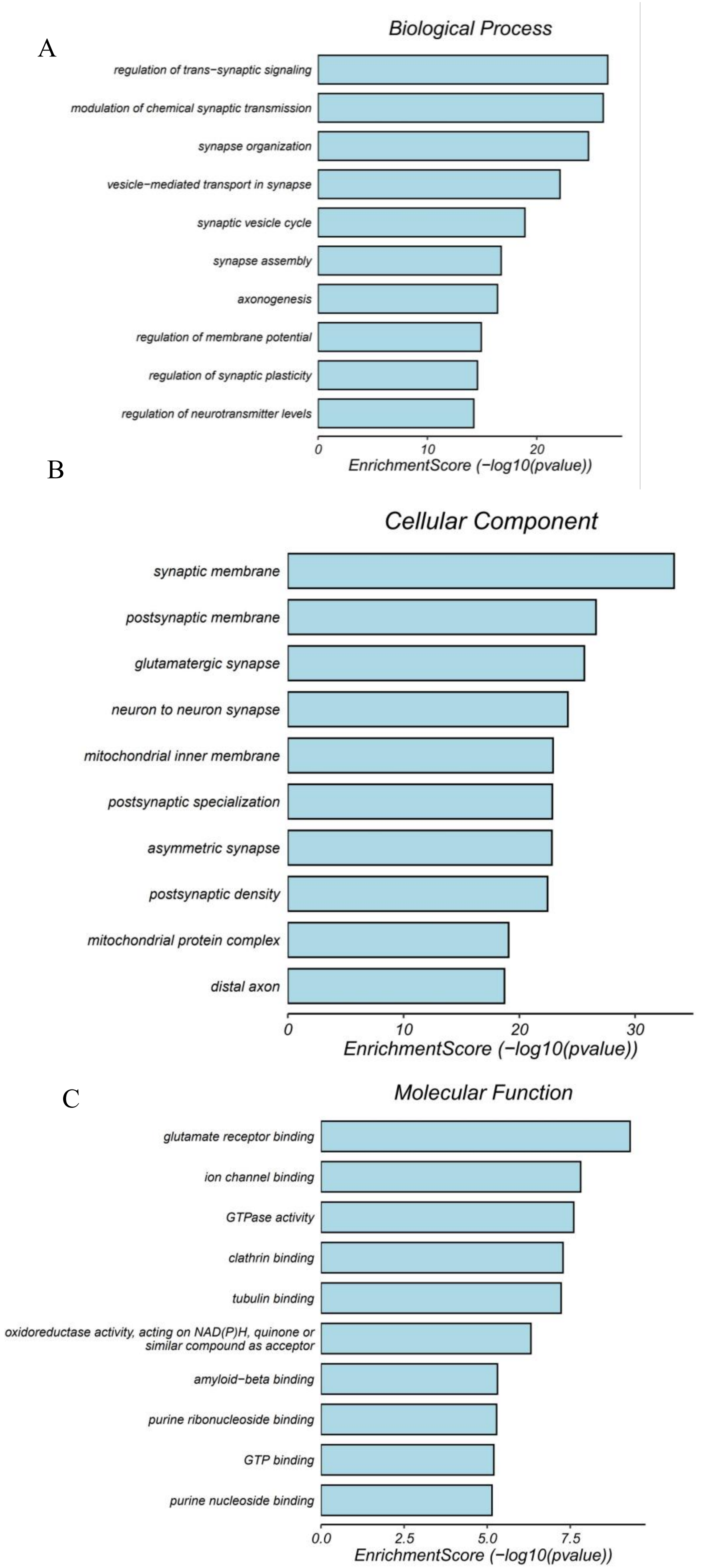

Figure S6. scRNA-seq analysis in DA neurons from control and POLG patient MOs. A. The top ten GO biological process terms are enriched in downregulated DEGs in POLG in MOs of POLG patients compared with controls. B. The top ten GO cellular component term is enriched in downregulated DEGs in MOs of POLG patients compared with controls. C. The top ten GO molecular functional terms are enriched in downregulated DEGs in MOs of POLG patients compared with controls.

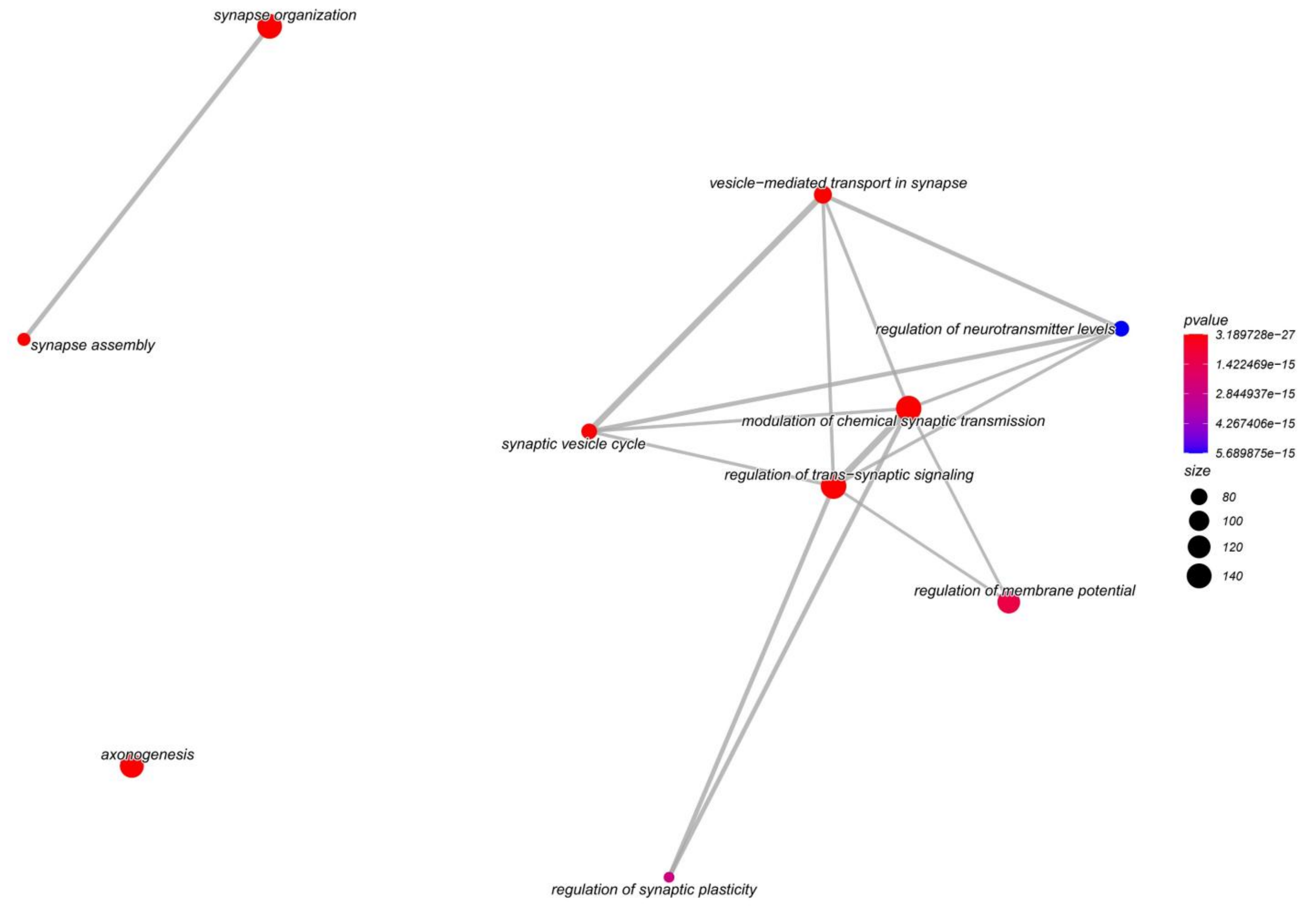

Figure S7. GO biological process emapplot analysis of downregulated DEGs in MOs of POLG patients compared with controls.

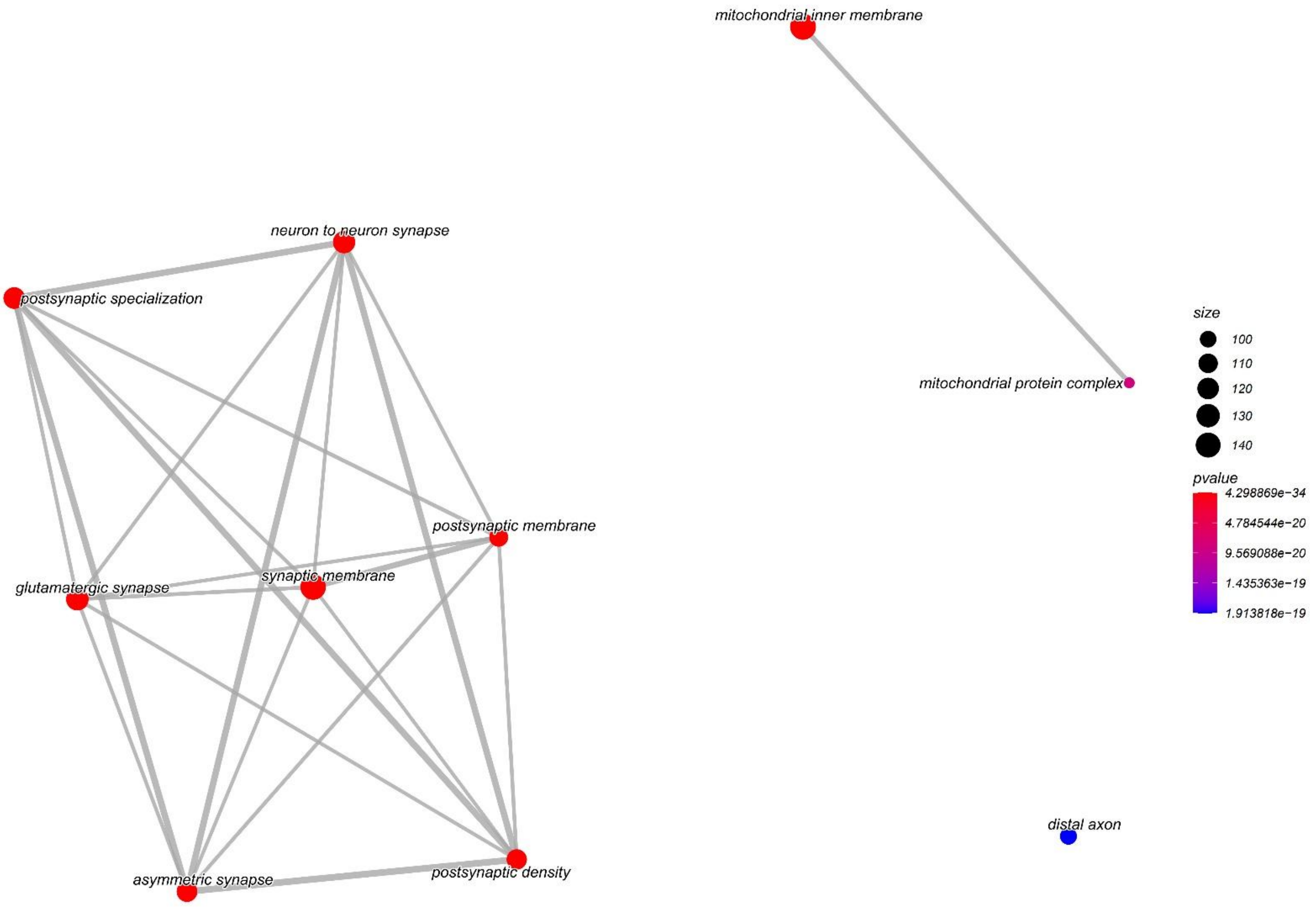

Figure S8. GO cell component emapplot analysis of downregulated DEGs in MOs of POLG patients compared with controls.

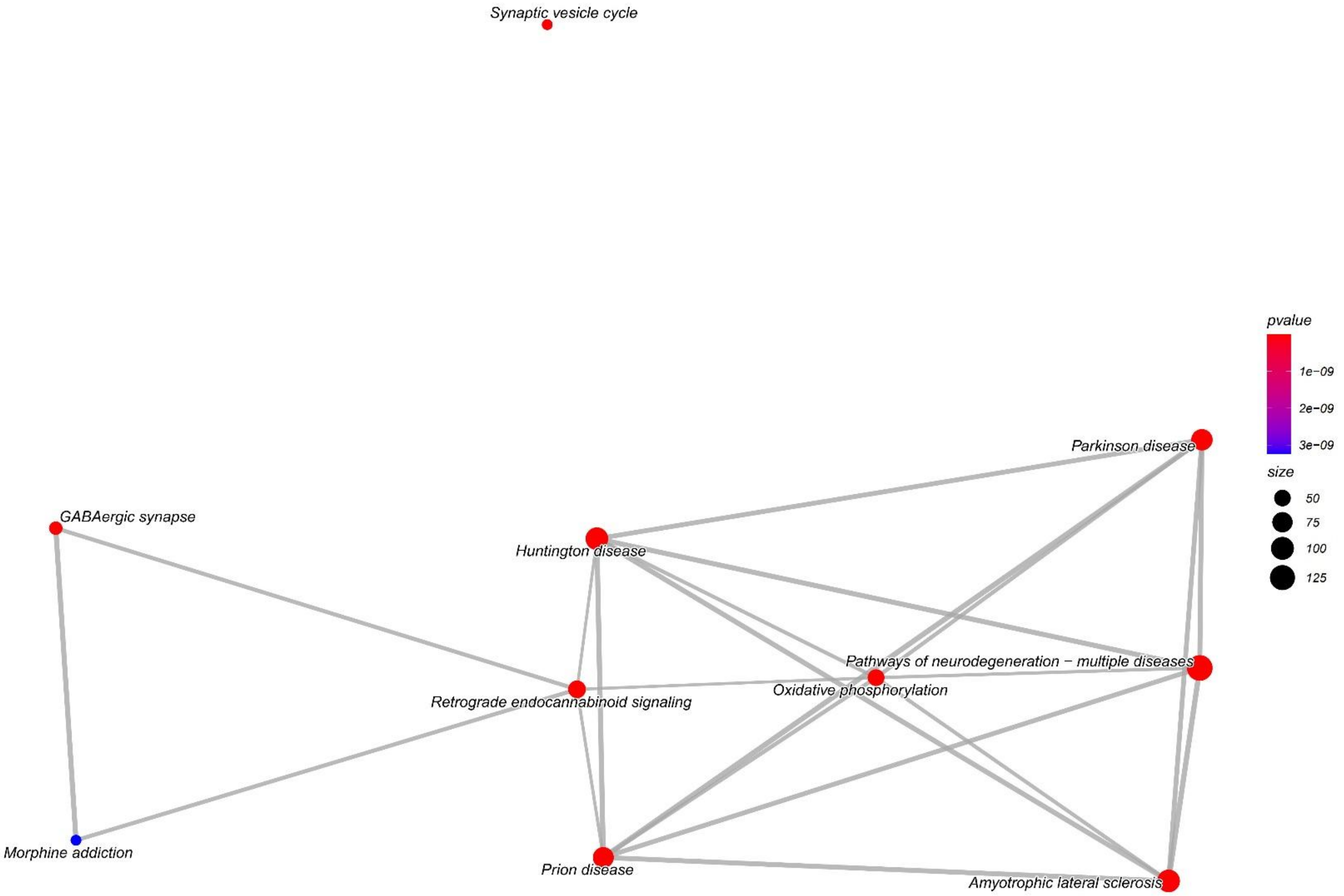

Figure S9. KEGG pathway emapplot analysis of downregulated DEGs in MOs of POLG patients compared with controls.

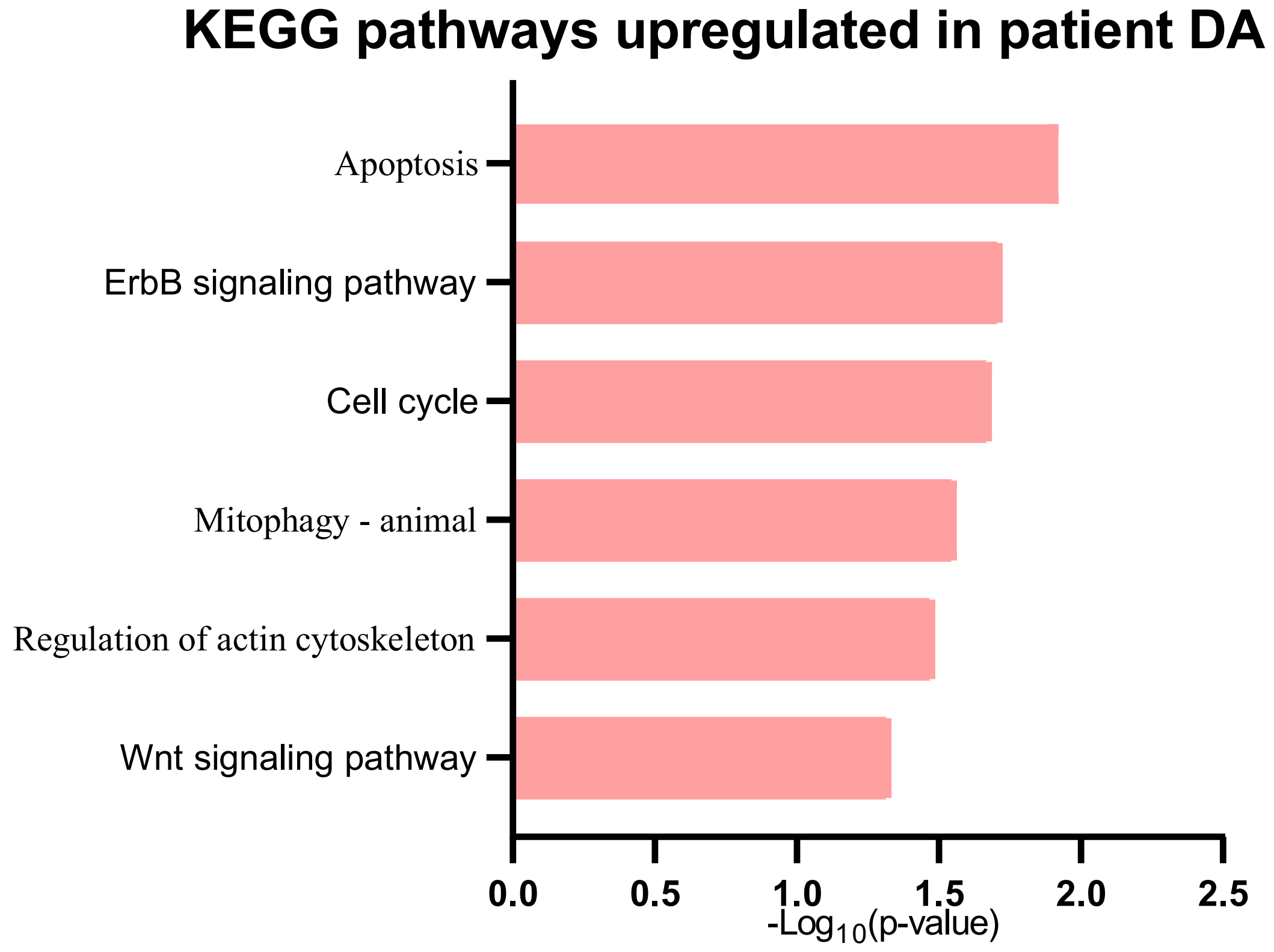

Figure S10. KEGG pathway analysis of upregulated DEGs in DA neurons of MOs from POLG patients compared with controls.

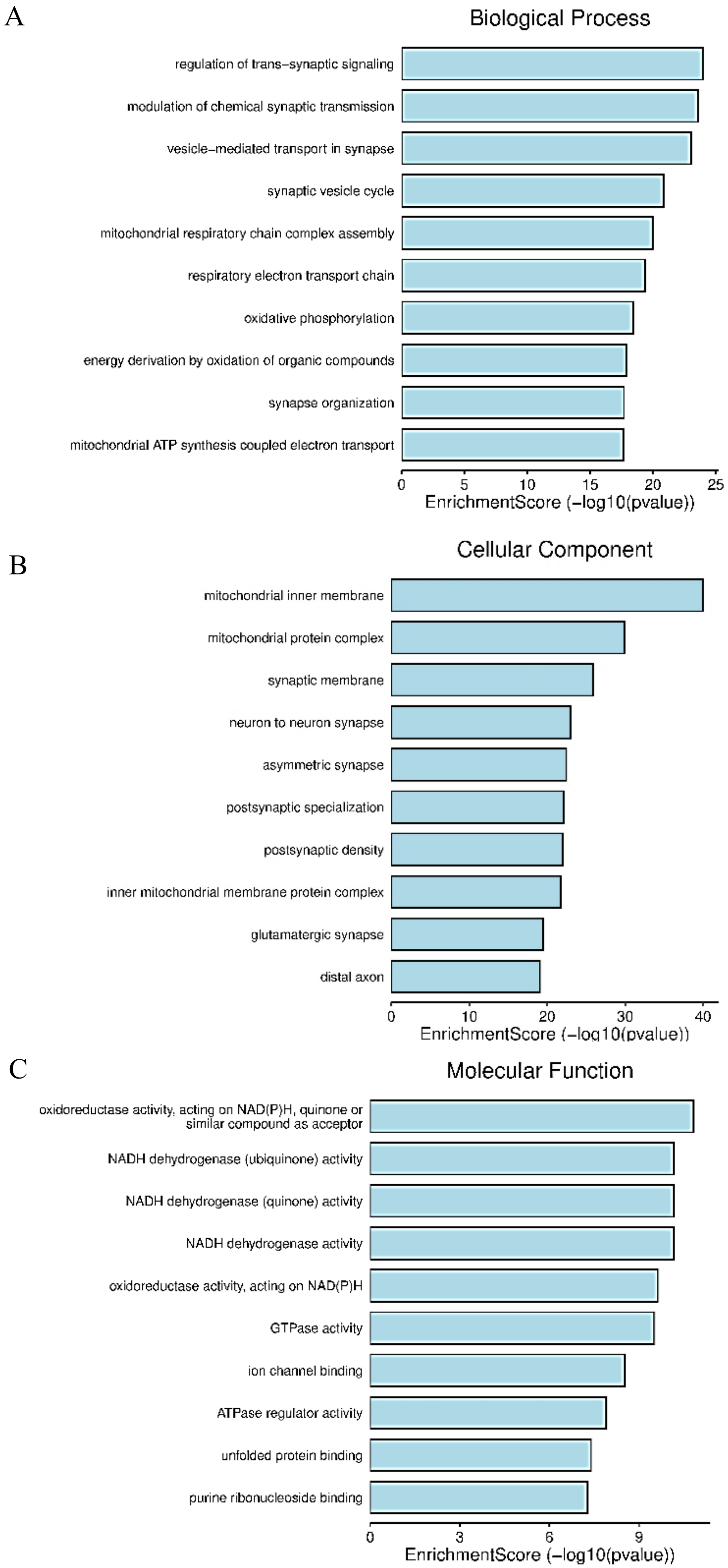

Figure S11. scRNA-seq analysis of DA neurons in NR-treated versus untreated POLG patient MOs.

(A) Top ten enriched GO biological process terms among upregulated DEG) in DA neurons from POLG patient MOs treated with NR compared with untreated controls.(B) Top ten enriched GO cellular component terms among upregulated DEGs in DA neurons from POLG patient MOs treated with NR compared with untreated controls.(C) Top ten enriched GO molecular function terms among upregulated DEGs in DA neurons from POLG patient MOs treated with NR compared with untreated controls.

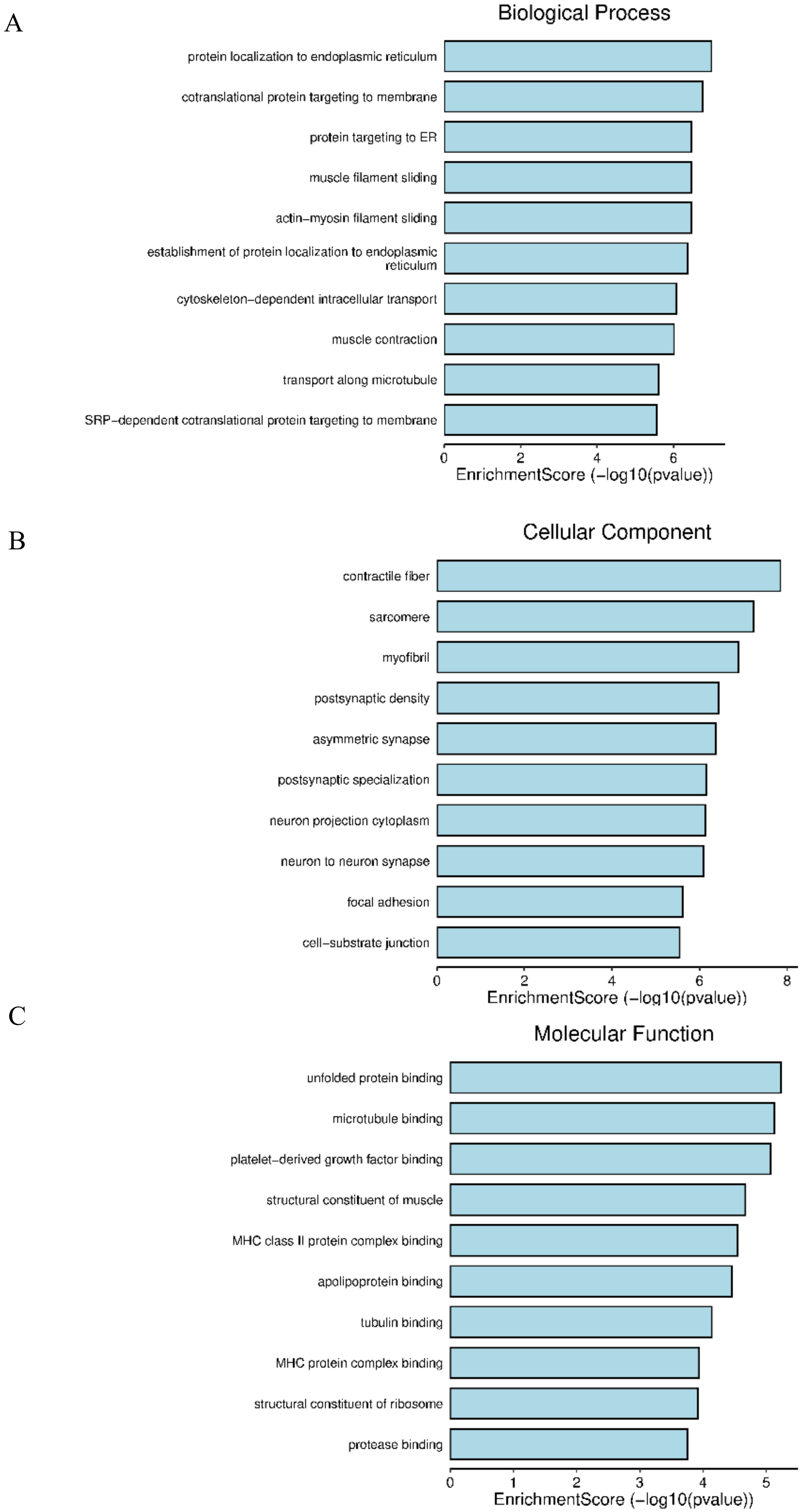

Figure S12. scRNA-seq analysis of DA2 neurons in NR-treated versus untreated POLG patient MOs.

(A) Top ten enriched GO biological process terms among upregulated DEGs in DA2 neurons from POLG patient MOs treated with NR compared with untreated controls.(B) Top ten enriched GO cellular component terms among upregulated DEGs in DA2 neurons from POLG patient MOs treated with NR compared with untreated controls.(C) Top ten enriched GO molecular function terms among upregulated DEGs in DA2 neurons from POLG patient MOs treated with NR compared with untreated controls.

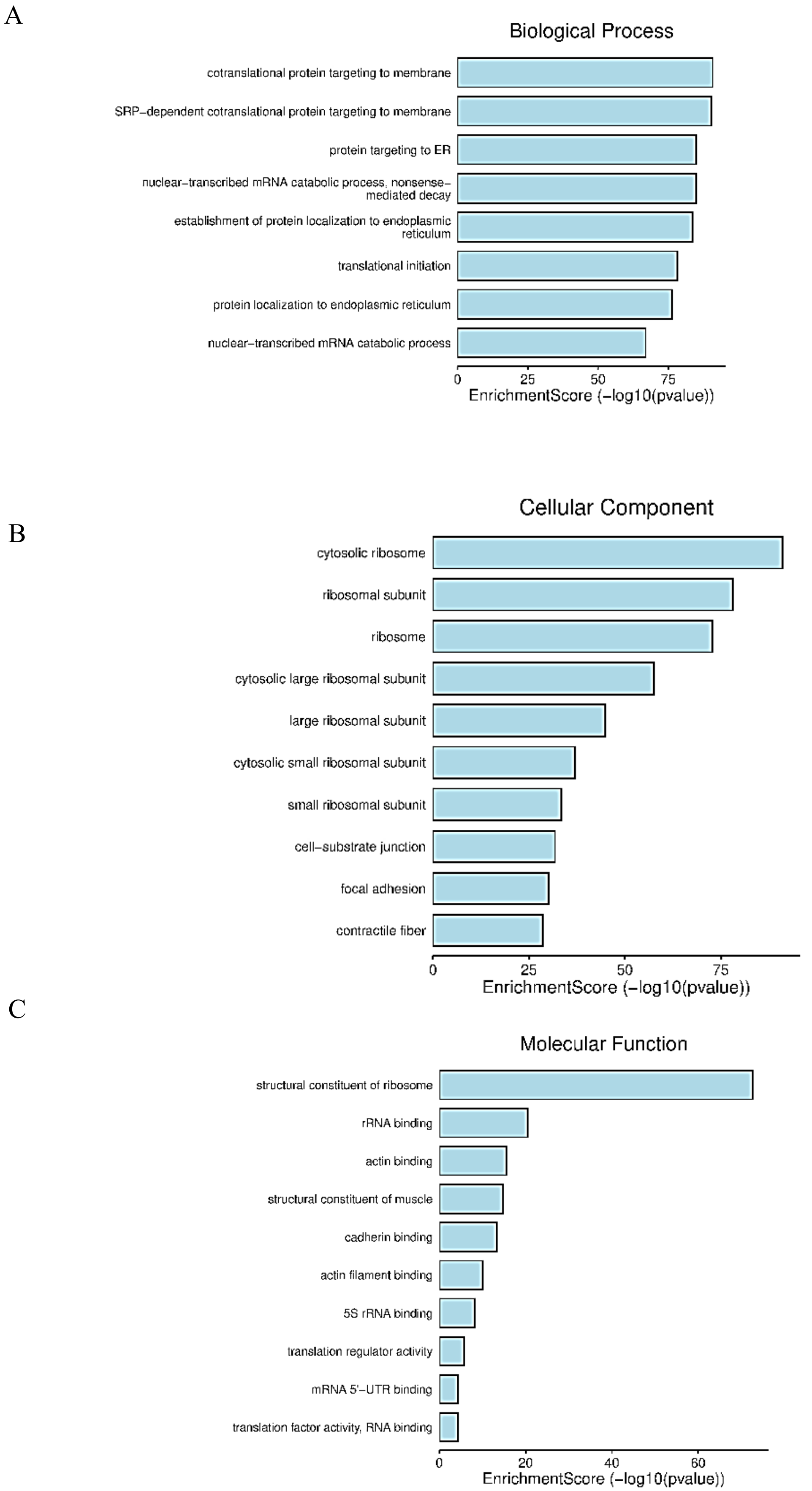

Figure S13. scRNA-seq analysis of VMNs in NR-treated versus untreated POLG patient MOs.

(A) Top ten enriched GO biological process terms among upregulated DEGs in VMN from POLG patient MOs treated with NR compared with untreated controls.(B) Top ten enriched GO cellular component terms among upregulated DEGs in VMNs from POLG patient MOs treated with NR compared with untreated controls.(C) Top ten enriched GO molecular function terms among upregulated DEGs in VMN neurons from POLG patient MOs treated with NR compared with untreated controls.

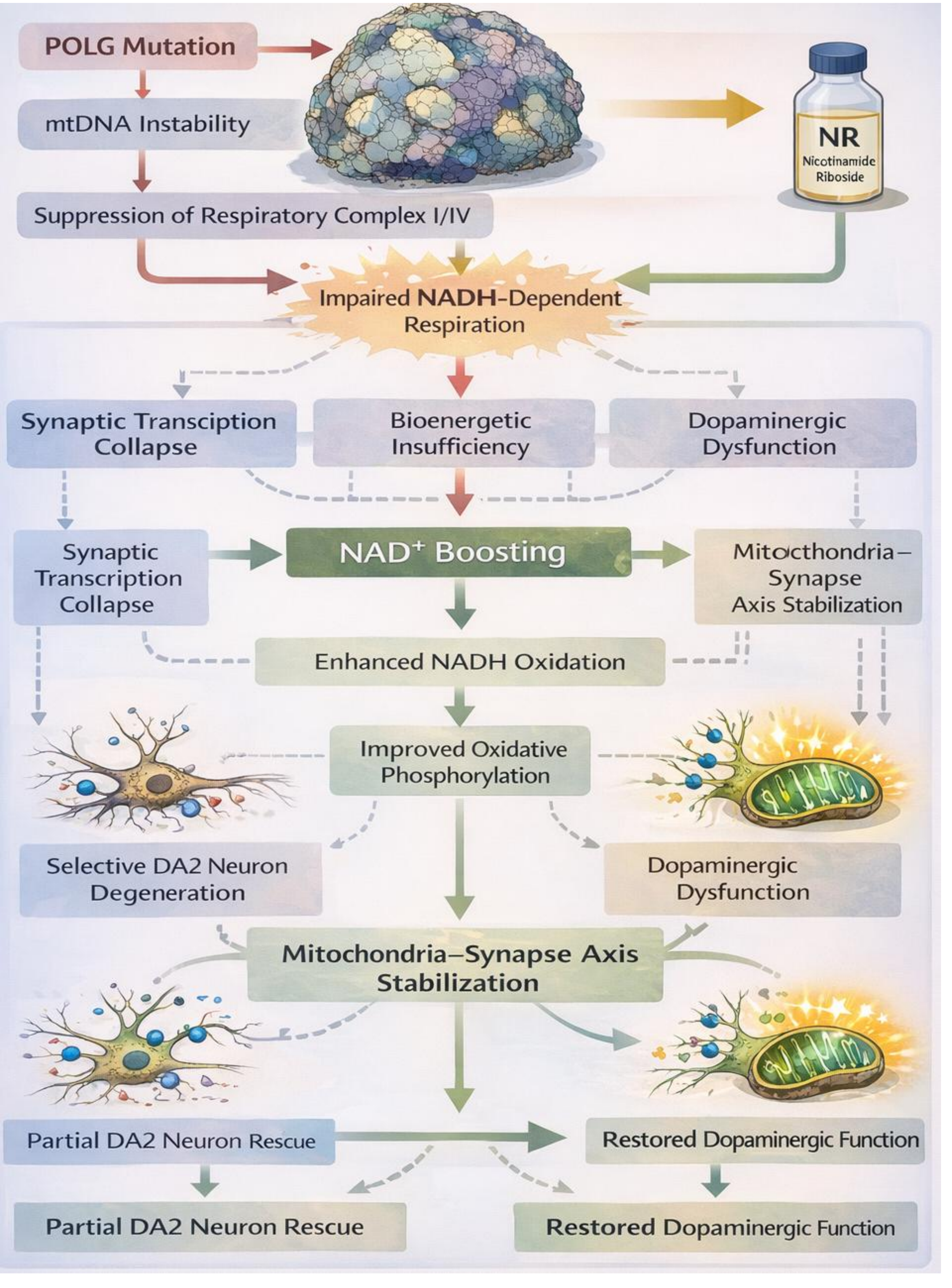

Figure S14. Proposed mitochondria–synapse axis model underlying POLG-associated dopaminergic vulnerability. Adapted from an AI-generated conceptual illustration (ChatGPT, OpenAI) and modified by the authors.
