# Supplementary material for: Impaired NADH-linked mitochondrial respiration disrupts ventral midbrain neuronal programs in POLG disease
Source: J Transl Med. 2026 Jul 31;24:988. doi: 10.1186/s12967-026-08706-w (PMC13435581; doi:10.1186/s12967-026-08706-w)
Supplement: Supplementary file 3 [file 12967_2026_8706_MOESM3_ESM.pdf]

Table S1. List of antibodies used in the study.

Markers	Host	Company	Catalogue Number	Application	Concentration
**Primary antibodies**					
**Nanog**	Rabbit	Abcam	ab80892	ICC	1 : 200
**SSEA4**	Mouse	Abcam	ab16287	ICC	1 : 200
**OCT4**	Rabbit	Abcam	ab19857	ICC	1 : 200
**SOX2**	Rabbit	Abcam	ab97959	ICC	1 : 200
**PAX6**	Rabbit	Abcam	ab5790	ICC	1 : 200
**TUJ1**	Mouse	Abcam	ab78078	ICC	1 : 1000
**MAP2**	Chicken	Abcam	ab5392	ICC	1 : 500
**Synaptophysin**	Rabbit	Abcam	ab32127	ICC	1 : 1000
**PSD-95**	Mouse	Abcam	ab2723	ICC	1 : 1000
**DAT**	Rabbit	Abcam	ab214470	ICC/FC	1 : 100
**TH**	Rabbit	Abcam	ab75875	ICC/FC	1 : 1000
**OTX2**	Rabbit	Proteintech	13497-1-AP	ICC	1 : 200
**FOXA2**	Rabbit	Proteintech	22474-1-AP	ICC	1 : 200
**Secondary antibodies**					
**Alexa Flour® 488**	Rabbit	Thermo Fisher Scientific	A11008	ICC/FC	1 : 400
**Alexa Flour®594**	Mouse	Thermo Fisher Scientific	A11005	ICC	1 : 400
**Alexa Flour®594**	Chicken	Thermo Fisher Scientific	A11042	ICC	1 : 400
**Alexa Flour® 647**	Chicken	Thermo Fisher Scientific	A-21449	ICC	1 : 400
**Alexa Flour® 594**	Rabbit	Thermo Fisher Scientific	A-11012	ICC	1 : 400
**Alexa Flour® 488**	Mouse	Thermo Fisher Scientific	A-11001	ICC	1 : 400

ICC: Immunofluorimetric cytochemistry staining; FC: Flow cytometry

Table S2. List of cell numbers in scRNA-seq.

Sample ID	Estimated Number of Cells	Mean Reads per Cell	Median UMI per Cell	Total Gene	Median Gene per Cell
**control**	5,369	49,258	1,882	24,576	1,010
**patient**	3,331	69,973	3,542	25,389	1,648
**patient with treatment**	8,433	27,269	1,919	26,403	1,071

Table S3: Top 10 mitochondrial related GO/BP terms downregulated in patient DA neurons, related to Figure 3A.

ID	Description	Foldchange	pvalue	p.adjust	qvalue
GO:0033108	mitochondrial respiratory chain complex assembly	12.04612496	8.99239E-13	3.35079E-10	2.87106E-10
GO:0070125	mitochondrial translational elongation	11.01934414	9.56436E-12	2.71043E-09	2.32237E-09
GO:0006839	mitochondrial transport	10.72956475	1.86395E-11	4.43143E-09	3.79698E-09
GO:0046034	ATP metabolic process	10.43781338	3.64911E-11	7.50206E-09	6.42799E-09
GO:0042775	mitochondrial ATP synthesis coupled electron transport	9.548269822	2.82963E-10	3.58942E-08	3.07552E-08
GO:0042773	ATP synthesis coupled electron transport	9.399480352	3.98584E-10	4.65952E-08	3.99242E-08
GO:0015980	energy derivation by oxidation of organic compounds	9.323495727	4.74793E-10	5.44368E-08	4.66431E-08
GO:0032543	mitochondrial translation	9.194270165	6.39337E-10	7.19194E-08	6.16227E-08
GO:0010257	NADH dehydrogenase complex assembly	8.085592472	8.21122E-09	7.41747E-07	6.35551E-07
GO:0032981	mitochondrial respiratory chain complex I assembly	8.085592472	8.21122E-09	7.41747E-07	6.35551E-07

Table S4: Top 10 mitochondrial related GO/CC terms downregulated in patient DA neurons, related to Figure 3B.

ID	Description	Foldchange	pvalue	p.adjust	qvalue
GO:0005759	mitochondrial matrix	9.914822276	1.21668E-10	2.56219E-09	1.64233E-09
GO:0005746	mitochondrial respirasome	8.71406568	1.93168E-09	3.63968E-08	2.33299E-08
GO:0005747	mitochondrial respiratory chain complex I	5.760262975	1.73675E-06	1.85599E-05	1.18967E-05
GO:0030964	NADH dehydrogenase complex	5.760262975	1.73675E-06	1.85599E-05	1.18967E-05
GO:0045271	respiratory chain complex I	5.760262975	1.73675E-06	1.85599E-05	1.18967E-05
GO:0031304	intrinsic component of mitochondrial inner membrane	5.114173502	7.68823E-06	7.04611E-05	4.51647E-05
GO:0031305	integral component of mitochondrial inner membrane	5.114173502	7.68823E-06	7.04611E-05	4.51647E-05
GO:0005762	mitochondrial large ribosomal subunit	4.18638642	6.51049E-05	0.000475664	0.000304895
GO:0005750	mitochondrial respiratory chain complex III	3.924915894	0.000118873	0.000826342	0.000529675
GO:0045275	respiratory chain complex III	3.924915894	0.000118873	0.000826342	0.000529675

Table S5: Mitochondrial related GO/MF terms downregulated in patient DA neurons, related to Figure 3C.

ID	Description	Foldchange	pvalue	p.adjust	qvalue
GO:0016655	oxidoreductase activity, acting on NAD(P)H, quinone or similar compound as acceptor	6.31656154	4.82435E-07	8.84463E-05	7.60046E-05
GO:0008137	NADH dehydrogenase (ubiquinone) activity	4.85200654	1.40603E-05	0.000814015	0.000699508
GO:0003954	NADH dehydrogenase activity	4.85200654	1.40603E-05	0.000814015	0.000699508
GO:0050136	NADH dehydrogenase (quinone) activity	4.85200654	1.40603E-05	0.000814015	0.000699508
GO:0044769	ATPase activity, coupled to transmembrane movement of ions, rotational mechanism	3.98892936	0.000102582	0.004340002	0.003729495
GO:0009055	electron transfer activity	3.52730858	0.000296956	0.009031218	0.007760798
GO:0015078	proton transmembrane transporter activity	3.51744463	0.000303777	0.009031218	0.007760798
GO:0051536	iron-sulfur cluster binding	2.96285957	0.001089282	0.021396616	0.018386757
GO:0042626	ATPase-coupled transmembrane transporter activity	2.82772714	0.00148687	0.026000017	0.022342598
GO:0046933	proton-transporting ATP synthase activity, rotational mechanism	2.02623451	0.009413811	0.093290022	0.080166928

Table S6: Neural related GO/MF terms downregulated in patient DA neurons, related to Figure 3D.

ID	Description	Foldchange	pvalue	p.adjust	qvalue
GO:0035254	glutamate receptor binding	9.30086037	5.00195E-10	5.50215E-07	4.72816E-07
GO:0044325	ion channel binding	7.81422956	1.53381E-08	8.43593E-06	7.24925E-06
GO:0030276	clathrin binding	7.28343369	5.20674E-08	1.31065E-05	1.12628E-05
GO:0035255	ionotropic glutamate receptor binding	4.59338485	2.55044E-05	0.001402742	0.001205419
GO:0051082	unfolded protein binding	4.56431693	2.72699E-05	0.001428422	0.001227486
GO:0008017	microtubule binding	3.64798731	0.000224912	0.008510334	0.007313187
GO:0098918	structural constituent of synapse	3.63704095	0.000230653	0.008510334	0.007313187
GO:0000149	SNARE binding	3.61536355	0.000242458	0.008603347	0.007393115
GO:0043014	alpha-tubulin binding	3.57466123	0.00026628	0.008973183	0.007710926
GO:0030165	PDZ domain binding	3.55765592	0.000276913	0.008973183	0.007710926
GO:0016917	GABA receptor activity	3.55696725	0.000277353	0.008973183	0.007710926

Table S7: Downregulated Complex I-associated DEGs in patient DA neurons, related to Figure 3E.

names	scores	logfoldchanges	pvals	pvals_adj
NDUFS3	-3.12216	-0.25063	0.001795	0.022284
NDUFS2	-3.15529	-0.25835	0.001603	0.020192
NDUFS7	-3.27191	-0.27171	0.001068	0.014277
NDUFS8	-3.35416	-0.25257	0.000796	0.011005
NDUFAF4	-3.67048	-0.33353	0.000242	0.003859
NDUFS6	-3.81911	-0.30412	0.000134	0.002294
NDUFA8	-4.11664	-0.33975	3.84E-05	0.000755
NDUFA12	-4.29843	-0.31825	1.72E-05	0.000363
NDUFB6	-4.43001	-0.33154	9.42E-06	0.000209
NDUFS4	-4.49275	-0.35551	7.03E-06	0.00016
NDUFA10	-4.79955	-0.40189	1.59E-06	4.12E-05
NDUFB3	-4.98469	-0.39094	6.21E-07	1.72E-05
NDUFA5	-5.30996	-0.37134	1.1E-07	3.45E-06
NDUFA2	-5.39184	-0.44909	6.97E-08	2.26E-06
NDUFA4	-5.58753	-0.23205	2.3E-08	8.17E-07
NDUFAF6	-5.91964	-0.85553	3.23E-09	1.31E-07
NDUFAF8	-6.32942	-0.74037	2.46E-10	1.16E-08
NDUFB2	-6.89882	-0.4079	5.24E-12	3.05E-10
NDUFV3	-7.01641	-0.6462	2.28E-12	1.38E-10
NDUFC1	-7.5483	-0.56743	4.41E-14	3.22E-12
NDUFB1	-11.2576	-0.76883	2.12E-29	5.55E-27

Table S8: Downregulated Complex II, III, IV-associated DEGs in patient DA neurons, related to Figure 3F.

names	scores	logfoldchanges	pvals	pvals_adj
SDHB	-3.2993	-0.31882	0.000969	0.013103
SDHC	-5.98162	-0.56566	2.21E-09	9.14E-08
UQCRH	-3.04171	-0.19402	0.002352	0.027998
UQCRHL	-3.12814	-1.15351	0.001759	0.021908
UQCR10	-4.53441	-0.28378	5.78E-06	0.000135
UQCRFS1	-5.03901	-0.39035	4.68E-07	1.33E-05
UQCRQ	-5.05573	-0.29944	4.29E-07	1.23E-05
COX11	-3.03297	-0.23001	0.002422	0.028657
COX7A2L	-4.94137	-0.37868	7.76E-07	2.13E-05
COX6C	-7.09946	-0.37413	1.25E-12	7.81E-11

Table S9: Downregulated Complex V-associated DEGs in patient DA neurons, related to Figure 3F.

names	scores	logfoldchanges	pvals	pvals_adj
ATP5F1D	-2.89184	-0.22838	0.00382991	0.042297
ATP5MD	-3.01594	-0.19085	0.002561862	0.030172
ATP5F1E	-3.95121	-0.24668	7.7757E-05	0.001412
ATP5PD	-4.93228	-0.33315	8.12773E-07	2.22E-05
ATP5PB	-5.42685	-0.38081	5.73588E-08	1.9E-06
ATP5MC3	-5.72407	-0.34083	1.04005E-08	3.92E-07
ATP5ME	-6.70564	-0.41596	2.00522E-11	1.1E-09
ATP5IF1	-8.85409	-0.60682	8.43706E-19	9.89E-17
MT-ATP8	-13.7982	-0.88467	2.61222E-43	1.42E-40

Table S10: GO/BP downregulated in patient DA2 enurons, related to Figure 4E.

ID	Description	Foldchange	pvalue	p.adjust	qvalue
GO:0099177	regulation of trans-synaptic signaling	26.44419811	3.59585E-27	2.07337E-23	1.7684E-23
GO:0050804	modulation of chemical synaptic transmission	25.99561899	1.01014E-26	2.91223E-23	2.48388E-23
GO:0099003	vesicle-mediated transport in synapse	20.01848759	9.58324E-21	1.8419E-17	1.57098E-17
GO:0050808	synapse organization	18.62858667	2.35187E-19	3.39022E-16	2.89156E-16
GO:0099504	synaptic vesicle cycle	16.98650958	1.03155E-17	1.18958E-14	1.01461E-14
GO:0007409	axonogenesis	15.80396249	1.5705E-16	1.42259E-13	1.21334E-13
GO:0001505	regulation of neurotransmitter levels	15.76269763	1.72704E-16	1.42259E-13	1.21334E-13
GO:0006836	neurotransmitter transport	14.14564626	7.15079E-15	5.15393E-12	4.39585E-12
GO:0099643	signal release from synapse	13.15173468	7.05124E-14	4.06574E-11	3.46772E-11
GO:0007269	neurotransmitter secretion	13.15173468	7.05124E-14	4.06574E-11	3.46772E-11

Table S11: GO/CC downregulated in patient DA2 neurons, related to Figure 4F.

ID	Description	Foldchange	pvalue	p.adjust	qvalue
GO:0097060	synaptic membrane	29.95490458	1.10942E-30	7.8214E-28	5.10333E-28
GO:0098978	glutamatergic synapse	22.88542594	1.30189E-23	4.32226E-21	2.8202E-21
GO:0005743	mitochondrial inner membrane	22.73535682	1.83926E-23	4.32226E-21	2.8202E-21
GO:0098984	neuron to neuron synapse	22.15131352	7.05808E-23	1.24399E-20	8.11679E-21
GO:0045211	postsynaptic membrane	21.51052042	3.08659E-22	3.7617E-20	2.45444E-20
GO:0014069	postsynaptic density	21.49465426	3.20144E-22	3.7617E-20	2.45444E-20
GO:0032279	asymmetric synapse	21.4201237	3.80081E-22	3.82796E-20	2.49768E-20
GO:0099572	postsynaptic specialization	20.67454309	2.11571E-21	1.86447E-19	1.21654E-19
GO:0008021	synaptic vesicle	17.83855767	1.45025E-18	1.13603E-16	7.41238E-17
GO:0070382	exocytic vesicle	17.44649726	3.57687E-18	2.52169E-16	1.64536E-16

Table S12: GO/MF downregulated in patient DA2 neurons, related to Figure 4G.

ID	Description	Foldchange	pvalue	p.adjust
GO:0030276	clathrin binding	7.601480761	2.50334E-08	2.66105E-05
GO:0016655	oxidoreductase activity, acting on NAD(P)H, quinone or similar compound as acceptor	7.1433943	7.18796E-08	3.8204E-05
GO:0035254	glutamate receptor binding	6.742585128	1.8089E-07	6.40954E-05
GO:0044325	ion channel binding	6.523042874	2.99887E-07	7.96949E-05
GO:0016651	oxidoreductase activity, acting on NAD(P)H	6.139459253	7.25339E-07	0.000154207
GO:0003924	GTPase activity	5.833848248	1.46606E-06	0.000259737
GO:0001540	amyloid-beta binding	5.109310018	7.77481E-06	0.001180661
GO:0042625	ATPase-coupled ion transmembrane transporter activity	4.963166595	1.08851E-05	0.001446361
GO:0044769	ATPase activity, coupled to transmembrane movement of ions, rotational mechanism	4.690158611	2.04099E-05	0.00198969
GO:0046961	proton-transporting ATPase activity, rotational mechanism	4.690158611	2.04099E-05	0.00198969

Table S13: KEGG pathways downregulated in patient DA2 neurons, related to Figure 4H.

ID	Description	Foldchange	pvalue	p.adjust	qvalue
hsa05012	Parkinson disease	12.4151373	3.8447E-13	1.24568E-10	9.38917E-11
hsa00190	Oxidative phosphorylation	11.6556552	2.20976E-12	3.57981E-10	2.69823E-10
hsa05016	Huntington disease	11.4214299	3.7894E-12	4.09255E-10	3.0847E-10
hsa05022	Pathways of neurodegeneration - multiple diseases	10.9376109	1.15449E-11	9.35135E-10	7.04845E-10
hsa04721	Synaptic vesicle cycle	10.6138553	2.43301E-11	1.57659E-09	1.18834E-09
hsa04727	GABAergic synapse	10.2151429	6.09336E-11	3.29042E-09	2.48011E-09
hsa04723	Retrograde endocannabinoid signaling	9.40627878	3.92393E-10	1.81622E-08	1.36895E-08
hsa05014	Amyotrophic lateral sclerosis	8.69119926	2.03611E-09	7.38145E-08	5.56366E-08
hsa05020	Prion disease	8.6881611	2.0504E-09	7.38145E-08	5.56366E-08
hsa05032	Morphine addiction	7.35032446	4.4635E-08	1.44617E-06	1.09003E-06

Table S14: Mitochondrial related GO/BP downregulated in patient DA2 neurons, related to Figure 5A.

ID	Description	Foldchange	pvalue	p.adjust	qvalue
GO:0070125	mitochondrial translational elongation	11.92004791	1.20213E-12	5.77624E-10	4.92663E-10
GO:0070126	mitochondrial translational termination	11.74994418	1.77851E-12	7.88837E-10	6.72809E-10
GO:0033108	mitochondrial respiratory chain complex assembly	11.1339894	7.34532E-12	2.35295E-09	2.00686E-09
GO:0006119	oxidative phosphorylation	10.23341214	5.84235E-11	1.46465E-08	1.24922E-08
GO:0032543	mitochondrial translation	9.468142884	3.40296E-10	6.14309E-08	5.23952E-08
GO:0046034	ATP metabolic process	8.930912411	1.17243E-09	1.82709E-07	1.55835E-07
GO:0006839	mitochondrial transport	8.687534755	2.05336E-09	2.88773E-07	2.46298E-07
GO:0140053	mitochondrial gene expression	8.141262229	7.22334E-09	9.2555E-07	7.89413E-07
GO:0022904	respiratory electron transport chain	7.539183698	2.88946E-08	3.33212E-06	2.84201E-06
GO:0016236	macroautophagy	7.088020578	8.16544E-08	7.97998E-06	6.80623E-06
GO:0010257	NADH dehydrogenase complex assembly	6.944660978	1.1359E-07	1.03962E-05	8.86702E-06
GO:0032981	mitochondrial respiratory chain complex I assembly	6.944660978	1.1359E-07	1.03962E-05	8.86702E-06
GO:0042775	mitochondrial ATP synthesis coupled electron transport	6.733686432	1.84635E-07	1.58896E-05	1.35525E-05
GO:0042773	ATP synthesis coupled electron transport	6.627807889	2.35609E-07	1.96887E-05	1.67928E-05
GO:0006120	mitochondrial electron transport, NADH to ubiquinone	5.665515553	2.16015E-06	0.000133929	0.00011423
GO:0007006	mitochondrial membrane organization	4.868770195	1.35279E-05	0.000541679	0.000462005
GO:0017062	respiratory chain complex III assembly	4.840369437	1.44421E-05	0.00055888	0.000476676
GO:0034551	mitochondrial respiratory chain complex III assembly	4.840369437	1.44421E-05	0.00055888	0.000476676
GO:0098779	positive regulation of mitophagy in response to mitochondrial depolarization	4.840369437	1.44421E-05	0.00055888	0.000476676
GO:1990542	mitochondrial transmembrane transport	4.787762095	1.63019E-05	0.000618399	0.000527441
GO:0010821	regulation of mitochondrion organization	4.605950906	2.4777E-05	0.000855475	0.000729645
GO:1904923	regulation of autophagy of mitochondrion in response to mitochondrial depolarization	4.256440005	5.54064E-05	0.00157376	0.00134228
GO:0000422	autophagy of mitochondrion	4.206240914	6.21955E-05	0.001691601	0.001442788
GO:0061726	mitochondrion disassembly	4.206240914	6.21955E-05	0.001691601	0.001442788
GO:0006754	ATP biosynthetic process	3.942983782	0.000114029	0.002728185	0.002326904
GO:0046902	regulation of mitochondrial membrane permeability	2.964092036	0.001086195	0.016612739	0.014169217
GO:0033617	mitochondrial cytochrome c oxidase assembly	2.859161782	0.001383051	0.020036866	0.017089697
GO:0042776	mitochondrial ATP synthesis coupled proton transport	2.582235978	0.002616761	0.032102644	0.027380753
GO:0006734	NADH metabolic process	2.501790529	0.003149267	0.037517919	0.031999511
GO:0000423	mitophagy	2.457034894	0.003491123	0.040502642	0.034545219
GO:0008535	respiratory chain complex IV assembly	2.339548356	0.004575638	0.048409409	0.041289001
GO:0051560	mitochondrial calcium ion homeostasis	2.339548356	0.004575638	0.048409409	0.041289001
GO:0051654	establishment of mitochondrion localization	2.339548356	0.004575638	0.048409409	0.041289001
GO:0097345	mitochondrial outer membrane permeabilization	2.334918586	0.004624677	0.048749338	0.041578931
GO:0002082	regulation of oxidative phosphorylation	2.282140275	0.005222275	0.053106943	0.045295587
GO:0006735	NADH regeneration	2.229075507	0.005900985	0.057377872	0.048938317
GO:0015986	ATP synthesis coupled proton transport	2.229075507	0.005900985	0.057377872	0.048938317
GO:1902686	mitochondrial outer membrane permeabilization involved in programmed cell death	2.218310979	0.006049076	0.058521763	0.049913956
GO:0006851	mitochondrial calcium ion transmembrane transport	1.865608877	0.013626713	0.107045817	0.091300739
GO:0019674	NAD metabolic process	1.818102048	0.015201903	0.115791508	0.09876005
GO:1903955	positive regulation of protein targeting to mitochondrion	1.762982725	0.017259065	0.123163083	0.10504736
GO:1901028	regulation of mitochondrial outer membrane permeabilization involved in apoptotic signaling pathway	1.725226216	0.018826682	0.13222247	0.112774226
GO:0090151	establishment of protein localization to mitochondrial membrane	1.483880993	0.032818521	0.19368638	0.165197577
GO:0090199	regulation of release of cytochrome c from mitochondria	1.483880993	0.032818521	0.19368638	0.165197577
GO:0043462	regulation of ATPase activity	1.394999672	0.040271734	0.227877152	0.194359321
GO:0034982	mitochondrial protein processing	1.369584832	0.042698751	0.233365873	0.199040721

Table S15: Mitochondrial related GO/CC downregulated in patient DA2 neurons, related to Figure 5B.

ID	Description	Foldchange	pvalue	p.adjust	qvalue
GO:0005743	mitochondrial inner membrane	22.73535682	1.83926E-23	4.32226E-21	2.8202E-21
GO:0098798	mitochondrial protein complex	16.2279777	5.91592E-17	3.4756E-15	2.26777E-15
GO:0098800	inner mitochondrial membrane protein complex	12.58265279	2.61425E-13	9.70025E-12	6.32924E-12
GO:0005759	mitochondrial matrix	9.077372388	8.36811E-10	1.90307E-08	1.24172E-08
GO:0005761	mitochondrial ribosome	7.788330606	1.62806E-08	3.04985E-07	1.98997E-07
GO:0005746	mitochondrial respirasome	6.934781708	1.16203E-07	1.90519E-06	1.2431E-06
GO:0098803	respiratory chain complex	6.220021697	6.02529E-07	8.13922E-06	5.3107E-06
GO:0005762	mitochondrial large ribosomal subunit	5.972841417	1.06453E-06	1.23032E-05	8.02762E-06
GO:0030964	NADH dehydrogenase complex	4.331492675	4.6613E-05	0.000369238	0.000240921
GO:0005741	mitochondrial outer membrane	3.127215116	0.000746079	0.004109264	0.002681222
GO:0005758	mitochondrial intermembrane space	2.704791868	0.001973368	0.009337078	0.006092278
GO:0005763	mitochondrial small ribosomal subunit	2.683955874	0.002070352	0.009602618	0.006265538
GO:0005750	mitochondrial respiratory chain complex III	2.648316419	0.002247417	0.010156594	0.006626998
GO:0045275	respiratory chain complex III	2.648316419	0.002247417	0.010156594	0.006626998
GO:0008076	voltage-gated potassium channel complex	2.234861549	0.005822888	0.023457921	0.015305878
GO:0000276	mitochondrial proton-transporting ATP synthase complex, coupling factor F(o)	2.210946679	0.006152524	0.024232008	0.015810956
GO:0005753	mitochondrial proton-transporting ATP synthase complex	2.163635727	0.006860634	0.026430313	0.01724531
GO:0005744	TIM23 mitochondrial import inner membrane translocase complex	1.888414912	0.0129296	0.044683176	0.02915498
GO:0001518	voltage-gated sodium channel complex	1.517584095	0.03036798	0.087030187	0.056785654

Table S16: Mitochondrial related GO/MF downregulated in patient DA2 neurons, related to Figure 5C.

ID	Description	Foldchange	pvalue	p.adjust	qvalue
GO:0016655	oxidoreductase activity, acting on NAD(P)H, quinone or similar compound as acceptor	7.1433943	7.18796E-08	3.8204E-05	3.28755E-05
GO:0016651	oxidoreductase activity, acting on NAD(P)H	6.139459253	7.25339E-07	0.000154207	0.000132699
GO:0003924	GTPase activity	5.833848248	1.46606E-06	0.000259737	0.00022351
GO:0042625	ATPase-coupled ion transmembrane transporter activity	4.963166595	1.08851E-05	0.001446361	0.001244628
GO:0044769	ATPase activity, coupled to transmembrane movement of ions, rotational mechanism	4.690158611	2.04099E-05	0.00198969	0.001712175
GO:0046961	proton-transporting ATPase activity, rotational mechanism	4.690158611	2.04099E-05	0.00198969	0.001712175
GO:0060590	ATPase regulator activity	4.686355218	2.05895E-05	0.00198969	0.001712175
GO:0009055	electron transfer activity	4.532559094	2.93387E-05	0.002399003	0.002064399
GO:0019829	ATPase-coupled cation transmembrane transporter activity	4.254405882	5.56665E-05	0.003866617	0.003327316
GO:0003954	NADH dehydrogenase activity	4.016744727	9.62178E-05	0.005113974	0.004400697
GO:0008137	NADH dehydrogenase (ubiquinone) activity	4.016744727	9.62178E-05	0.005113974	0.004400697
GO:0050136	NADH dehydrogenase (quinone) activity	4.016744727	9.62178E-05	0.005113974	0.004400697
GO:0016620	oxidoreductase activity, acting on the aldehyde or oxo group of donors, NAD or NADP as acceptor	3.552607215	0.000280151	0.00883875	0.007605955
GO:0001671	ATPase activator activity	2.964304875	0.001085663	0.025645778	0.022068803
GO:0042626	ATPase-coupled transmembrane transporter activity	2.82635423	0.001491577	0.030583551	0.026317874
GO:0016903	oxidoreductase activity, acting on the aldehyde or oxo group of donors	2.659251269	0.002191537	0.03819022	0.032863595
GO:0016668	oxidoreductase activity, acting on a sulfur group of donors, NAD(P) as acceptor	2.140975724	0.007228102	0.093382621	0.080357972
GO:0046933	proton-transporting ATP synthase activity, rotational mechanism	1.822575435	0.015046122	0.141870629	0.122083058
GO:0051117	ATPase binding	1.744882863	0.017993562	0.163138174	0.140384288
GO:0043531	ADP binding	1.644944438	0.022649341	0.173210425	0.1490517
GO:0070402	NADPH binding	1.565458513	0.027198283	0.193182426	0.166238083
GO:0016627	oxidoreductase activity, acting on the CH-CH group of donors	1.527880073	0.029656502	0.203386205	0.175018678
GO:0004032	alditol:NADP+ 1-oxidoreductase activity	1.422265547	0.037821126	0.225864364	0.19436167
GO:0051287	NAD binding	1.391251512	0.040620801	0.23856305	0.205289192

Table S17: Neural related GO/BP downregulated in patient DA2 neurons, related to Figure 5D.

ID	Description	Foldchange	pvalue	p.adjust	qvalue
GO:0099177	regulation of trans-synaptic signaling	26.44419811	3.59585E-27	2.07337E-23	1.7684E-23
GO:0050804	modulation of chemical synaptic transmission	25.99561899	1.01014E-26	2.91223E-23	2.48388E-23
GO:0099003	vesicle-mediated transport in synapse	20.01848759	9.58324E-21	1.8419E-17	1.57098E-17
GO:0050808	synapse organization	18.62858667	2.35187E-19	3.39022E-16	2.89156E-16
GO:0099504	synaptic vesicle cycle	16.98650958	1.03155E-17	1.18958E-14	1.01461E-14
GO:0007409	axonogenesis	15.80396249	1.5705E-16	1.42259E-13	1.21334E-13
GO:0001505	regulation of neurotransmitter levels	15.76269763	1.72704E-16	1.42259E-13	1.21334E-13
GO:0006836	neurotransmitter transport	14.14564626	7.15079E-15	5.15393E-12	4.39585E-12
GO:0007269	neurotransmitter secretion	13.15173468	7.05124E-14	4.06574E-11	3.46772E-11
GO:0099643	signal release from synapse	13.15173468	7.05124E-14	4.06574E-11	3.46772E-11
GO:0048167	regulation of synaptic plasticity	11.9752467	1.05865E-12	5.54926E-10	4.73304E-10
GO:0043624	cellular protein complex disassembly	11.44204048	3.61376E-12	1.48835E-09	1.26944E-09
GO:0045666	positive regulation of neuron differentiation	11.36490663	4.31612E-12	1.55542E-09	1.32664E-09
GO:1990138	neuron projection extension	11.1703509	6.75537E-12	2.29126E-09	1.95425E-09
GO:0010976	positive regulation of neuron projection development	10.79993009	1.58515E-11	4.81051E-09	4.10294E-09
GO:0007416	synapse assembly	9.98072478	1.04538E-10	2.31834E-08	1.97734E-08
GO:0036465	synaptic vesicle recycling	9.809124946	1.55194E-10	3.31426E-08	2.82677E-08
GO:0098693	regulation of synaptic vesicle cycle	9.519814115	3.02124E-10	6.00707E-08	5.1235E-08
GO:0048488	synaptic vesicle endocytosis	8.855097228	1.39606E-09	2.06401E-07	1.76042E-07
GO:0140238	presynaptic endocytosis	8.855097228	1.39606E-09	2.06401E-07	1.76042E-07
GO:0016079	synaptic vesicle exocytosis	8.703569932	1.97893E-09	2.85263E-07	2.43304E-07
GO:0050769	positive regulation of neurogenesis	8.430256727	3.71316E-09	5.09763E-07	4.34784E-07
GO:0050770	regulation of axonogenesis	8.310064536	4.89706E-09	6.56662E-07	5.60075E-07
GO:0051588	regulation of neurotransmitter transport	8.239357394	5.76292E-09	7.55204E-07	6.44124E-07
GO:0099173	postsynapse organization	7.947854818	1.12757E-08	1.41339E-06	1.2055E-06
GO:0046928	regulation of neurotransmitter secretion	7.471902171	3.37363E-08	3.74084E-06	3.19061E-06
GO:0050803	regulation of synapse structure or activity	7.443434515	3.60218E-08	3.84633E-06	3.28058E-06
GO:2000300	regulation of synaptic vesicle exocytosis	6.904384309	1.24628E-07	1.12282E-05	9.57668E-06
GO:0050807	regulation of synapse organization	6.150371988	7.0734E-07	5.09815E-05	4.34828E-05
GO:0001764	neuron migration	5.473449154	3.36164E-06	0.00018286	0.000155964
GO:0099601	regulation of neurotransmitter receptor activity	5.25924668	5.50495E-06	0.000262327	0.000223742
GO:0045665	negative regulation of neuron differentiation	4.910933592	1.22763E-05	0.000512935	0.000437488
GO:1901214	regulation of neuron death	4.90179509	1.25373E-05	0.000520074	0.000443577
GO:0099560	synaptic membrane adhesion	4.882249198	1.31145E-05	0.000531063	0.00045295
GO:0014046	dopamine secretion	3.754781883	0.000175881	0.003769992	0.003215474
GO:0014059	regulation of dopamine secretion	3.754781883	0.000175881	0.003769992	0.003215474

Table S18: Mitochondrial related GO/BP downregulated in patient VMN, related to Figure 6C.

ID	Description	Foldchange	pvalue	p.adjust	qvalue
GO:0006735	NADH regeneration	3.43315153	0.000368849	0.009113641	0.006551921
GO:0061718	glucose catabolic process to pyruvate	3.43315153	0.000368849	0.009113641	0.006551921
GO:0006007	glucose catabolic process	3.18160241	0.00065826	0.014457344	0.010393582
GO:0006734	NADH metabolic process	3.06850889	0.000854065	0.017464164	0.012555226
GO:0019674	NAD metabolic process	2.89666955	0.001268617	0.025076325	0.018027712
GO:0031647	regulation of protein stability	2.44659459	0.003576065	0.054374528	0.03909059
GO:0043044	ATP-dependent chromatin remodeling	2.3932271	0.004043644	0.058338374	0.041940253
GO:0046034	ATP metabolic process	2.38637372	0.004107961	0.058338374	0.041940253
GO:0006094	gluconeogenesis	2.38385108	0.004131892	0.058338374	0.041940253
GO:0006096	glycolytic process	2.18613757	0.00651422	0.083579031	0.060086106
GO:0006757	ATP generation from ADP	2.1788595	0.006624308	0.083579031	0.060086106
GO:0046031	ADP metabolic process	2.12288871	0.007535486	0.093094655	0.066927017
GO:0006090	pyruvate metabolic process	1.95308963	0.011140646	0.099826661	0.071766749
GO:2001171	positive regulation of ATP biosynthetic process	1.80161852	0.015789976	0.099826661	0.071766749
GO:2001169	regulation of ATP biosynthetic process	1.67777124	0.021000458	0.114643569	0.082418825
GO:0070570	regulation of neuron projection regeneration	1.47626778	0.033398904	0.132037001	0.094923201

Table S19: Mitochondrial related GO/MF downregulated in patient VMN, related to Figure 6D.

ID	Description	Foldchange	pvalue	p.adjust	qvalue
GO:0016655	oxidoreductase activity, acting on NAD(P)H, quinone or similar compound as acceptor	7.1433943	7.18796E-08	3.8204E-05	3.28755E-05
GO:0016651	oxidoreductase activity, acting on NAD(P)H	6.139459253	7.25339E-07	0.000154207	GO:0016651
GO:0003924	GTPase activity	5.833848248	1.46606E-06	0.000259737	GO:0003924
GO:0042625	ATPase-coupled ion transmembrane transporter activity	4.963166595	1.08851E-05	0.001446361	0.001244628
GO:0044769	ATPase activity, coupled to transmembrane movement of ions, rotational mechanism	4.690158611	2.04099E-05	0.00198969	0.001712175
GO:0046961	proton-transporting ATPase activity, rotational mechanism	4.690158611	2.04099E-05	0.00198969	0.001712175
GO:0060590	ATPase regulator activity	4.686355218	2.05895E-05	0.00198969	0.001712175
GO:0009055	electron transfer activity	4.532559094	2.93387E-05	0.002399003	0.002064399
GO:0019829	ATPase-coupled cation transmembrane transporter activity	4.254405882	5.56665E-05	0.003866617	0.003327316
GO:0003954	NADH dehydrogenase activity	4.016744727	9.62178E-05	0.005113974	0.004400697
GO:0008137	NADH dehydrogenase (ubiquinone) activity	4.016744727	9.62178E-05	0.005113974	0.004400697
GO:0050136	NADH dehydrogenase (quinone) activity	4.016744727	9.62178E-05	0.005113974	0.004400697
GO:0016620	oxidoreductase activity, acting on the aldehyde or oxo group of donors, NAD or NADP as acceptor	3.552607215	0.000280151	0.00883875	0.007605955
GO:0001671	ATPase activator activity	2.964304875	0.001085663	0.025645778	0.022068803
GO:0042626	ATPase-coupled transmembrane transporter activity	2.82635423	0.001491577	0.030583551	0.026317874
GO:0016903	oxidoreductase activity, acting on the aldehyde or oxo group of donors	2.659251269	0.002191537	0.03819022	0.032863595
GO:0016668	oxidoreductase activity, acting on a sulfur group of donors, NAD(P) as acceptor	2.140975724	0.007228102	0.093382621	0.080357972
GO:0046933	proton-transporting ATP synthase activity, rotational mechanism	1.822575435	0.015046122	0.141870629	0.122083058
GO:0051117	ATPase binding	1.744882863	0.017993562	0.163138174	0.140384288
GO:0043531	ADP binding	1.644944438	0.022649341	0.173210425	0.1490517
GO:0070402	NADPH binding	1.565458513	0.027198283	0.193182426	0.166238083
GO:0016627	oxidoreductase activity, acting on the CH-CH group of donors	1.527880073	0.029656502	0.203386205	0.175018678
GO:0004032	alditol:NADP+ 1-oxidoreductase activity	1.422265547	0.037821126	0.225864364	0.19436167
GO:0051287	NAD binding	1.391251512	0.040620801	0.23856305	0.205289192

Table S20: Neural related GO/BP downregulated in patient VMN, related to Figure 6E.

ID	Description	Foldchange	pvalue	p.adjust	qvalue
GO:0048680	positive regulation of axon regeneration	1.93544341	0.011602634	0.099826661	0.071766749
GO:1904396	regulation of neuromuscular junction development	1.93544341	0.011602634	0.099826661	0.071766749
GO:0021794	thalamus development	1.86332964	0.013698416	0.099826661	0.071766749
GO:0060221	retinal rod cell differentiation	1.86332964	0.013698416	0.099826661	0.071766749
GO:0098974	postsynaptic actin cytoskeleton organization	1.86332964	0.013698416	0.099826661	0.071766749
GO:0150105	protein localization to cell-cell junction	1.86332964	0.013698416	0.099826661	0.071766749
GO:1900272	negative regulation of long-term synaptic potentiation	1.86332964	0.013698416	0.099826661	0.071766749
GO:0099188	postsynaptic cytoskeleton organization	1.80161852	0.015789976	0.099826661	0.071766749
GO:0048714	positive regulation of oligodendrocyte differentiation	1.61772792	0.024114157	0.115446679	0.082996192
GO:0140058	neuron projection arborization	1.56513668	0.027218446	0.116118982	0.08347952
GO:0048679	regulation of axon regeneration	1.51836603	0.030313353	0.124832071	0.089743479
GO:1902692	regulation of neuroblast proliferation	1.45037452	0.035450754	0.134758315	0.096879591
GO:0060251	regulation of glial cell proliferation	1.43800319	0.036475127	0.135185938	0.097187015
GO:0048713	regulation of oligodendrocyte differentiation	1.38109979	0.041581506	0.145904337	0.104892618
GO:0001941	postsynaptic membrane organization	1.37059354	0.042599692	0.146869868	0.105586752
GO:0045687	positive regulation of glial cell differentiation	1.34057741	0.045648088	0.15038509	0.108113893
GO:0014014	negative regulation of gliogenesis	1.32170723	0.047675227	0.15281843	0.109863254
GO:1900271	regulation of long-term synaptic potentiation	1.30365869	0.049698275	0.156471036	0.112489162

Table S21: Neural related GO/MF downregulated in patient VMN, related to Figure 6F.

ID	Description	Foldchange	pvalue	p.adjust	qvalue
GO:0099602	neurotransmitter receptor regulator activity	1.971859994	0.0106694	0.113415724	0.097596935
GO:0099529	neurotransmitter receptor activity involved in regulation of postsynaptic membrane potential	1.672143295	0.02127437	0.172796916	0.148695866

Table S22: Upregulated mitochondrial-associated DEGs in NR-treated DA neurons, related to Figure 7F.

names	scores	logfoldchanges	pvals	pvals_adj
CYCS	7.314146	0.434392	2.59E-13	3.69E-10
MT-CO3	7.101503	0.261849	1.23E-12	1.58E-09
MT-ND3	6.270302	0.237949	3.6E-10	2.97E-07
MT-ND4	5.360192	0.204843	8.31E-08	3.95E-05
MT-CO2	5.045653	0.242024	4.52E-07	0.000171
MT-ATP6	4.770379	0.156808	1.84E-06	0.000545
COX7B	4.157959	0.31659	3.21E-05	0.0067
HSPA9	4.044737	0.371132	5.24E-05	0.010327
COX7A2	3.783788	0.129612	0.000154	0.02626
NDUFA6	3.648387	0.338665	0.000264	0.039913
NDUFB9	3.610932	0.239372	0.000305	0.044519

Table S23: Upregulated neural-associated DEGs in NR-treated DA neurons, related to Figure 7G.

names	scores	logfoldchanges	pvals	pvals_adj
LGI2	6.935834	1.31948	4.04E-12	4.83E-09
CADM2	5.226219	0.580889	1.73E-07	7.73E-05
KCND3	4.73873	1.138861	2.15E-06	0.000623
KIF1A	4.650826	0.312396	3.31E-06	0.000908
PCDH9	4.617893	0.648898	3.88E-06	0.001049
TCF4	4.60885	0.562481	4.05E-06	0.001072
CNTN1	4.356087	0.461192	1.32E-05	0.003011
GPRIN3	4.282652	0.74167	1.85E-05	0.004074
DCX	3.62916	0.147062	0.000284	0.041987

Table S24: Mitochondrial related GO/BP upregulated in patient DA neurons with NR, related to Figure 8A.

ID	Description	Foldchange	pvalue	p.adjust	qvalue
GO:0033108	mitochondrial respiratory chain complex assembly	19.95085471	1.11981E-20	1.32227E-17	1.11345E-17
GO:0022904	respiratory electron transport chain	19.33544412	4.61908E-20	4.54518E-17	3.82736E-17
GO:0006119	oxidative phosphorylation	18.43590277	3.6652E-19	3.09133E-16	2.60312E-16
GO:0042775	mitochondrial ATP synthesis coupled electron transport	17.67467623	2.11507E-18	1.24873E-15	1.05152E-15
GO:0042773	ATP synthesis coupled electron transport	17.45106118	3.53947E-18	1.89973E-15	1.59971E-15
GO:0045333	cellular respiration	17.31470085	4.84506E-18	2.38377E-15	2.0073E-15
GO:0070125	mitochondrial translational elongation	16.32470319	4.73475E-17	2.1503E-14	1.81071E-14
GO:0070126	mitochondrial translational termination	16.09896975	7.96215E-17	3.35775E-14	2.82746E-14
GO:0022900	electron transport chain	15.83390075	1.46588E-16	4.67523E-14	3.93687E-14
GO:0010257	NADH dehydrogenase complex assembly	15.82061461	1.51142E-16	4.67523E-14	3.93687E-14
GO:0032981	mitochondrial respiratory chain complex I assembly	15.82061461	1.51142E-16	4.67523E-14	3.93687E-14
GO:0032543	mitochondrial translation	14.41932732	3.80779E-15	8.32636E-13	7.01138E-13
GO:0140053	mitochondrial gene expression	12.23398576	5.83464E-13	1.07649E-10	9.06481E-11
GO:0006839	mitochondrial transport	11.46799237	3.40414E-12	5.7423E-10	4.83542E-10
GO:0006120	mitochondrial electron transport, NADH to ubiquinone	11.09890575	7.96332E-12	1.27069E-09	1.07001E-09
GO:0006735	NADH regeneration	1.499717873	0.031643326	0.179636728	0.151266629
GO:2001169	regulation of ATP biosynthetic process	1.639911579	0.022913341	0.143002501	0.12041806

Table S25: Mitochondrial related GO/CC terms upregulated in patient DA neurons with NR, related to Figure 8B.

ID	Description	Foldchange	pvalue	p.adjust	qvalue
GO:0005743	mitochondrial inner membrane	39.95388565	1.11202E-40	7.9621E-38	5.27919E-38
GO:0098798	mitochondrial protein complex	29.92242922	1.19556E-30	4.2801E-28	2.83788E-28
GO:0098800	inner mitochondrial membrane protein complex	21.72731473	1.87364E-22	1.6769E-20	1.11186E-20
GO:0005746	mitochondrial respirasome	15.89522809	1.27283E-16	6.50964E-15	4.31615E-15
GO:0070469	respirasome	14.24088283	5.74271E-15	2.28432E-13	1.5146E-13
GO:0098803	respiratory chain complex	13.49474091	3.2008E-14	1.14589E-12	7.5977E-13
GO:0005759	mitochondrial matrix	11.13494316	7.3292E-12	1.9436E-10	1.28868E-10
GO:0000313	organellar ribosome	10.86698678	1.35835E-11	3.35373E-10	2.22366E-10
GO:0005761	mitochondrial ribosome	10.86698678	1.35835E-11	3.35373E-10	2.22366E-10
GO:0048786	presynaptic active zone	9.884865507	1.30357E-10	3.11119E-09	2.06284E-09
GO:0005747	mitochondrial respiratory chain complex I	9.561545774	2.74444E-10	5.95461E-09	3.94815E-09
GO:0030964	NADH dehydrogenase complex	9.561545774	2.74444E-10	5.95461E-09	3.94815E-09
GO:0045271	respiratory chain complex I	9.561545774	2.74444E-10	5.95461E-09	3.94815E-09
GO:0098573	intrinsic component of mitochondrial membrane	6.40818559	3.90674E-07	4.66204E-06	3.09112E-06
GO:0005758	mitochondrial intermembrane space	5.511984773	3.0762E-06	3.23906E-05	2.14763E-05
GO:0031304	intrinsic component of mitochondrial inner membrane	5.090559638	8.11784E-06	7.97831E-05	5.28994E-05
GO:0005763	mitochondrial small ribosomal subunit	4.45037349	3.54508E-05	0.000305817	0.000202769
GO:0005753	mitochondrial proton-transporting ATP synthase complex	4.056992127	8.77017E-05	0.000647365	GO:0005753
GO:0045277	respiratory chain complex IV	1.615112143	0.024259836	0.07423095	GO:0045277

Table S26: Mitochondrial related GO/MF terms upregulated in patient DA neurons with NR, related to Figure 8C.

ID	Description	Foldchange	pvalue	p.adjust	qvalue
GO:0016655	oxidoreductase activity, acting on NAD(P)H, quinone or similar compound as acceptor	10.82566767	1.494E-11	1.6045E-08	1.37757E-08
GO:0003954	NADH dehydrogenase activity	10.15094567	7.064E-11	1.8967E-08	1.62845E-08
GO:0008137	NADH dehydrogenase (ubiquinone) activity	10.15094567	7.064E-11	1.8967E-08	1.62845E-08
GO:0050136	NADH dehydrogenase (quinone) activity	10.15094567	7.064E-11	1.8967E-08	1.62845E-08
GO:0016651	oxidoreductase activity, acting on NAD(P)H	9.624022613	2.377E-10	5.1052E-08	4.38317E-08
GO:0015078	proton transmembrane transporter activity	6.970106792	1.071E-07	8.2181E-06	7.05579E-06
GO:0009055	electron transfer activity	6.575075352	2.66E-07	1.7857E-05	1.53315E-05
GO:0044389	ubiquitin-like protein ligase binding	6.525998157	2.979E-07	1.8817E-05	1.6156E-05
GO:0042625	ATPase-coupled ion transmembrane transporter activity	4.975468936	1.058E-05	0.00039187	0.000336445
GO:0001671	ATPase activator activity	4.87669575	1.328E-05	0.00047554	0.000408285
GO:0046933	proton-transporting ATP synthase activity, rotational mechanism	2.945177238	0.0011345	0.01818663	0.015614514
GO:0004129	cytochrome-c oxidase activity	2.894459005	0.0012751	0.01928799	0.016560107
GO:0015002	heme-copper terminal oxidase activity	2.894459005	0.0012751	0.01928799	0.016560107
GO:0016676	oxidoreductase activity, acting on a heme group of donors, oxygen as acceptor	2.894459005	0.0012751	0.01928799	0.016560107

Table S22: Neural related GO/BP terms upregulated in patient DA neurons with NR, related to Figure 8D.

ID	Description	Foldchange	pvalue	p.adjust	qvalue
GO:0099177 +A2:B1168	regulation of trans-synaptic signaling	23.98876357	1.02621E-24	6.05875E-21	5.10189E-21
GO:0050804	modulation of chemical synaptic transmission	23.57229488	2.67735E-24	7.90354E-21	6.65533E-21
GO:0099003	vesicle-mediated transport in synapse	23.01464402	9.66843E-24	1.90275E-20	1.60225E-20
GO:0099504	synaptic vesicle cycle	20.82106666	1.50985E-21	2.22854E-18	1.87658E-18
GO:0050808	synapse organization	17.67648023	2.1063E-18	1.24873E-15	1.05152E-15
GO:0001505	regulation of neurotransmitter levels	16.04460293	9.02396E-17	3.55183E-14	2.99089E-14
GO:0007269	neurotransmitter secretion	15.80031329	1.58375E-16	4.67523E-14	3.93687E-14
GO:0099643	signal release from synapse	15.80031329	1.58375E-16	4.67523E-14	3.93687E-14
GO:0006836	neurotransmitter transport	15.60326771	2.49306E-16	7.00905E-14	5.90211E-14
GO:0048167	regulation of synaptic plasticity	15.53106585	2.94398E-16	7.90056E-14	6.65282E-14
GO:0007416	synapse assembly	12.30240771	4.98416E-13	9.49242E-11	7.99328E-11
GO:0042391	regulation of membrane potential	10.74302874	1.80705E-11	2.7356E-09	2.30357E-09
GO:1990138	neuron projection extension	9.304447142	4.96081E-10	6.65651E-08	5.60524E-08
GO:0045666	positive regulation of neuron differentiation	9.072302686	8.46637E-10	1.08664E-07	9.15027E-08
GO:0010769	regulation of cell morphogenesis involved in differentiation	8.967958353	1.07657E-09	1.32418E-07	1.11505E-07

Table S28: Neural related GO/CC terms upregulated in patient DA neurons with NR, related to Figure 8E.

ID	Description	Foldchange	pvalue	p.adjust	qvalue
GO:0097060	synaptic membrane	25.83638728	1.45751E-26	3.4786E-24	2.30645E-24
GO:0098984	neuron to neuron synapse	22.96154855	1.09258E-23	1.95571E-21	1.29671E-21
GO:0032279	asymmetric synapse	22.43534677	3.66989E-23	5.25528E-21	3.48447E-21
GO:0099572	postsynaptic specialization	22.08862117	8.15415E-23	9.73062E-21	6.45179E-21
GO:0014069	postsynaptic density	21.99876551	1.00285E-22	1.02577E-20	6.80126E-21
GO:0098978	glutamatergic synapse	19.44004715	3.63039E-20	2.88817E-18	GO:0098978
GO:0150034	distal axon	19.05111166	8.88973E-20	6.36504E-18	GO:0150034
GO:0045211	postsynaptic membrane	18.98347826	1.03878E-19	6.76148E-18	GO:0045211
GO:0008021	synaptic vesicle	17.68453353	2.0676E-18	1.23367E-16	GO:0008021
GO:0070382	exocytic vesicle	17.1653502	6.8336E-18	3.76374E-16	GO:0070382
GO:0098982	GABA-ergic synapse	15.11477462	7.6776E-16	3.66477E-14	GO:0098982
GO:0030426	growth cone	14.34930369	4.474E-15	1.88434E-13	1.2494E-13
GO:0030427	site of polarized growth	14.07316958	8.44949E-15	3.18412E-13	2.1112E-13
GO:0042734	presynaptic membrane	12.53036088	2.94876E-13	1.00539E-11	6.66611E-12
GO:0099634	postsynaptic specialization membrane	9.007945568	9.81871E-10	2.0677E-08	1.37097E-08

Table S29: Neural related GO/MF terms upregulated in patient DA neurons with NR, related to Figure 8F.

ID	Description	Foldchange	pvalue	p.adjust	qvalue
GO:0035254	glutamate receptor binding	5.426706865	3.744E-06	0.00015464	0.00013277
GO:0035255	ionotropic glutamate receptor binding	1.771219316	0.0169348	0.12125334	0.104104601
GO:0098918	structural constituent of synapse	1.758947604	0.0174202	0.1239024	0.106379011
GO:0035091	phosphatidylinositol binding	1.69157712	0.0203434	0.14374196	0.123412683
GO:0004970	ionotropic glutamate receptor activity	1.640886415	0.022862	0.15739585	0.135135511
GO:0099529	neurotransmitter receptor activity involved in regulation of postsynaptic membrane potential	1.414191105	0.0385309	0.22266163	0.191170817
GO:0008066	glutamate receptor activity	1.384965358	0.041213	0.23174243	0.198967332

Table S30: Upregulated mitochondrial oxidative phosphorylation-associated DEGs in NR-treated DA2 neurons, related to Figure 9C.

names	scores	logfoldchanges	pvals	pvals_adj
CYCS	8.394803	0.54445	4.67E-17	1.69E-13
MT-CO3	7.318803	0.302521	2.5E-13	4.42E-10
MT-ND3	6.929154	0.322183	4.23E-12	6.04E-09
MT-ND4	5.34224	0.245581	9.18E-08	5.08E-05
MT-ATP6	4.708699	0.195912	2.49E-06	0.000953
MT-CO2	4.651298	0.259459	3.3E-06	0.001164
COX7B	4.322026	0.325848	1.55E-05	0.004276
COX7A2	4.269642	0.176869	1.96E-05	0.005146
NDUFA5	4.057129	0.245565	4.97E-05	0.011879
SLC25A5	3.898191	0.344171	9.69E-05	0.019736
LYRM2	3.823272	0.503013	0.000132	0.025555
NDUFB9	3.646816	0.224629	0.000266	0.04523

Table S31: Upregulated mitochondrial stress and proteostasis-associated DEGs in NR-treated DA2 neurons, related to Figure 9D.

names	scores	logfoldchanges	pvals	pvals_adj
HSP90AA1	8.631042	0.321552	6.08E-18	2.5E-14
HSP90AB1	8.386044	0.331287	5.03E-17	1.69E-13
HSPD1	6.65907	0.413874	2.76E-11	3.4E-08
ATF4	5.903923	0.78587	3.55E-09	2.76E-06
NFE2L1	4.428665	0.404113	9.48E-06	0.002834
KLF6	4.334867	0.723237	1.46E-05	0.004095
CRYAB	3.663654	2.131991	0.000249	0.042863

Table S32: Upregulated neuronal/synapti-associated DEGs in NR-treated DA2 neurons, related to Figure 9E.

names	scores	logfoldchanges	pvals	pvals_adj
LGI2	7.648585	1.348754	2.03E-14	3.96E-11
KIF1A	5.917699	0.42903	3.26E-09	2.67E-06
NEFL	5.214682	0.499888	1.84E-07	9.61E-05
GPRIN3	4.944172	0.797855	7.65E-07	0.000337
CADM2	4.553332	0.512024	5.28E-06	0.001702
CNTN1	4.421522	0.431985	9.8E-06	0.002906
DCX	4.403664	0.223461	1.06E-05	0.003082
PCDH9	4.011464	0.592461	6.03E-05	0.013555
MAPT	3.986377	0.198211	6.71E-05	0.014801
MAP2	3.799461	0.148888	0.000145	0.027421

Table S25: Mitochondrial related GO/BP terms upregulated in patient DA2 neurons with NR, related to Figure 10A.

ID	Description	Foldchange	pvalue	p.adjust	qvalue
GO:0072599	establishment of protein localization to endoplasmic reticulum	6.37747206	4.19E-07	0.000129215	0.00010284
GO:0034643	establishment of mitochondrion localization, microtubule-mediated	4.067031136	8.57E-05	0.00310696	0.002472762
GO:0047497	mitochondrion transport along microtubule	4.067031136	8.57E-05	0.00310696	0.002472762
GO:0046034	ATP metabolic process	3.778076975	0.000167	0.00497128	0.003956534
GO:0006119	oxidative phosphorylation	3.609167796	0.000246	0.006590525	0.005245256
GO:0042775	mitochondrial ATP synthesis coupled electron transport	3.309710738	0.00049	0.011470942	0.009129474
GO:0042773	ATP synthesis coupled electron transport	3.292839304	0.00051	0.011776266	0.009372475
GO:0022900	electron transport chain	3.189440899	0.000646	0.013899448	0.011062269
GO:0051646	mitochondrion localization	3.160903155	0.00069	0.014385014	0.01144872
GO:0019896	axonal transport of mitochondrion	2.908644067	0.001234	0.020934681	0.016661458
GO:0006123	mitochondrial electron transport, cytochrome c to oxygen	2.552858634	0.0028	0.036978581	0.02943045
GO:0019646	aerobic electron transport chain	2.552858634	0.0028	0.036978581	0.02943045
GO:0015980	energy derivation by oxidation of organic compounds	2.403197557	0.003952	0.04683977	0.037278756
GO:0072593	reactive oxygen species metabolic process	2.338425563	0.004587	0.051684094	0.041134248
GO:0045333	cellular respiration	2.266242114	0.005417	0.057563282	0.045813366
GO:2000379	positive regulation of reactive oxygen species metabolic process	2.126608722	0.007471	0.07048101	0.056094305
GO:0006120	mitochondrial electron transport, NADH to ubiquinone	1.739056998	0.018237	0.126289909	0.100511396
GO:0010257	NADH dehydrogenase complex assembly	1.603157581	0.024937	0.15524688	0.12355762
GO:0032981	mitochondrial respiratory chain complex I assembly	1.603157581	0.024937	0.15524688	0.12355762

Table S34: Mitochondrial related GO/CC terms upregulated in patient DA2 neurons with NR, related to Figure 10B.

ID	Description	Foldchange	pvalue	p.adjust
GO:0070469	respirasome	4.48116678	3.3E-05	0.000482154
GO:0005746	mitochondrial respirasome	3.51086523	0.000308	0.003105415
GO:0044309	neuron spine	3.33646082	0.000461	0.004205059
GO:0044233	mitochondria-associated endoplasmic reticulum membrane	2.82142739	0.001509	0.01129512
GO:0005743	mitochondrial inner membrane	2.7083569	0.001957	0.013143807
GO:0098803	respiratory chain complex	2.43662014	0.003659	0.019605198
GO:0005747	mitochondrial respiratory chain complex I	1.84696907	0.014224	0.050652389
GO:0030964	NADH dehydrogenase complex	1.84696907	0.014224	0.050652389
GO:0045271	respiratory chain complex I	1.84696907	0.014224	0.050652389

Table S35: Mitochondrial related GO/MF terms upregulated in patient DA2 neurons with NR, related to Figure 10C.

ID	Description	Foldchange	pvalue	p.adjust	qvalue
GO:0051082	unfolded protein binding	5.234410227	5.8E-06	0.00070348	0.000466578
GO:0004129	cytochrome-c oxidase activity	2.293740017	0.00508	0.0376634505	0.024980041
GO:0015002	heme-copper terminal oxidase activity	2.293740017	0.00508	0.0376634505	0.024980041
GO:0016676	oxidoreductase activity, acting on a heme group of donors, oxygen as acceptor	2.293740017	0.00508	0.0376634505	0.024980041
GO:0016675	oxidoreductase activity, acting on a heme group of donors	2.263772438	0.00545	0.0376634505	0.024980041
GO:0031072	heat shock protein binding	1.897947322	0.01265	0.0606869601	0.040250231
GO:0003954	NADH dehydrogenase activity	1.87544596	0.01332	0.0606869601	0.040250231
GO:0008137	NADH dehydrogenase (ubiquinone) activity	1.87544596	0.01332	0.0606869601	0.040250231
GO:0050136	NADH dehydrogenase (quinone) activity	1.87544596	0.01332	0.0606869601	0.040250231
GO:0016655	oxidoreductase activity, acting on NAD(P)H, quinone or similar compound as acceptor	1.643823432	0.02271	0.0931023003	0.061749495

Table S36: Neural related GO/BP terms upregulated in patient DA2 neurons with NR, related to Figure 10D

ID	Description	Foldchange	pvalue	p.adjust
GO:0098930	axonal transport	5.55327133	2.8E-06	0.000470189
GO:1901214	regulation of neuron death	5.501461102	3.15E-06	0.000485618
GO:0008088	axo-dendritic transport	5.183820232	6.55E-06	0.000737126
GO:0048675	axon extension	5.16225021	6.88E-06	0.000737126
GO:0070997	neuron death	5.098365051	7.97E-06	0.000737126
GO:0021954	central nervous system neuron development	4.84596042	1.43E-05	0.001054475
GO:1990138	neuron projection extension	4.335816583	4.62E-05	0.002245622
GO:0008090	retrograde axonal transport	4.254826336	5.56E-05	0.002519451
GO:0007409	axonogenesis	4.104465384	7.86E-05	0.00310696
GO:0048167	regulation of synaptic plasticity	4.083270278	8.26E-05	0.00310696
GO:0021953	central nervous system neuron differentiation	4.070762156	8.5E-05	0.00310696
GO:0050772	positive regulation of axonogenesis	3.50882894	0.00031	0.008069556
GO:0030516	regulation of axon extension	3.309710738	0.00049	0.011470942
GO:1901216	positive regulation of neuron death	3.309710738	0.00049	0.011470942
GO:0051412	response to corticosterone	2.595269117	0.002539	0.035039911

Table S37: Neural related GO/CC terms upregulated in patient DA2 with NR, related to Figure 10E.

ID	Description	Foldchange	pvalue	p.adjust
GO:0014069	postsynaptic density	6.43518166	3.67E-07	2.52031E-05
GO:0032279	asymmetric synapse	6.3649597	4.32E-07	2.52031E-05
GO:0099572	postsynaptic specialization	6.16230262	6.88E-07	2.9905E-05
GO:0120111	neuron projection cytoplasm	6.13145309	7.39E-07	2.9905E-05
GO:0098984	neuron to neuron synapse	6.08654871	8.19E-07	2.9905E-05
GO:0030055	cell-substrate junction	5.54300273	2.86E-06	7.79099E-05
GO:0030426	growth cone	5.33202637	4.66E-06	0.000104571
GO:0030427	site of polarized growth	5.22537301	5.95E-06	0.000124131
GO:0098936	intrinsic component of postsynaptic membrane	2.99391482	0.001014	0.008460577
GO:0044232	organelle membrane contact site	2.36649605	0.0043	0.021283095
GO:0044295	axonal growth cone	2.30530904	0.004951	0.023317508
GO:0099055	integral component of postsynaptic membrane	2.05184912	0.008875	0.036498531
GO:0097060	synaptic membrane	1.87789956	0.013246	0.050219714
GO:0099699	integral component of synaptic membrane	1.73230633	0.018522	0.065162603
GO:0045211	postsynaptic membrane	1.7238228	0.018888	0.065656959
GO:0032589	neuron projection membrane	1.68374786	0.020713	0.06826707
GO:0098948	intrinsic component of postsynaptic specialization membrane	1.49600187	0.031915	0.093192503
GO:0098992	neuronal dense core vesicle	1.36943967	0.042713	0.113383666

Table S38: Neural related GO/MF terms upregulated in patient DA2 neurons with NR, related to Figure 10F.

ID	Description	Foldchange	pvalue	p.adjust
GO:0035255	ionotropic glutamate receptor binding	2.293740017	0.00508	0.037663455
GO:0051087	chaperone binding	2.133622547	0.00735	0.041573632
GO:0035254	glutamate receptor binding	1.87544596	0.01332	0.060686961
GO:0097110	scaffold protein binding	1.657244146	0.02202	0.091799211
GO:0099186	structural constituent of postsynapse	1.34836718	0.04484	0.126779395
GO:1902282	voltage-gated potassium channel activity involved in ventricular cardiac muscle cell action potential repolarization	1.34836718	0.04484	0.126779395
GO:0015271	outward rectifier potassium channel activity	1.314416991	0.04848	0.132518224

Table S39: Upregulated mitochondrial oxidative phosphorylation-associated DEGs in NR-treated VMNs, related to Figure 11C.

names	scores	logfoldchanges	pvals	pvals_adj
ATP5MC2	4.653102	1.816258	3.27E-06	0.001143
MT-ATP6	4.473223	1.709983	7.7E-06	0.002284
MT-CO3	4.262798	1.579026	2.02E-05	0.00509
COX6C	4.122797	1.451444	3.74E-05	0.00816
NDUFAF3	4.113464	2.340884	3.9E-05	0.00835
NDUFS2	4.107525	1.859852	4E-05	0.00847
NDUFB1	4.03201	1.32041	5.53E-05	0.010961
NDUFA3	3.993828	1.476686	6.5E-05	0.012485
ATP5PD	3.961161	1.493884	7.46E-05	0.013962
MT-ND4	3.9251	1.467071	8.67E-05	0.015828
MT-ND1	3.859343	1.656973	0.000114	0.019014
MT-CO1	3.858918	1.369173	0.000114	0.019014
NDUFB10	3.83007	1.572517	0.000128	0.020644
MT-ND5	3.758373	1.520279	0.000171	0.025872
ATP5F1D	3.72613	1.475517	0.000194	0.027825
UQCRFS1	3.687524	1.389829	0.000226	0.0312
MT-CYB	3.682857	1.396013	0.000231	0.031595
MT-ND4L	3.682433	1.605806	0.000231	0.031595
CYC1	3.669706	1.706216	0.000243	0.032967
UQCRQ	3.608615	1.164808	0.000308	0.039207
NDUFS8	3.602675	1.550481	0.000315	0.039841

Table S40: Upregulated neuromuscular maturation-associated DEGs in NR-treated VMNs, related to Figure 11D.

names	scores	logfoldchanges	pvals	pvals_adj
TTN	9.444508	5.760737	3.57E-21	1.32E-16
TNNT3	8.014807	4.90611	1.1E-15	8.18E-12
ACTA1	6.85238	4.112558	7.26E-12	1.79E-08
MYH3	5.92965	3.413744	3.04E-09	3.31E-06
MEF2C	5.811286	3.297592	6.2E-09	5.89E-06
CKM	5.661529	3.939511	1.5E-08	1.21E-05
CHRNA1	4.647162	3.218509	3.37E-06	0.001166
MYOG	4.427404	2.924505	9.54E-06	0.00274
CHRNB1	4.011222	3.198377	6.04E-05	0.011721
DESI2	3.832191	2.707193	0.000127	0.020591
RAPSN	3.620493	2.632437	0.000294	0.038105
DCLK1	3.531402	1.864886	0.000413	0.049904

Table S41: Mitochondrial related GO/BP terms upregulated in patient VMNs with NR, related to Figure 12A.

ID	Description	Foldchange	pvalue	p.adjust	qvalue
GO:0071453	cellular response to oxygen levels	4.790237365	1.62092E-05	0.000577621	0.000506451
GO:0042775	mitochondrial ATP synthesis coupled electron transport	4.75565407	1.75528E-05	0.000618225	0.000542052
GO:0042773	ATP synthesis coupled electron transport	4.7195871	1.90727E-05	0.000664038	0.00058222
GO:0071456	cellular response to hypoxia	4.706491498	1.96566E-05	0.000676589	0.000593225
GO:0061418	regulation of transcription from RNA polymerase II promoter in response to hypoxia	4.645536083	2.26185E-05	0.000769792	0.000674944
GO:0042776	mitochondrial ATP synthesis coupled proton transport	4.571459002	2.68251E-05	0.000892892	0.000782876
GO:0036294	cellular response to decreased oxygen levels	4.49240575	3.21806E-05	0.001048119	0.000918978
GO:0070482	response to oxygen levels	4.388180266	4.09091E-05	0.00131823	0.001155808
GO:0015986	ATP synthesis coupled proton transport	4.309139921	4.9075E-05	0.001516817	0.001329927
GO:0010257	NADH dehydrogenase complex assembly	4.23163315	5.86633E-05	0.001742071	0.001527427
GO:0032981	mitochondrial respiratory chain complex I assembly	4.23163315	5.86633E-05	0.001742071	0.001527427
GO:0022904	respiratory electron transport chain	4.138062487	7.27675E-05	0.002119354	0.001858223
GO:0009060	aerobic respiration	3.431515827	0.000370241	0.008185833	0.007177236
GO:1990542	mitochondrial transmembrane transport	3.041153307	0.000909592	0.016597317	0.01455232
GO:0006839	mitochondrial transport	3.007616582	0.000982615	0.017507888	0.015350698
GO:0043462	regulation of ATPase activity	2.881520727	0.001313649	0.021625229	0.018960732
GO:0006754	ATP biosynthetic process	2.861946398	0.001374212	0.022259288	0.019516666
GO:0006122	mitochondrial electron transport, ubiquinol to cytochrome c	2.854471904	0.001398067	0.022406064	0.019645358
GO:0006120	mitochondrial electron transport, NADH to ubiquinone	2.82592633	0.001493048	0.023677703	0.020760315
GO:0070125	mitochondrial translational elongation	2.640992588	0.002285638	0.03281136	0.028768592
GO:0070126	mitochondrial translational termination	2.616164799	0.002420111	0.034147748	0.02994032
GO:0006734	NADH metabolic process	2.461650385	0.003454217	0.04471292	0.039203731
GO:0034249	negative regulation of cellular amide metabolic process	2.430763842	0.003708823	0.047004294	0.041212779
GO:0032543	mitochondrial translation	2.325023344	0.004731258	0.058028016	0.050878242
GO:0006979	response to oxidative stress	2.324964595	0.004731898	0.058028016	0.050878242
GO:0006735	NADH regeneration	2.104976816	0.007852776	0.084950204	0.074483281
GO:0140053	mitochondrial gene expression	1.868282606	0.013543078	0.126611064	0.111011004
GO:0019674	NAD metabolic process	1.389642309	0.040771594	0.261093358	0.228923404

Table S42: Mitochondrial related GO/CC terms upregulated in patient VMNs with NR, related to Figure 12B.

ID	Description	Foldchange	pvalue	p.adjust	qvalue
GO:0098798	mitochondrial protein complex	11.73205595	1.8533E-12	3.609E-11	2.59E-11
GO:0005743	mitochondrial inner membrane	9.530304822	2.9491E-10	5.456E-09	3.91E-09
GO:0098800	inner mitochondrial membrane protein complex	8.621099514	2.3928E-09	4.024E-08	2.89E-08
GO:1990204	oxidoreductase complex	6.106258703	7.8296E-07	1.114E-05	7.99E-06
GO:0098803	respiratory chain complex	5.303625469	4.9702E-06	6.341E-05	4.55E-05
GO:0005746	mitochondrial respirasome	5.137642942	7.2838E-06	8.422E-05	6.04E-05
GO:0033017	sarcoplasmic reticulum membrane	4.579346817	2.6342E-05	0.0002707	0.000194
GO:0005747	mitochondrial respiratory chain complex I	4.016650238	9.6239E-05	0.0007913	0.000567
GO:0030964	NADH dehydrogenase complex	4.016650238	9.6239E-05	0.0007913	0.000567
GO:0045271	respiratory chain complex I	4.016650238	9.6239E-05	0.0007913	0.000567
GO:0005759	mitochondrial matrix	3.096078907	0.00080153	0.005492	0.003937
GO:0005750	mitochondrial respiratory chain complex III	2.974251793	0.00106108	0.0068877	0.004938
GO:0045275	respiratory chain complex III	2.974251793	0.00106108	0.0068877	0.004938
GO:0042645	mitochondrial nucleoid	2.420267599	0.00379955	0.0209826	0.015043
GO:0045259	proton-transporting ATP synthase complex	2.330224549	0.00467493	0.0243623	0.017466
GO:0000276	mitochondrial proton-transporting ATP synthase complex, coupling factor F(o)	1.87676023	0.01328128	0.0571404	0.040965
GO:0005744	TIM23 mitochondrial import inner membrane translocase complex	1.745780731	0.0179564	0.0706794	0.050672
GO:0045263	proton-transporting ATP synthase complex, coupling factor F(o)	1.745780731	0.0179564	0.0706794	0.050672
GO:0005761	mitochondrial ribosome	1.383727459	0.04133068	0.1390214	0.099668

Table S43: Mitochondrial related GO/MF terms upregulated in patient VMN with NR, related to Figure 12C.

ID	Description	Foldchange	pvalue	p.adjust	qvalue
GO:0003954	NADH dehydrogenase activity	3.148684612	0.000710093	0.013522645	0.011764474
GO:0008137	NADH dehydrogenase (ubiquinone) activity	3.148684612	0.000710093	0.013522645	0.011764474
GO:0050136	NADH dehydrogenase (quinone) activity	3.148684612	0.000710093	0.013522645	0.011764474

Table S44: Neural related GO/BP terms upregulated in patient VMN with NR, related to Figure 12D.

ID	Description	Foldchange	pvalue	p.adjust	qvalue
GO:0007274	neuromuscular synaptic transmission	3.42647695	0.000374561	0.008221352	0.007208379
GO:0008064	regulation of actin polymerization or depolymerization	2.04876069	0.008937979	0.093355645	0.081853067
GO:0007271	synaptic transmission, cholinergic	1.977145964	0.010540326	0.104335447	0.091480021
GO:0010644	cell communication by electrical coupling	1.937791024	0.011540084	0.11179018	0.098016238
GO:0007528	neuromuscular junction development	1.389642309	0.040771594	0.261093358	0.228923404
GO:0045104	intermediate filament cytoskeleton organization	1.389642309	0.040771594	0.261093358	0.228923404
GO:0007164	establishment of tissue polarity	1.380360899	0.041652311	0.264546206	0.231950818

Table S45: Neural related GO/CC terms upregulated in patient VMN with NR, related to Figure 12E.

ID	Description	Foldchange	pvalue	p.adjust	qvalue
GO:0030055	cell-substrate junction	31.75103962	1.774E-32	8.205E-31	5.88E-31
GO:0005925	focal adhesion	30.07829251	8.3504E-31	3.433E-29	2.46E-29
GO:0099572	postsynaptic specialization	8.939556779	1.1493E-09	2.025E-08	1.45E-08
GO:0014069	postsynaptic density	7.399016783	3.9901E-08	6.419E-07	4.6E-07
GO:0032279	asymmetric synapse	7.268029916	5.3947E-08	8.317E-07	5.96E-07
GO:0098984	neuron to neuron synapse	6.753022646	1.7659E-07	2.614E-06	1.87E-06
GO:0031594	neuromuscular junction	4.755818154	1.7546E-05	0.0001909	0.000137
GO:0030864	cortical actin cytoskeleton	2.159474687	0.00692668	0.0336924	0.024155
GO:0005892	acetylcholine-gated channel complex	1.634137349	0.02322002	0.0859141	0.061594
GO:0036464	cytoplasmic ribonucleoprotein granule	1.6170163	0.0241537	0.0884839	0.063437
GO:0044291	cell-cell contact zone	1.605980185	0.02477535	0.0898714	0.064431

Table S46: Neural related GO/MF terms upregulated in patient VMN with NR, related to Figure 12F.

ID	Description	Foldchange	pvalue	p.adjust	qvalue
GO:0022848	acetylcholine-gated cation-selective channel activity	1.602314295	0.024985365	0.168362924	0.146472911
GO:0042165	neurotransmitter binding	1.381256166	0.041566536	0.249399216	0.216973123
GO:0000062	fatty-acyl-CoA binding	1.344241805	0.045264549	0.264344964	0.229975672
GO:0015464	acetylcholine receptor activity	1.344241805	0.045264549	0.264344964	0.229975672
